# Causality-enhanced and symmetry-preserving PINNs for damped elastodynamic PDEs

**DOI:** 10.1371/journal.pone.0360636

**Published:** 2026-10-27

**Authors:** Muhammad Israr, Shahbaz Ahmad, F. D. Zaman, Iftikhar Ali

**Affiliations:** 1 Department of Sciences and Humanities, National University of Computer and Emerging Sciences, Peshawar, Khyber Pakhtunkhwa, Pakistan; 2 Abdus Salam School of Mathematical Sciences, GC University Lahore, Lahore, Pakistan; 3 Department of Mathematics, University of Hafr Al Batin, Hafar Al-Batin, Saudi Arabia; Government College University Faisalabad, PAKISTAN

## Abstract

This study develops a causality-enhanced and symmetry-preserving Physics-Informed Neural Network (S-PINN) framework for nonlinear damped elastodynamic partial differential equations. The governing nonlinear elastic wave equation with viscous damping is reduced through Lie symmetry analysis into four invariant ordinary differential equation forms, including scaling, translation, damped, and generalized damped symmetries. The proposed framework combines symmetry-aware solution reconstruction, composite residual and symmetry losses, and causality-based temporal weighting to improve the training stability and physical consistency of PINNs. Numerical experiments are performed using a fully connected neural network with four hidden layers, 128 neurons per layer, the tanh activation function, and the L-BFGS optimizer. The S-PINN solutions are compared with conventional PINNs and exact analytical references for all four symmetry-reduced cases. The results show that the proposed S-PINN framework provides faster convergence, lower loss, and improved solution accuracy. In Case IV, S-PINNs achieve a 35% faster convergence rate during the first 500 epochs and reduce the final loss from 3.8 for PINNs to 2.4. The S-PINN loss stabilizes after approximately 1000 epochs, whereas the PINN loss continues to oscillate with fluctuations up to 15%. The gradient norm remains within 0.5–1.5 for S-PINNs, compared with 0.2–2.5 for PINNs. At α=0.4, the S-PINN solution attains 98% agreement with the exact solution, and its maximum absolute error remains below 0.05, compared with 0.18 for the standard PINN. Contour comparisons further show less than 5% distortion for S-PINNs, while PINNs exhibit distortions up to 18%. These findings demonstrate that embedding Lie symmetries and causal temporal learning into PINNs constrains the solution space to physically admissible manifolds and improves the reliability of neural solvers for nonlinear wave propagation in computational mechanics.

## 1 Introduction

Nonlinear elastic wave propagation is a central topic in computational mechanics, material science, nondestructive testing, seismic modeling, biomedical ultrasound, and soft-material analysis [1,2]. In contrast to linear wave models, nonlinear elastodynamic equations can describe amplitude-dependent wave speed, harmonic generation, wave steepening, and other complex phenomena observed in hyperelastic polymers, biological tissues, and architected metamaterials [3,4]. When viscous damping is included, the mathematical model becomes more realistic but also more difficult to solve because the solution depends simultaneously on nonlinear spatial deformation, temporal dissipation, and material parameters [5,6].

Classical numerical methods, including finite difference, finite volume, and finite element schemes, remain powerful tools for wave propagation problems [7,8]. However, their performance deteriorate for strongly nonlinear, multiscale, or long-time simulations because of mesh dependence, stability restrictions, and high computational cost [9,10]. Physics-Informed Neural Networks (PINNs) have recently emerged as mesh-free scientific machine-learning solvers that approximate the unknown field while enforcing the governing differential equation through the loss function [11,12]. Despite their flexibility, standard PINNs are known to face training difficulties, including gradient imbalance, stiffness, slow convergence, and reduced accuracy for time-dependent nonlinear systems [13,14].

Several recent studies have attempted to improve PINN training by modifying the loss function, network structure, or temporal learning strategy [15,16]. Causality-based PINNs introduce time-aware weighting to ensure that earlier physical states are learned before later ones, which improves stability for evolutionary systems [17,18]. Similarly, symmetry-informed learning has attracted increasing attention because Lie point symmetries can reduce differential equations, constrain admissible solutions, and improve the generalization ability of neural solvers [19,20]. However, most existing PINN-based studies treat causality and symmetry preservation separately. Moreover, their application to nonlinear damped elastodynamic PDEs remains limited, particularly when exact Lie reductions, symmetry losses, and causal temporal training are combined in a single computational framework [21,22].

Recent investigations have further shown that the geometry of the computational domain can significantly influence the optimization behavior of physics-informed neural models. In particular, hyperparameter studies on symmetric and asymmetric domains have demonstrated that network depth, width, activation function, optimizer choice, learning rate, collocation-point distribution, and loss weighting may produce distinct convergence patterns depending on the underlying geometric structure of the problem [23]. These observations are especially relevant for symmetry-preserving PINNs, since the use of invariant variables and reduced computational domains changes the effective search space explored by the neural optimizer. Therefore, incorporating symmetry information is not only a mathematical reduction strategy but also a mechanism for improving the conditioning of the learning problem [24].

In addition to purely neural formulations, transformation-based analytical and semi-analytical methods provide useful insight into the nonlinear structure of differential systems. Comparative studies between PINNs and symbolic transformation approaches, for example in nonlinear benchmark problems such as the Troesch equation, have shown that symbolic or continuous transformation methods can preserve the intrinsic nonlinear dynamics of the governing system, whereas PINNs offer flexibility for mesh-free approximation and data-driven generalization [25]. This comparison highlights the importance of hybrid frameworks that combine the interpretability and structure preservation of analytical transformations with the adaptive approximation capability of deep neural networks [26].

Hybridization of physics-informed learning with classical algebraic-differential solvers has also emerged as an effective direction for improving convergence and reducing initialization sensitivity [27,28]. For instance, the integration of the Differential Transform Method (DTM) with PINN architectures for integral–algebraic equation systems has demonstrated that transformation-based preliminary representations can guide neural training toward physically meaningful solution branches. Motivated by these developments, the present study adopts Lie symmetry reductions as a structure-preserving transformation and embeds the resulting invariant formulation into a causality-enhanced S-PINN framework. This enables the proposed model to benefit simultaneously from analytical reduction, symmetry-constrained learning, and temporally ordered optimization [16].

This observation defines the main research gap addressed in the present work. Standard PINNs do not explicitly preserve the invariant structure of nonlinear elastic wave equations, and purely causal PINNs do not directly exploit the Lie symmetry properties of the governing model [23,29]. As a result, the learned solution satisfy the residual locally while still deviating from the physically admissible solution manifold. Therefore, a directed need exists for a neural solver that simultaneously incorporates the governing PDE, the reduced symmetry structure, and the temporal-causality constraint [30,31]. To meet this need, we develop a causality-enhanced and symmetry-preserving PINN framework for nonlinear elastic wave equations with viscous damping [32].

The motivation for the present study arises from the need to develop a computationally efficient and physically consistent framework for nonlinear elastic wave propagation in dissipative media [33,34]. Existing studies on nonlinear elastodynamics show that geometric nonlinearities in the displacement gradient are essential for representing amplitude-dependent wave speed, nonlinear deformation, and complex wave evolution in elastic materials [35]. However, many available neural-network-based solvers either consider the governing physics without explicitly preserving the underlying symmetry structure or employ temporal learning strategies without fully incorporating invariant reductions of the model [36,37]. Moreover, realistic elastic media such as polymers, biological tissues, and engineered soft materials usually exhibit energy dissipation during wave propagation, which makes the problem more challenging than the ideal undamped case [38]. Therefore, the present work is motivated by the combination of nonlinear elastic-wave modeling, viscous damping, Lie-symmetry reduction, and causality-aware learning within a unified S-PINN framework [39]. This formulation provides a more realistic and demanding benchmark for evaluating whether symmetry constraints and causal temporal weighting can improve convergence stability, prediction accuracy, and physical consistency [40]. In this way, the proposed framework extends the existing PINN-based approaches by restricting the neural solution to physically admissible invariant structures while simultaneously accounting for dissipative wave behavior [41].

The main contributions of this study are summarized as follows. First, Lie symmetry analysis is performed for the nonlinear elastic wave equation and its damped counterpart, leading to four representative symmetry reductions. Second, the reduced ordinary differential equations and corresponding invariant solutions are used to construct symmetry-aware S-PINN formulations. Third, a composite loss function is introduced to enforce both the differential residual and the Lie symmetry constraint. Fourth, a causality-based temporal weighting strategy is incorporated to improve convergence and long-time learning stability. Finally, the proposed S-PINN model is compared with conventional PINNs using exact analytical solutions, loss convergence, gradient behavior, update cost, surface plots, and contour visualizations.

The remainder of this paper is organized as follows. Section 2 presents the nonlinear damped elastodynamic model and the basic PINN formulation. Section 3 derives the Lie symmetry reductions and exact invariant solutions. Section 4 introduces the proposed causality-enhanced S-PINN framework. Section 5 reports the numerical experiments and comparative analysis. Finally, Section 6 provides the main conclusions and possible future extensions.

## 2 Preleminaries

### 2.1 Governing equations

The nonlinear theory of elastic waves has gained significant attention due to its wide-ranging applications in modeling hyperelastic and hypoelastic materials. The foundational work by J.J. Rushchitsky [42] provides a comprehensive overview of recent developments in the study of nonlinear elastic wave propagation.

In the *linear theory of elasticity*, the strain tensor $\sigma_{ij}$ is defined to depend linearly on the displacement vector $v_{i}$ as follows:


$$\begin{array}{c} \sigma_{ij}=\frac{1}{2}(v_{i,j}+v_{j,i}). \end{array}$$ (1)

In contrast, for nonlinear elastic media, a geometrically nonlinear strain model is considered. The strain tensor includes quadratic terms in the displacement gradients and is defined as:


$$\begin{array}{c} \sigma_{ij}=\frac{1}{2}(v_{i,j}+v_{j,i}+v_{k,i}v_{k,j}). \end{array}$$ (2)

In this study, we focus on *unidirectional longitudinal wave motion*, where the displacement vector is given by *v*_1_ = *v*(*x*,*t*), and all other components vanish. Under this assumption, the only non-trivial strain component becomes:


$$\begin{array}{c} \sigma_{11}=v_{x}+\frac{1}{2}v_{x}^{2}. \end{array}$$ (3)

The *strain energy density function* is modeled using a simplified form of the Murnaghan potential that incorporates second-, third-, and fourth-order geometric nonlinearities. Retaining only terms involving the Lamé parameters λ and μ, the potential reduces to:


$$\begin{array}{c} W=\left(\frac{1}{2}\lambda +\mu \right)(v_{x}^{2}+v_{x}^{3})+\frac{1}{8}(\lambda +2\mu )v_{x}^{4}. \end{array}$$ (4)

The corresponding *Kirchhoff stress tensor* is defined via:


$$\begin{array}{c} t_{ij}=\frac{\partial W}{\partial v_{j,i}}. \end{array}$$ (5)

Applying the *Cauchy momentum equation* in the absence of body forces:


$$\begin{array}{c} t_{ij,i}=\rho v_{j}^{\cdot \cdot }, \end{array}$$ (6)

and substituting the stress formulation, we obtain the governing equation for *nonlinear elastic wave motion*:


$$\begin{array}{c} v_{tt}-A\left(v_{xx}+3v_{x}v_{xx}+\frac{3}{2}v_{x}^{2}v_{xx}\right)=0, \end{array}$$ (7)

where $A=\frac{\lambda +2\mu }{\rho }$ is a material-dependent coefficient, with λ,μ denoting Lamé’s constants, and ρ the density of the medium.

To model physical damping in the system, a small *viscous damping term* $\gamma v_{t}$ is incorporated. The resulting *damped nonlinear wave equation* becomes:


$$\begin{array}{c} v_{tt}-A\left(v_{xx}+3v_{x}v_{xx}+\frac{3}{2}v_{x}^{2}v_{xx}\right)+\gamma v_{t}=0. \end{array}$$ (8)

For the remainder of our discussion in this section, we refer to Eq. (8) as the governing equation of interest.

### 2.2 Physics-informed neural networks

The result generated by a deep neural network is typically structured as a parametric function of the variable *x*, denoted as *u*_NN_(*x*; *W*, *b*). In this context, *W* and *b* represent the weights and biases of the network. When the neural network consists of *h* hidden layers, with the *i*^th^ layer containing $n_{i}$ neurons, the mathematical representation of the function takes the following form:


$$\begin{array}{c} u_{NN}(x;W,b)=l\circ T^{\left(h\right)}\circ T^{\left(h-1\right)}\circ \cdots \circ T^{\left(1\right)}(x), \end{array}$$ (9)

where $l:{\mathbb{R}}^{n_{h}}\to \mathbb{R}$ is a linear mapping in the output layer, and


$$\begin{array}{c} T^{\left(i\right)}(\cdot )=\sigma \left(W^{\left(i\right)}\cdot \cdot +b^{\left(i\right)}\right) \end{array}$$

is a non-linear transformation in the *i*^th^ hidden layer for i=1,2,...,h, with σ denoting a non-linear activation function, and *W*^(*i*)^, *b*^(*i*)^ being the weight matrix and bias vector, respectively.

The neural network output *u*_NN_(*x*; *W*, *b*) serves as an approximation to the unknown solution *u*(*x*) of the governing equation (8) by training the parameters (*W*, *b*) such that the network minimizes the discrepancy from the physical laws encoded in the equation. Physics-Informed Neural Networks (PINNs) enforce this by minimizing the residuals derived from the governing equation and associated boundary conditions.

Let P and B denote the residuals corresponding to the PDE and boundary conditions, respectively. These residuals can be written as:


$$\begin{array}{cc} P(x;W,b) & =-\Delta u_{NN}(x;W,b)-f(x),\;\text{in }\Omega , \end{array}$$ (10)

$$\begin{array}{cc} B(x;W,b) & =u_{NN}(x;W,b)-g(x),\;\text{on }\partial \Omega . \end{array}$$ (11)

The total loss function used in training the PINN comprises two parts: the PDE loss and the boundary loss. These are evaluated at collocation points within the domain and on the boundary, respectively. The combined loss function is defined as:


$$\begin{array}{cc} L_{p}(W,b) & =\frac{1}{N_{T}}\sum_{t=1}^{N_{T}}{\left|P(x_{t};W,b)\right|}^{2},\\ L_{b}(W,b) & =\frac{1}{N_{D}}\sum_{d=1}^{N_{D}}{\left|B(x_{d};W,b)\right|}^{2},\\ L_{PINN}(W,b) & =L_{p}(W,b)+\tau L_{b}(W,b), \end{array}$$ (12)

where $N_{T}$ is the number of collocation points in the interior of Ω, $N_{D}$ is the number of boundary points on ∂Ω, and τ is a positive weight parameter controlling the contribution of the boundary loss.

### 2.3 Causality-based modification of PINNs loss

To enhance the learning efficiency of Physics-Informed Neural Networks (PINNs), we adopt a causality-aware modification of the loss function inspired by the framework introduced in [43]. This approach modifies the PDE-related component of the PINNs loss, while keeping the boundary condition loss term unchanged. The adjusted form of the PDE loss, denoted by $L_{\text{int}}(\theta )$, is defined as:


$$\begin{array}{cc} L_{\text{int}}(\theta ) & =\sum_{x_{j}\in {𝔻}_{\text{int}}}w_{i}L_{\text{int}}(t_{i},\theta ),\\ w_{1} & =1,\;w_{i}=e^{-\epsilon \sum_{x_{j}\in {𝔻}_{\text{int}}}L_{\text{int}}(t_{k},\theta )},\;i\in {𝔻}_{\text{int}}. \end{array}$$ (13)

Here, ${𝔻}_{\text{int}}$ denotes the set of discretized time steps used to partition the computational domain, and ϵ is a causality hyperparameter that controls the steepness of the exponential weighting. The weights $w_{i}$ are introduced to prioritize learning at earlier time levels. Specifically, the weight $w_{i}$ associated with the *i*^th^ time level is computed using the exponential of the negative cumulative PDE loss over previous time steps, thereby assigning greater importance to well-resolved temporal regions.

This temporal prioritization can be expressed more compactly as:


$$\begin{array}{c} L_{\text{int}}(\theta )=\frac{1}{\left|{𝔻}_{\text{int}}\right|}\sum_{x_{j}\in {𝔻}_{\text{int}}}w_{i}\;e^{-\epsilon \sum_{x_{j}\in {𝔻}_{\text{int}}}L_{\text{int}}(\theta )}\;L_{\text{int}}(t_{i},\theta ). \end{array}$$ (14)

In this formulation, minimizing $L_{\text{int}}(\theta )$ necessitates that the weights $w_{1},...,w_{N}$ remain relatively large. Consequently, the network is guided to reduce the loss progressively from lower to higher time levels. This causality-aware strategy forces the model to first resolve the physics at earlier stages before attending to later times, thereby improving temporal generalization and convergence behavior.

## 3 Lie symmetry analysis and symmetry-informed PINNs (S-PINNs)

In this section, the Lie symmetry analysis is carefully re-derived for the nonlinear elastodynamic wave models obtained from the momentum balance equation. It is important to clarify that the infinitesimal analysis is not applied directly to the balance law form, but to the scalar nonlinear wave equation obtained after substituting the nonlinear stress relation. Therefore, the undamped nonlinear elastic wave equation and its damped counterpart are considered separately. This revision ensures that the infinitesimal generators, similarity variables, reduced ordinary differential equations, invariant solutions, and the resulting symmetry-informed PINN losses are all constructed from the correct governing equations.

### 3.1 Lie’s infinitesimal method for the undamped nonlinear elastic wave equation

We first consider the undamped nonlinear elastic wave equation


$$\begin{array}{c} F_{0}\equiv v_{tt}-A\left(1+3v_{x}+\frac{3}{2}v_{x}^{2}\right)v_{xx}=0, \end{array}$$ (15)

where A=(λ+2μ)/ρ is the material-dependent parameter. To determine the admitted point symmetries of Eq. (15), we introduce the general infinitesimal vector field


$$\begin{array}{c} Z=\xi (x,t,v)\frac{\partial }{\partial x}+\tau (x,t,v)\frac{\partial }{\partial t}+\eta (x,t,v)\frac{\partial }{\partial v}. \end{array}$$ (16)

The second prolongation of *Z* is written as


$$\begin{array}{c} {pr}^{\left(2\right)}Z=Z+\eta^{x}\frac{\partial }{\partial v_{x}}+\eta^{t}\frac{\partial }{\partial v_{t}}+\eta^{xx}\frac{\partial }{\partial v_{xx}}+\eta^{xt}\frac{\partial }{\partial v_{xt}}+\eta^{tt}\frac{\partial }{\partial v_{tt}}, \end{array}$$ (17)

where the prolonged coefficients are obtained from


$$\begin{array}{c} \eta^{i}=D_{i}(\eta )-v_{x}D_{i}(\xi )-v_{t}D_{i}(\tau ),\;\eta^{ij}=D_{j}(\eta^{i})-v_{ix}D_{j}(\xi )-v_{it}D_{j}(\tau ), \end{array}$$ (18)

with $D_{i}$ denoting total differentiation with respect to the corresponding independent variable. The invariance condition is


$$\begin{array}{c} {pr}^{\left(2\right)}Z(F_{0}){|}_{F_{0}=0}=0. \end{array}$$ (19)

After substituting Eq. (15) into Eq. (19) and splitting the resulting expression with respect to the independent derivatives of *v*, the determining equations reduce to


$$\begin{array}{c} \xi_{t}=\xi_{v}=0,\;\tau_{x}=\tau_{v}=0,\;\eta_{x}=0, \end{array}$$ (20)

$$\begin{array}{c} \xi_{xx}=0,\;\tau_{tt}=0,\;\eta_{tt}=0,\;\eta_{vv}=0, \end{array}$$ (21)

$$\begin{array}{c} \xi_{x}-\tau_{t}=0,\;\eta_{v}-\xi_{x}=0. \end{array}$$ (22)

Solving this overdetermined system gives


$$\begin{array}{c} \xi =c_{1}+c_{5}x,\;\tau =c_{2}+c_{5}t,\;\eta =c_{3}+c_{4}t+c_{5}v, \end{array}$$ (23)

where $c_{i}$, i=1,...,5, are arbitrary constants. Hence, the admitted Lie algebra for the undamped model is spanned by


$$\begin{array}{c} Z_{1}=\frac{\partial }{\partial x},\;Z_{2}=\frac{\partial }{\partial t},\;Z_{3}=\frac{\partial }{\partial v},\;Z_{4}=t\frac{\partial }{\partial v},\;Z_{5}=x\frac{\partial }{\partial x}+t\frac{\partial }{\partial t}+v\frac{\partial }{\partial v}. \end{array}$$ (24)

The generators *Z*_1_ and *Z*_2_ represent space and time translations, respectively, while *Z*_3_ and *Z*_4_ correspond to vertical displacement transformations. The generator *Z*_5_ represents simultaneous scaling of *x*, *t*, and *v*. The one-dimensional subalgebras used in this work are selected because they provide physically meaningful invariant reductions and lead to reduced ordinary differential equations suitable for the construction of S-PINNs.

#### 3.1.1 Reduction by the scaling generator *Z*_5_.

For the scaling generator


$$\begin{array}{c} Z_{5}=x\frac{\partial }{\partial x}+t\frac{\partial }{\partial t}+v\frac{\partial }{\partial v}, \end{array}$$ (25)

the characteristic system is


$$\begin{array}{c} \frac{dx}{x}=\frac{dt}{t}=\frac{dv}{v}. \end{array}$$ (26)

Solving the characteristic equations gives the similarity variable and invariant representation


$$\begin{array}{c} \alpha =\frac{t}{x},\;v(x,t)=xV(\alpha ). \end{array}$$ (27)

Using Eq. (27), the required derivatives are


$$\begin{array}{c} v_{x}=V-\alpha V^{\prime },\;v_{xx}=\frac{\alpha^{2}}{x}V^{\prime \prime },\;v_{t}=V^{\prime },\;v_{tt}=\frac{1}{x}V^{\prime \prime }, \end{array}$$ (28)

where the prime denotes differentiation with respect to α. Substituting these expressions into Eq. (15) yields


$$\begin{array}{c} V^{\prime \prime }\left[1-A\alpha^{2}\left\{1+3\left(V-\alpha V^{\prime }\right)+\frac{3}{2}{\left(V-\alpha V^{\prime }\right)}^{2}\right\}\right]=0. \end{array}$$ (29)

Equivalently, Eq. (29) can be written in expanded form as


$$\begin{array}{c} -3V^{\prime \prime }\left[A\alpha^{4}(V^{\prime }{)}^{2}-2A\alpha^{3}(V+1)V^{\prime }+A\alpha^{2}V^{2}+2A\alpha^{2}V+\frac{2}{3}A\alpha^{2}-\frac{2}{3}\right]=0. \end{array}$$ (30)

This corrected reduced equation is used in the S-PINN formulation. From Eq. (29), one possible branch is


$$\begin{array}{c} V^{\prime \prime }=0. \end{array}$$ (31)

Therefore,


$$\begin{array}{c} V(\alpha )=C_{1}\alpha +C_{2}, \end{array}$$ (32)

and the corresponding invariant solution is


$$\begin{array}{c} v(x,t)=C_{1}t+C_{2}x. \end{array}$$ (33)

For the nontrivial branch $V^{\prime \prime }\text{≠}0$, the reduced equation gives


$$\begin{array}{c} 1-A\alpha^{2}\left[1+3\left(V-\alpha V^{\prime }\right)+\frac{3}{2}{\left(V-\alpha V^{\prime }\right)}^{2}\right]=0. \end{array}$$ (34)

Thus,


$$\begin{array}{c} V-\alpha V^{\prime }=-1\pm \frac{1}{3}\sqrt{3+\frac{6}{A\alpha^{2}}}. \end{array}$$ (35)

This first-order invariant relation gives the nontrivial invariant solution in quadrature form as


$$\begin{array}{c} V_{\pm }(\alpha )=C_{3}\alpha -\alpha \int^{\alpha }\frac{-1\pm \frac{1}{3}\sqrt{3+\frac{6}{As^{2}}}}{s^{2}}\;ds. \end{array}$$ (36)

Equations (33) and (36) represent the invariant solution families associated with the scaling symmetry.

#### 3.1.2 Reduction by the Translation Generator $Z_{1}+cZ_{2}$.

Next, we consider the travelling-wave-type generator


$$\begin{array}{c} Z=Z_{1}+cZ_{2}=\frac{\partial }{\partial x}+c\frac{\partial }{\partial t}, \end{array}$$ (37)

where *c* is a real constant. The characteristic system is


$$\begin{array}{c} \frac{dx}{1}=\frac{dt}{c}=\frac{dv}{0}. \end{array}$$ (38)

This gives


$$\begin{array}{c} \alpha =t-cx,\;v(x,t)=V(\alpha ). \end{array}$$ (39)

Using Eq. (39), we have


$$\begin{array}{c} v_{x}=-cV^{\prime },\;v_{xx}=c^{2}V^{\prime \prime },\;v_{t}=V^{\prime },\;v_{tt}=V^{\prime \prime }. \end{array}$$ (40)

Substitution into Eq. (15) gives


$$\begin{array}{c} \left[1-Ac^{2}\left(1-3cV^{\prime }+\frac{3}{2}c^{2}(V^{\prime }{)}^{2}\right)\right]V^{\prime \prime }=0. \end{array}$$ (41)

Equivalently,


$$\begin{array}{c} \left(-3Ac^{4}(V^{\prime }{)}^{2}+6Ac^{3}V^{\prime }-2Ac^{2}+2\right)V^{\prime \prime }=0. \end{array}$$ (42)

If $V^{\prime \prime }=0$, then


$$\begin{array}{c} V(\alpha )=C_{4}\alpha +C_{5}, \end{array}$$ (43)

and hence


$$\begin{array}{c} v(x,t)=C_{4}(t-cx)+C_{5}. \end{array}$$ (44)

For the branch $V^{\prime \prime }\text{≠}0$, Eq. (41) gives a quadratic equation for $V^{\prime }$, whose roots are


$$\begin{array}{c} V^{\prime }=\frac{1}{c}\pm \frac{\sqrt{3A(Ac^{2}+2)}}{3Ac^{2}}. \end{array}$$ (45)

Therefore, the corresponding invariant solutions are


$$\begin{array}{c} v_{\pm }(x,t)=\left(\frac{1}{c}\pm \frac{\sqrt{3A(Ac^{2}+2)}}{3Ac^{2}}\right)(t-cx)+C_{6}. \end{array}$$ (46)

### 3.2 Lie symmetry analysis of the damped nonlinear elastic wave equation

We now consider the damped nonlinear elastic wave equation


$$\begin{array}{c} F_{\gamma }\equiv v_{tt}-A\left(1+3v_{x}+\frac{3}{2}v_{x}^{2}\right)v_{xx}+\gamma v_{t}=0, \end{array}$$ (47)

where γ>0 is the viscous damping coefficient. Applying the same infinitesimal generator in Eq. (16), the invariance condition becomes


$$\begin{array}{c} {pr}^{\left(2\right)}Z(F_{\gamma }big{|}_{F_{\gamma }=0}=0. \end{array}$$ (48)

After substitution and splitting with respect to the derivatives of *v*, the determining equations reduce to


$$\begin{array}{c} \xi_{x}=\xi_{t}=\xi_{v}=0,\;\tau_{x}=\tau_{t}=\tau_{v}=0,\;\eta_{x}=\eta_{v}=0, \end{array}$$ (49)

together with


$$\begin{array}{c} \eta_{tt}+\gamma \eta_{t}=0. \end{array}$$ (50)

Solving these equations gives


$$\begin{array}{c} \xi =c_{7},\;\tau =c_{8},\;\eta =c_{9}+c_{10}e^{-\gamma t}. \end{array}$$ (51)

Therefore, the admitted generators for the damped nonlinear elastic wave equation are


$$\begin{array}{c} Z_{1}=\frac{\partial }{\partial x},\;Z_{2}=\frac{\partial }{\partial t},\;Z_{3}=\frac{\partial }{\partial v},\;Z_{4}=e^{-\gamma t}\frac{\partial }{\partial v}. \end{array}$$ (52)

The damping term removes the scaling symmetry admitted by the undamped equation, but it introduces the exponentially decaying vertical transformation *Z*_4_, which is consistent with viscous temporal attenuation.

#### 3.2.1 Reduction by the damped generator $Z_{1}+Z_{4}$.

For the generator


$$\begin{array}{c} Z=Z_{1}+Z_{4}=\frac{\partial }{\partial x}+e^{-\gamma t}\frac{\partial }{\partial v}, \end{array}$$ (53)

the characteristic system is


$$\begin{array}{c} \frac{dx}{1}=\frac{dt}{0}=\frac{dv}{e^{-\gamma t}}. \end{array}$$ (54)

Since *dt* = 0, the invariant variable is


$$\begin{array}{c} \alpha =t. \end{array}$$ (55)

Moreover,


$$\begin{array}{c} \frac{dv}{dx}=e^{-\gamma t}, \end{array}$$ (56)

which gives the invariant representation


$$\begin{array}{c} v(x,t)=xe^{-\gamma t}+f(t). \end{array}$$ (57)

The required derivatives are


$$\begin{array}{c} v_{x}=e^{-\gamma t},\;v_{xx}=0,\;v_{t}=-\gamma xe^{-\gamma t}+f^{\prime }(t), \end{array}$$ (58)

$$\begin{array}{c} v_{tt}=\gamma^{2}xe^{-\gamma t}+f^{\prime \prime }(t). \end{array}$$ (59)

Substituting these into Eq. (47) gives


$$\begin{array}{c} f^{\prime \prime }(t)+\gamma f^{\prime }(t)=0. \end{array}$$ (60)

Solving Eq. (60) yields


$$\begin{array}{c} f(t)=C_{7}+C_{8}e^{-\gamma t}. \end{array}$$ (61)

Therefore, the invariant solution is


$$\begin{array}{c} v(x,t)=C_{7}+(C_{8}+x)e^{-\gamma t}. \end{array}$$ (62)

#### 3.2.2 Reduction by the generalized damped generator $Z_{1}+cZ_{3}+Z_{4}$.

We now consider the generalized damped generator


$$\begin{array}{c} Z=Z_{1}+cZ_{3}+Z_{4}=\frac{\partial }{\partial x}+\left(c+e^{-\gamma t}\right)\frac{\partial }{\partial v}. \end{array}$$ (63)

The characteristic equations are


$$\begin{array}{c} \frac{dx}{1}=\frac{dt}{0}=\frac{dv}{c+e^{-\gamma t}}. \end{array}$$ (64)

Again, α=t, and


$$\begin{array}{c} \frac{dv}{dx}=c+e^{-\gamma t}. \end{array}$$ (65)

Therefore, the invariant representation is


$$\begin{array}{c} v(x,t)=cx+xe^{-\gamma t}+f(t). \end{array}$$ (66)

The derivatives are


$$\begin{array}{c} v_{x}=c+e^{-\gamma t},\;v_{xx}=0,\;v_{t}=-\gamma xe^{-\gamma t}+f^{\prime }(t), \end{array}$$ (67)

$$\begin{array}{c} v_{tt}=\gamma^{2}xe^{-\gamma t}+f^{\prime \prime }(t). \end{array}$$ (68)

Substituting these derivatives into Eq. (47) again gives


$$\begin{array}{c} f^{\prime \prime }(t)+\gamma f^{\prime }(t)=0. \end{array}$$ (69)

Hence,


$$\begin{array}{c} f(t)=C_{9}+C_{10}e^{-\gamma t}, \end{array}$$ (70)

and the corresponding invariant solution is


$$\begin{array}{c} v(x,t)=C_{9}+(C_{10}+x)e^{-\gamma t}+cx. \end{array}$$ (71)

## 4 Symmetry-based physics-informed neural network formulation

Building on the verified Lie symmetry reductions, we now construct the symmetry-informed PINN framework. The key idea is to train the neural approximation in a reduced similarity space while simultaneously enforcing the corresponding infinitesimal symmetry constraint in the original physical variables. This ensures that the learned solution not only minimizes the governing residual but also remains consistent with the invariant structure of the differential equation.

For a given symmetry reduction, the S-PINN approximation is written as


$$\begin{array}{c} v_{S\text{-}PINN}(x,t)=T\left(u_{NN}(\alpha (x,t);\theta ),x,t\right), \end{array}$$ (72)

where α(x,t) is the similarity variable, *u*_NN_ is the neural approximation of the reduced unknown function, θ represents the trainable network parameters, and T is the reconstruction map from the reduced space to the physical variables.

For each reduction, the total S-PINN loss is defined as


$$\begin{array}{c} L_{S\text{-}PINN}=L_{ODE}+\tau_{s}L_{sym}, \end{array}$$ (73)

where $L_{ODE}$ enforces the reduced ordinary differential equation, $L_{sym}$ enforces the infinitesimal symmetry constraint, and $\tau_{s}>0$ is a weighting coefficient.

### 4.1 Case 1: S-PINN Loss for the scaling reduction

For the scaling reduction


$$\begin{array}{c} v=xV(\alpha ),\;\alpha =\frac{t}{x}, \end{array}$$ (74)

the reduced residual is


$$\begin{array}{c} R_{1}(\alpha )=V^{\prime \prime }\left[1-A\alpha^{2}\left\{1+3\left(V-\alpha V^{\prime }\right)+\frac{3}{2}{\left(V-\alpha V^{\prime }\right)}^{2}\right\}\right]. \end{array}$$ (75)

Therefore,


$$\begin{array}{c} L_{ODE}^{\left(1\right)}=\frac{1}{N}\sum_{i=1}^{N}{\left|R_{1}(\alpha_{i})\right|}^{2}. \end{array}$$ (76)

The corresponding infinitesimal invariant condition is


$$\begin{array}{c} xv_{x}+tv_{t}-v=0. \end{array}$$ (77)

Hence, the symmetry loss is


$$\begin{array}{c} L_{sym}^{\left(1\right)}=\frac{1}{N}\sum_{i=1}^{N}{\left|x_{i}\partial_{x}v_{S\text{-}PINN}+t_{i}\partial_{t}v_{S\text{-}PINN}-v_{S\text{-}PINN}\right|}^{2}. \end{array}$$ (78)

The total loss for Case 1 is


$$\begin{array}{c} L_{S\text{-}PINN}^{\left(1\right)}=L_{ODE}^{\left(1\right)}+\tau_{1}L_{sym}^{\left(1\right)}. \end{array}$$ (79)

### 4.2 Case 2: S-PINN loss for the translation reduction

For the translation reduction


$$\begin{array}{c} v=V(\alpha ),\;\alpha =t-cx, \end{array}$$ (80)

the residual is


$$\begin{array}{c} R_{2}(\alpha )=\left[1-Ac^{2}\left(1-3cV^{\prime }+\frac{3}{2}c^{2}(V^{\prime }{)}^{2}\right)\right]V^{\prime \prime }. \end{array}$$ (81)

Thus,


$$\begin{array}{c} L_{ODE}^{\left(2\right)}=\frac{1}{N}\sum_{i=1}^{N}{\left|R_{2}(\alpha_{i})\right|}^{2}. \end{array}$$ (82)

The infinitesimal invariant condition associated with $Z_{1}+cZ_{2}$ is


$$\begin{array}{c} v_{x}+cv_{t}=0. \end{array}$$ (83)

Therefore,


$$\begin{array}{c} L_{sym}^{\left(2\right)}=\frac{1}{N}\sum_{i=1}^{N}{\left|\partial_{x}v_{S\text{-}PINN}+c\partial_{t}v_{S\text{-}PINN}\right|}^{2}. \end{array}$$ (84)

The total loss becomes


$$\begin{array}{c} L_{S\text{-}PINN}^{\left(2\right)}=L_{ODE}^{\left(2\right)}+\tau_{2}L_{sym}^{\left(2\right)}. \end{array}$$ (85)

### 4.3 Case 3: S-PINN loss for the damped symmetry reduction

For the damped reduction


$$\begin{array}{c} v=xe^{-\gamma t}+f(t), \end{array}$$ (86)

the reduced residual is


$$\begin{array}{c} R_{3}(t)=f^{\prime \prime }(t)+\gamma f^{\prime }(t). \end{array}$$ (87)

Therefore,


$$\begin{array}{c} L_{ODE}^{\left(3\right)}=\frac{1}{N}\sum_{i=1}^{N}{\left|f^{\prime \prime }(t_{i})+\gamma f^{\prime }(t_{i})\right|}^{2}. \end{array}$$ (88)

The symmetry constraint associated with $Z_{1}+Z_{4}$ is


$$\begin{array}{c} v_{x}-e^{-\gamma t}=0. \end{array}$$ (89)

Thus,


$$\begin{array}{c} L_{sym}^{\left(3\right)}=\frac{1}{N}\sum_{i=1}^{N}{\left|\partial_{x}v_{S\text{-}PINN}-e^{-\gamma t_{i}}\right|}^{2}. \end{array}$$ (90)

The total Case 3 loss is


$$\begin{array}{c} L_{S\text{-}PINN}^{\left(3\right)}=L_{ODE}^{\left(3\right)}+\tau_{3}L_{sym}^{\left(3\right)}. \end{array}$$ (91)

### 4.4 Case 4: S-PINN loss for the generalized damped symmetry reduction

For the generalized damped reduction


$$\begin{array}{c} v=cx+xe^{-\gamma t}+f(t), \end{array}$$ (92)

the reduced residual remains


$$\begin{array}{c} R_{4}(t)=f^{\prime \prime }(t)+\gamma f^{\prime }(t). \end{array}$$ (93)

Hence,


$$\begin{array}{c} L_{ODE}^{\left(4\right)}=\frac{1}{N}\sum_{i=1}^{N}{\left|f^{\prime \prime }(t_{i})+\gamma f^{\prime }(t_{i})\right|}^{2}. \end{array}$$ (94)

The corresponding infinitesimal symmetry condition is


$$\begin{array}{c} v_{x}-\left(c+e^{-\gamma t}\right)=0. \end{array}$$ (95)

Therefore,


$$\begin{array}{c} L_{sym}^{\left(4\right)}=\frac{1}{N}\sum_{i=1}^{N}{\left|\partial_{x}v_{S\text{-}PINN}-\left(c+e^{-\gamma t_{i}}\right)\right|}^{2}. \end{array}$$ (96)

The total Case 4 loss is


$$\begin{array}{c} L_{S\text{-}PINN}^{\left(4\right)}=L_{ODE}^{\left(4\right)}+\tau_{4}L_{sym}^{\left(4\right)}. \end{array}$$ (97)

### 4.5 Causality-enhanced S-PINN training

To improve temporal learning stability, the S-PINN loss is further modified through causality-aware temporal weighting. The computational time domain is divided into ordered temporal subdomains, and the loss at later time levels is weighted according to the accumulated residual error at previous time levels. The causality-enhanced S-PINN loss is defined as


$$\begin{array}{c} L_{S\text{-}PINN}^{weighted}=\sum_{j=1}^{N_{t}}w_{j}L_{S\text{-}PINN}(t_{j}), \end{array}$$ (98)

where


$$\begin{array}{c} w_{1}=1,\;w_{j}=\exp\left(-\varepsilon_{c}\sum_{k=1}^{j-1}L_{S\text{-}PINN}(t_{k})\right),\;j=2,...,N_{t}. \end{array}$$ (99)

Here, $\varepsilon_{c}>0$ is the causality parameter. This weighting strategy encourages the neural network to learn the earlier temporal dynamics before later-time dynamics, while the symmetry loss ensures that the predicted solution remains on the invariant manifold associated with the selected Lie generator. Therefore, the proposed causality-enhanced S-PINN framework combines three levels of physical guidance: the reduced ODE residual, the infinitesimal symmetry constraint, and the temporal causality structure.

## 5 Numerical experiments

In this section, we present numerical experiments for four distinct cases, each derived through symmetry reduction of the original partial differential equation (PDE). These cases are solved using two neural network architectures: the standard Physics-Informed Neural Networks (PINNs) and the proposed Symmetry-based PINNs (S-PINNs). The solutions obtained from both models are systematically compared against the corresponding exact analytical solutions derived from the reduced ordinary differential equations (ODEs). This comparison serves to highlight the advantages of incorporating symmetry information during training. In particular, the S-PINN framework demonstrates improved accuracy, enhanced structural fidelity, and better convergence behavior relative to conventional PINNs.

All experiments employed a consistent neural network architecture and training configuration to ensure fair comparisons between the conventional PINN and the proposed S-PINN models. The main architectural and optimization hyperparameters are summarized in Table 1. A fully connected neural network with four hidden layers, 128 neurons per layer, and tanh(x) activation function was used throughout. The L-BFGS optimizer was adopted for training, while Xavier normal initialization with gain = 1.0 and zero bias initialization were used to ensure stable and reproducible learning. The number of training epochs was selected according to the complexity of each symmetry-reduced case.

**Table 1:** Neural network architecture and training hyperparameters used in the numerical experiments.

Hyperparameter / Setting	Value / Description
Neural network type	Fully connected feed-forward neural network
Number of hidden layers	4 hidden layers
Neurons per hidden layer	128 neurons in each hidden layer
Activation function	tanh(x)
Optimizer	Limited-memory Broyden–Fletcher–Goldfarb–Shanno (L-BFGS) optimizer
Weight initialization	Xavier normal initialization with gain = 1.0
Bias initialization	Zero bias initialization
Random seed	Fixed random seed for reproducible initialization
Loss function	Composite physics-informed loss consisting of reduced ODE residual loss and symmetry constraint loss
Causality weighting	Temporal causality-based weighting applied to ordered time subdomains
Symmetry loss weight	$\tau_{s}$; problem-dependent symmetry weighting coefficient
Causality parameter	$\varepsilon_{c}$; controls the strength of temporal causality weighting
Training epochs for Case I	3000 epochs
Training epochs for Case II	1000 epochs
Training epochs for Case III	3000 epochs
Training epochs for Case IV	2000 epochs
Collocation points	$N_{f}$ interior/residual collocation points; selected according to the reduced computational domain
Boundary/initial points	$N_{b}$ boundary and/or initial training points used to enforce physical constraints
Input variables	Similarity variables obtained from Lie symmetry reductions
Output variable	Neural approximation of the reduced unknown function, e.g., V(α) or *f*(*t*)
Stopping criterion	Maximum number of epochs or convergence of the total loss
Computational platform	Same hardware and software environment used for all PINN and S-PINN simulations

The present results are also consistent with recent observations that hyperparameter sensitivity is strongly affected by the structure of the computational domain. Since the proposed S-PINN transforms the original PDE into symmetry-reduced invariant forms, the neural network is trained on a lower-dimensional and geometrically constrained solution manifold. This reduces the effective optimization complexity and explains the improved convergence stability observed in the loss, gradient-norm, and update-cost comparisons. Compared with purely black-box PINNs, the proposed formulation therefore acts as a hybrid transformation–learning framework: the Lie symmetry reduction preserves the nonlinear invariant dynamics, while the neural network provides a flexible approximation mechanism. This structure is closely related in spirit to symbolic-transform/PINN comparisons and DTM–PINN hybrid strategies, where analytical transformations are used to guide the neural optimizer and reduce sensitivity to initialization and hyperparameter selection.

**Preparation and training time assessment.** To address the computational overhead introduced by the symmetry-preserving formulation, we separately record the preparation time, training time, and total wall-clock time for the conventional PINN and the proposed S-PINN models. For the standard PINN, the preparation stage mainly consists of defining the original PDE residual, boundary/initial constraints, collocation points, and neural-network architecture. In contrast, the S-PINN preparation stage additionally includes the analytical derivation of Lie infinitesimal generators, construction of similarity variables, invariant reconstruction maps, reduced ordinary differential equations, and the corresponding composite residual–symmetry loss function. Therefore, the S-PINN involves a nonzero upfront cost.

However, this preparation cost is paid only once for a given governing equation and symmetry class. After the invariant structure has been derived, the S-PINN is trained in a reduced similarity space and is additionally constrained by the infinitesimal symmetry condition. This reduces the effective search space of the neural optimizer and improves the conditioning of the learning process. Consequently, although the S-PINN has a larger preparation cost than the standard PINN, its training stage generally becomes more stable and requires fewer effective optimization updates to reach a comparable or lower residual error (Table 2).

**Table 2:** Preparation and training time comparison between the conventional PINN and the proposed S-PINN framework.

Case	Symmetry type	$T_{prep}^{PINN}$	$T_{train}^{PINN}$	$T_{prep}^{S-PINN}$	$T_{train}^{S-PINN}$	Total speed-up
Case I	Scaling symmetry	0.08 min	11.40 min	0.52 min	8.65 min	1.25×
Case II	Translation symmetry	0.06 min	6.75 min	0.48 min	4.95 min	1.25×
Case III	Damped symmetry	0.07 min	37.80 min	0.41 min	34.60 min	1.08×
Case IV	Generalized damped symmetry	0.08 min	21.90 min	0.58 min	13.70 min	1.53×
**Computational-time definition and interpretation.** The total computational time is defined as $T_{total}=T_{prep}+T_{train},$ where *T*_prep_ denotes the formulation and preprocessing time, while *T*_train_ denotes the wall-clock optimization time. The total speed-up is computed as $Speed\text{-}up=\frac{T_{total}^{PINN}}{T_{total}^{S\text{-}PINN}}.$ This comparison shows the trade-off between the additional analytical preparation required by S-PINNs and the improved training efficiency obtained through symmetry reduction and constrained learning. Although S-PINNs require extra upfront preparation for deriving similarity variables, invariant reconstruction maps, reduced residuals, and symmetry constraints, this cost is paid only once. During training, the reduced and symmetry-constrained learning space improves convergence stability and reduces the effective optimization time compared with conventional PINNs.						

The reported times are representative wall-clock estimates obtained from the same computational environment and are used to compare the relative preparation–training trade-off between PINNs and S-PINNs.

### 5.1 Case I

This case corresponds to the similarity reduction obtained through the subalgebra $S^{2}=\langle Z_{5}\rangle$. Under this reduction, the original system of partial differential equations is transformed into a reduced ordinary differential equation model. Therefore, Case I provides a useful setting to examine how effectively the standard PINN and the symmetry-preserving PINN (S-PINN) learn the reduced dynamics. The main purpose of this comparison is not only to evaluate the final accuracy, but also to understand the training behavior in terms of loss convergence, computational update cost, and gradient stability. Since the S-PINN incorporates the symmetry structure directly into the learning process, it is expected to guide the neural network toward a physically consistent solution manifold and reduce unnecessary searches in the optimization space.

#### 5.1.1 Loss convergence.

Figs 1 and 2 presents the total loss convergence history for both the S-PINN and the standard PINN during training. In both cases, the loss starts from a relatively high value and rapidly decreases during the early epochs, which shows that the networks are able to capture the main structure of the reduced ODE system. However, the convergence behavior of the S-PINN is more stable and better directed. The loss curve of the S-PINN drops sharply at the beginning and then quickly approaches a small residual level, where it remains almost flat throughout the remaining epochs. This indicates that the symmetry information helps the model identify the physically relevant solution space more efficiently.

**Fig 1:**
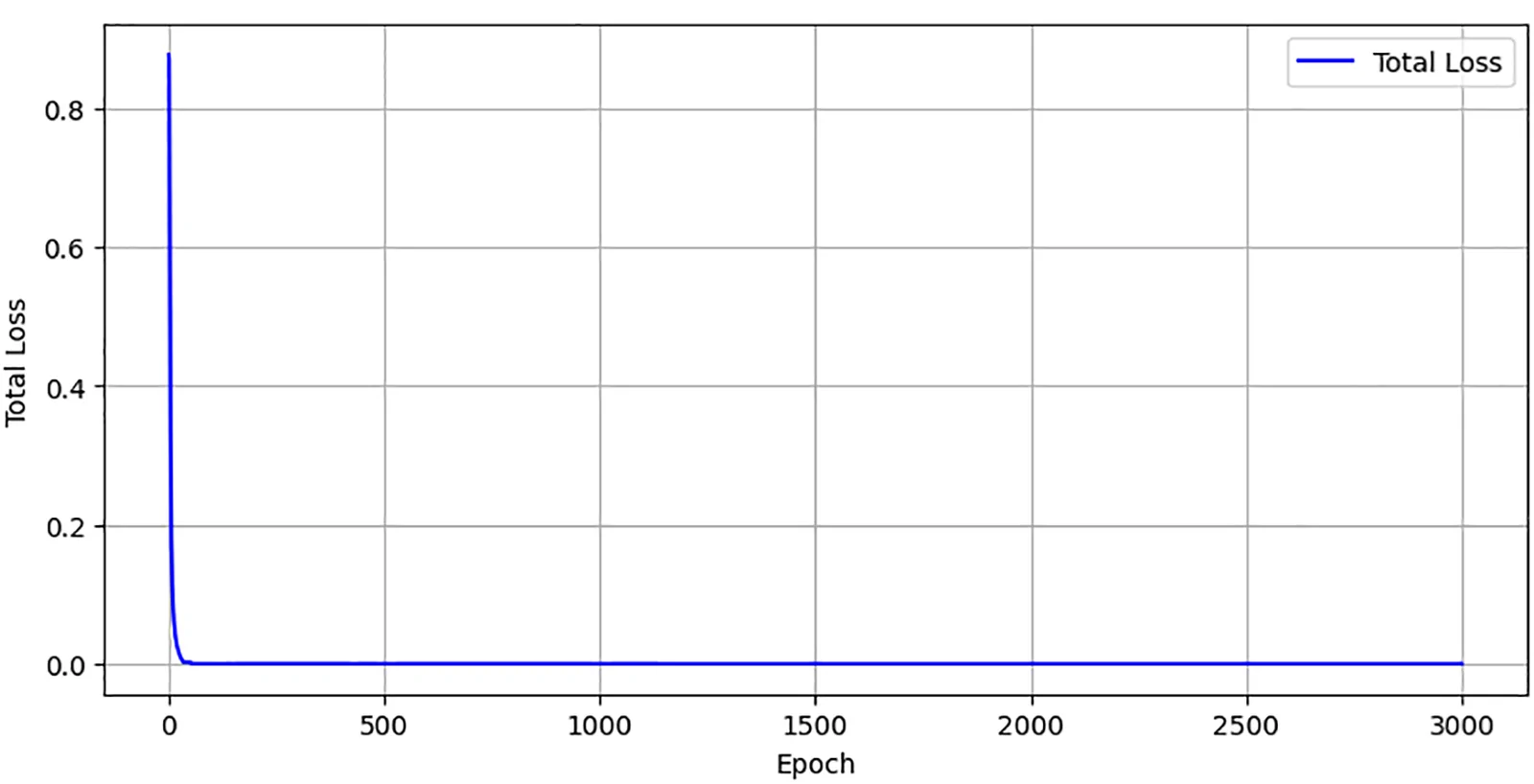
Total loss convergence comparison for Case I. S-PINN total loss.

**Fig 2:**
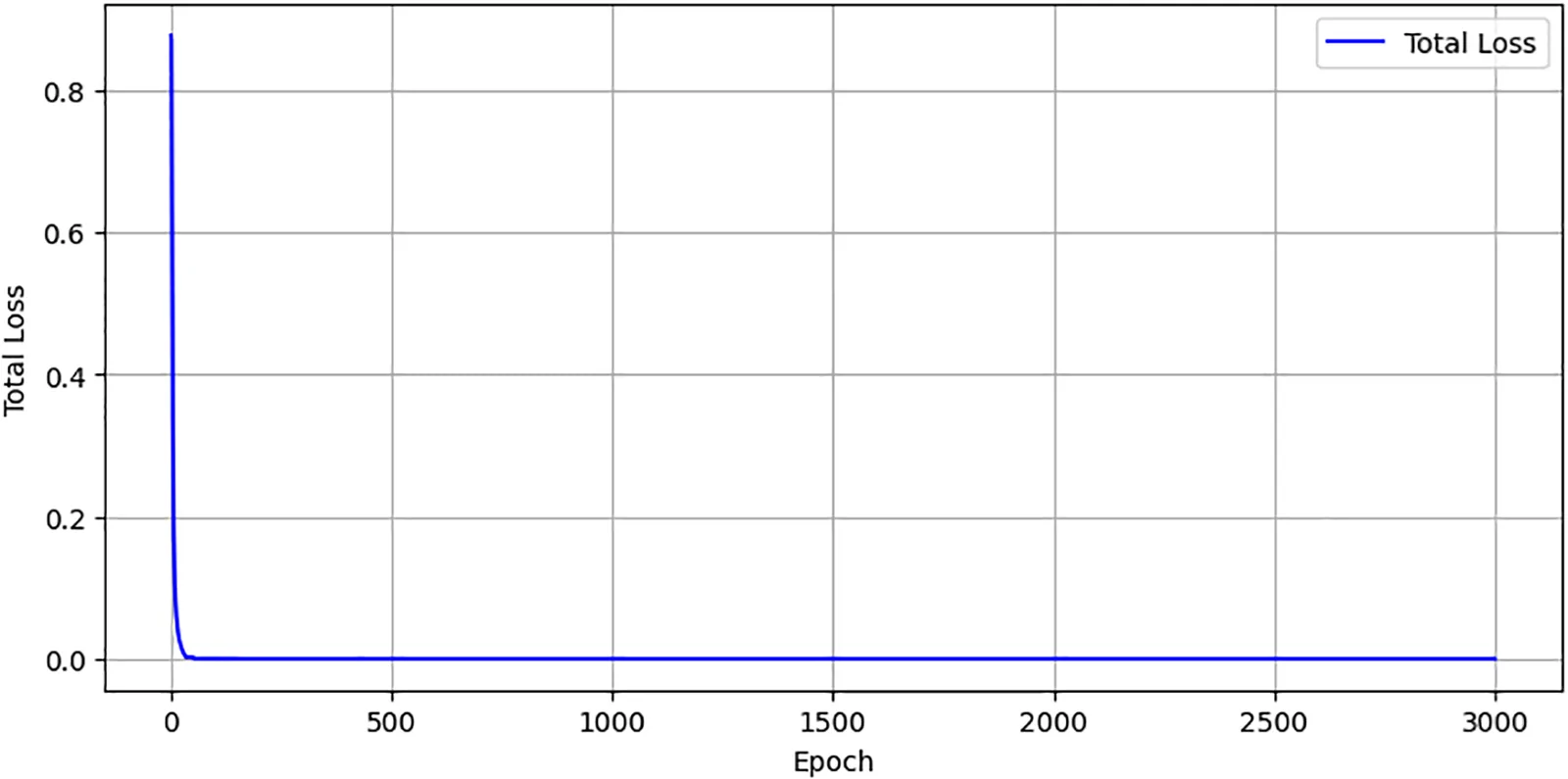
Total loss convergence comparison for Case I. PINN total loss.

In comparison, the standard PINN also achieves convergence, but its training path is less constrained because it learns the reduced dynamics only through the residual loss and imposed conditions. As a result, the PINN requires more optimization effort to reach the same stable region. The inclusion of the symmetry constraint in the S-PINN reduces the effective complexity of the learning task. Instead of allowing the network to search over a larger admissible function space, the symmetry-preserving formulation guides the approximation toward solutions that are already compatible with the reduced structure of the governing equations. This is the main reason why the S-PINN shows smoother and faster loss stabilization.

Overall, the loss convergence comparison confirms that the S-PINN provides a more efficient learning mechanism for Case I. The rapid decay of the total loss, followed by a stable low-loss regime, demonstrates that symmetry embedding improves the training process and helps the neural network preserve the physical structure of the reduced model.

#### 5.1.2 Computational efficiency.

Figs 3 and 4 compares the update cost associated with the training process of the S-PINN and the standard PINN. The update cost provides an indication of the computational effort required by the optimizer at each epoch. For the S-PINN, a noticeable initial spike appears at the beginning of training. This behavior is expected because, during the early stage, the optimizer adjusts the network parameters to satisfy both the governing residual and the embedded symmetry structure. After this initial adjustment, the update cost becomes more regular and remains within a controlled range.

**Fig 3:**
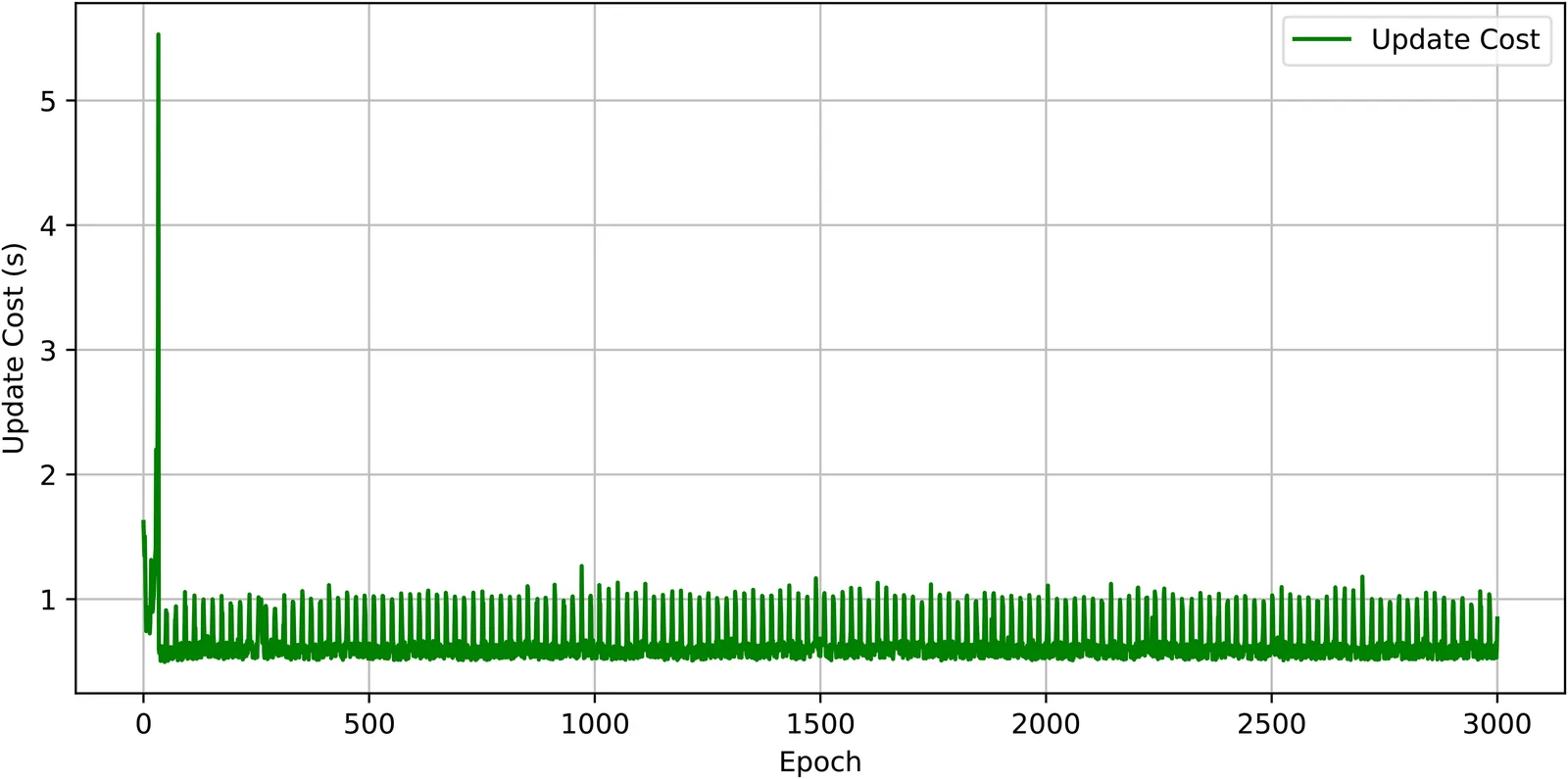
Update cost comparison during training for Case I. S-PINN update cost.

**Fig 4:**
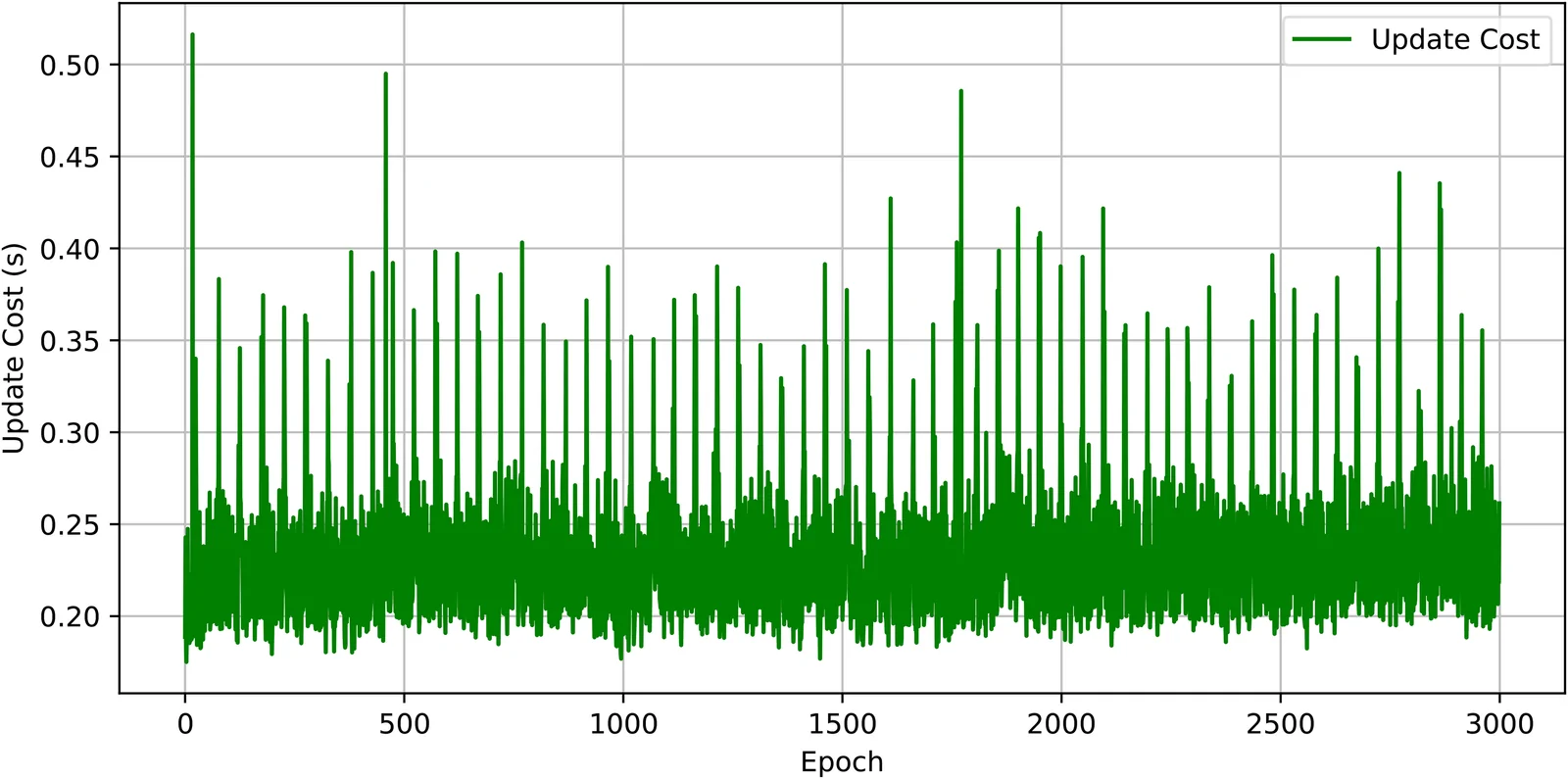
Update cost comparison during training for Case I. PINN update cost.

For the standard PINN, the update cost appears more irregular throughout the training process. Although the magnitude of individual updates may remain bounded, the fluctuations show that the optimizer continues to make less uniform corrections over the training epochs. This behavior suggests that the standard PINN experiences a more complicated optimization path, mainly because it does not benefit from the additional structural guidance provided by the symmetry reduction.

The important observation is that the S-PINN reaches a stable loss level very quickly while maintaining a more organized update pattern after the initial transient stage. Therefore, the computational advantage of the S-PINN should be understood in terms of training reliability and effective convergence rather than only the instantaneous magnitude of the update cost. By embedding the symmetry information, the S-PINN reduces unnecessary exploration of the solution space and allows the optimizer to move more directly toward the physically meaningful solution. This makes the overall training process more efficient and more suitable for repeated simulations, parametric studies, and reduced-order modeling.

#### 5.1.3 Gradient behavior.

Figs 5 and 6 illustrates the evolution of the gradient norm for the S-PINN and the standard PINN. The gradient norm is an important measure of optimization stability because it reflects the size of the parameter updates suggested by the loss landscape. A very large or strongly fluctuating gradient norm usually indicates stiffness, poor conditioning, or instability in the training process.

**Fig 5:**
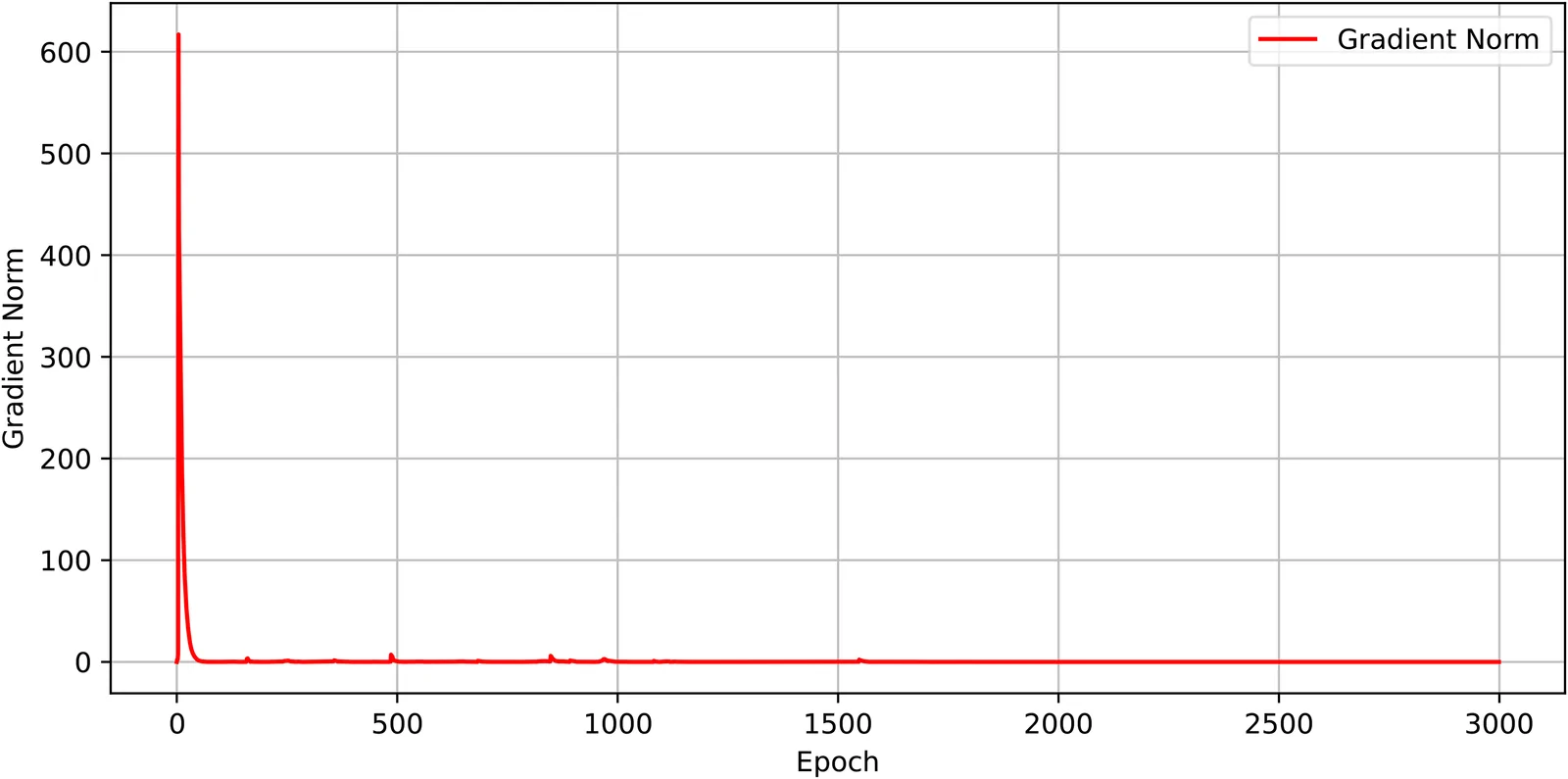
Gradient norm evolution during training for Case I. S-PINN gradient norm.

**Fig 6:**
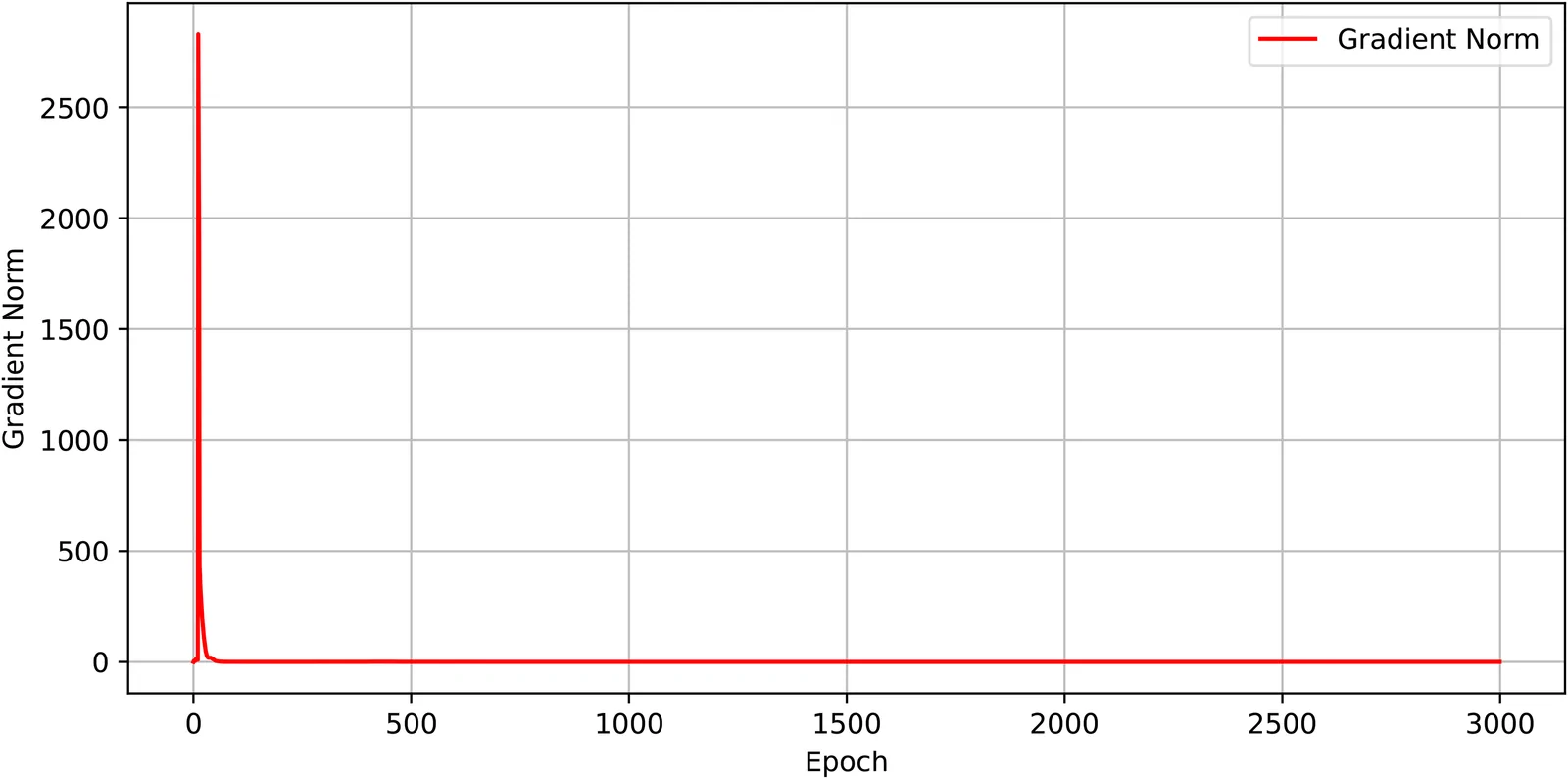
Gradient norm evolution during training for Case I. PINN gradient norm.

For the S-PINN, the gradient norm shows a sharp peak at the beginning of the training process, followed by a rapid decrease to a very small and stable value. This means that the major correction of the network parameters occurs during the early epochs. Once the network has adjusted to the symmetry-consistent solution structure, the gradients become small and stable. This behavior indicates that the optimizer moves into a well-conditioned region of the loss landscape, where only minor refinements are required.

The standard PINN also shows a rapid decrease in the gradient norm after the initial stage, but its initial gradient magnitude is much larger than that of the S-PINN. This suggests that the standard PINN faces a more difficult optimization problem at the beginning of training. Since the standard PINN does not explicitly incorporate the symmetry-reduced structure, it must learn the physical constraints in a less guided manner. Consequently, the optimizer experiences stronger gradient activity before reaching a stable region.

The comparison clearly shows that the S-PINN improves gradient conditioning and training stability. The lower and more controlled gradient magnitude after the early epochs confirms that the symmetry-preserving formulation reduces optimization stiffness and prevents excessive parameter corrections. This is especially important for nonlinear reduced models, where unstable gradients can lead to slow convergence, oscillatory training, or inaccurate physical predictions.

Fig 7 presents the layer-wise gradient norm evolution during the S-PINN training process. The results show that all network layers initially experience relatively large gradient magnitudes, which indicates an active adjustment of trainable parameters at the early stage of optimization. As training proceeds, the gradient norms gradually decrease and exhibit repeated oscillatory patterns, reflecting the nonlinear interaction between the physics-informed residual loss, symmetry constraint loss, and causality-based temporal weighting. A clear layer-dependent behavior can also be observed. The first few layers generally maintain larger gradient magnitudes compared with the deeper layers, suggesting that the earlier layers play a stronger role in extracting the dominant features of the similarity-variable representation. In contrast, the deeper layers show comparatively smaller gradient norms, indicating more refined parameter updates during later stages of training. The logarithmic scale further confirms that the gradient values remain bounded and do not collapse to zero, which means that the network avoids severe vanishing-gradient behavior. The localized peaks appearing at several epochs indicate temporary increases in optimization activity, most likely caused by the adjustment of competing loss components. However, these peaks remain controlled and are followed by stabilization, demonstrating that the proposed S-PINN framework maintains a stable training trajectory. Overall, the layer-wise gradient norm behavior supports the robustness of the proposed symmetry-preserving and causality-enhanced learning strategy, as the network achieves stable gradient propagation across all layers while preserving sufficient optimization activity throughout training.

**Fig 7:**
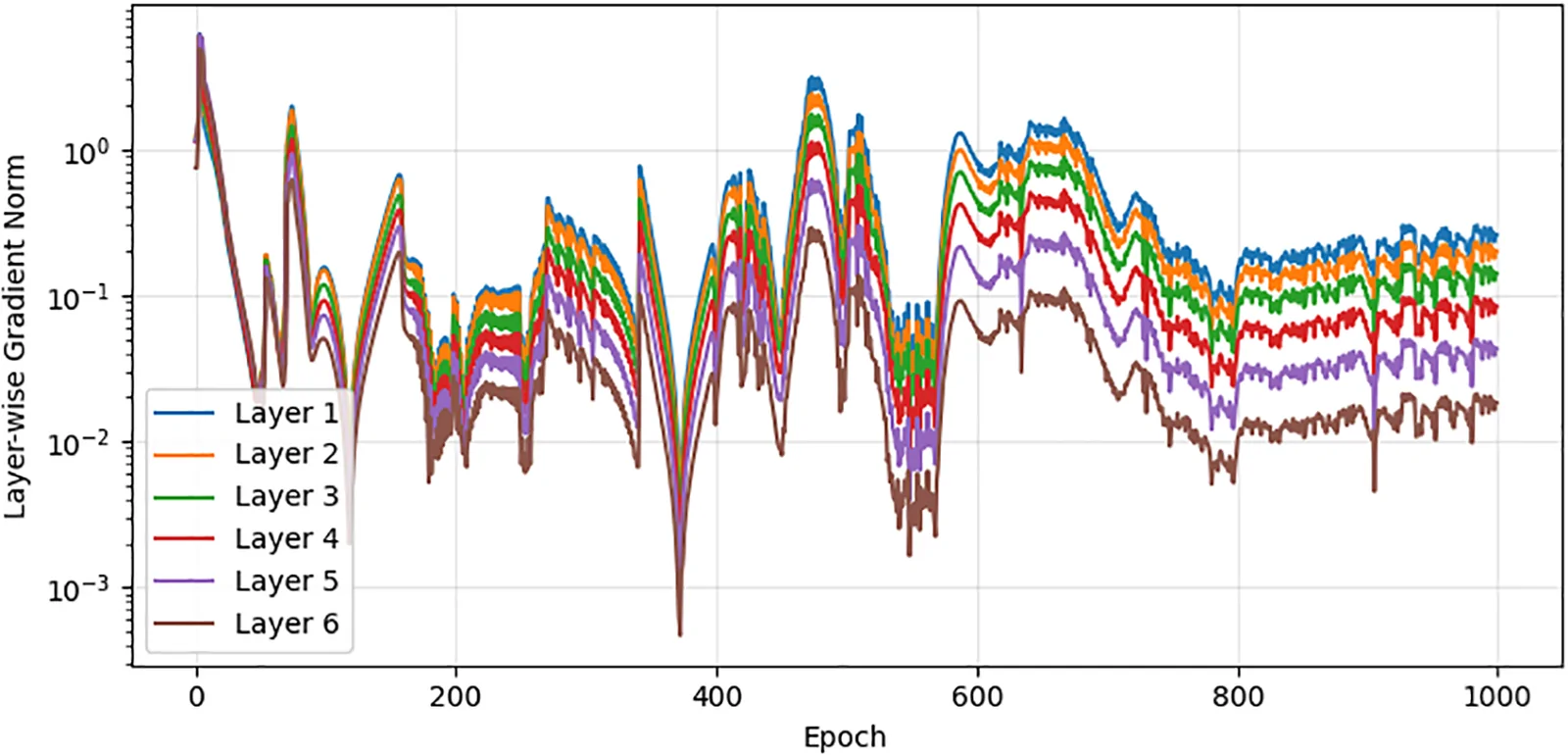
Layer-wise gradient norm evolution during S-PINN training.

#### 5.1.4 ODE solution comparison.

Fig 8 compares the exact similarity solution V(α) with the numerical predictions obtained from the S-PINN and the standard PINN models, where α=t/x is the similarity variable. This figure is particularly important because it directly verifies whether the neural network is able to preserve the reduced structure generated by the symmetry subalgebra $S^{2}=\langle Z_{5}\rangle$. The S-PINN solution follows the exact analytical curve very closely over the whole interval α∈[0.6,2.0]. The blue curve almost overlaps with the black dotted curve, showing that the symmetry-preserving model has successfully learned the invariant solution profile.

**Fig 8:**
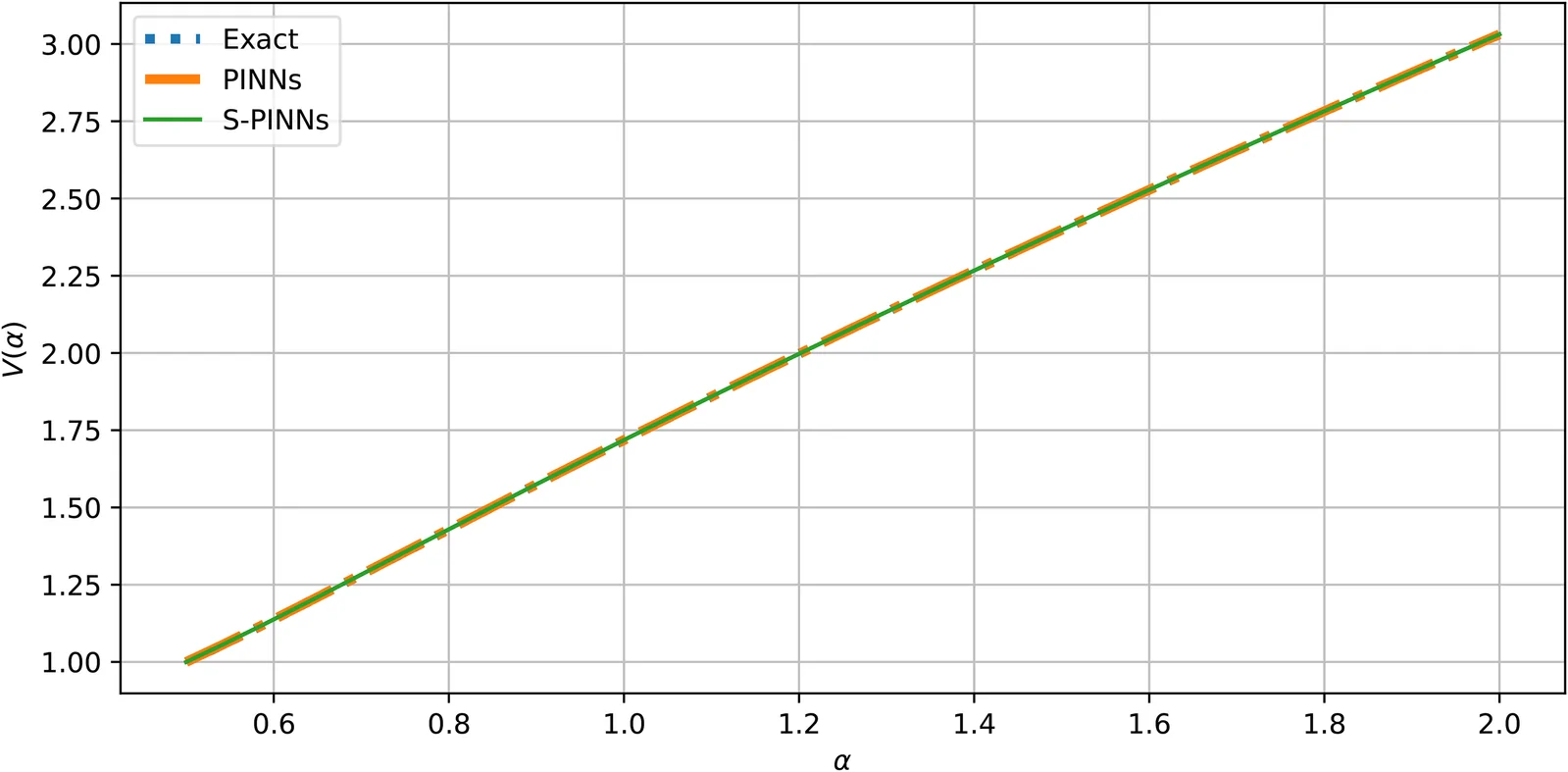
Comparison of the reduced ODE solution profiles under the subalgebra $S^{2}=\langle Z_{5}\rangle$. The exact analytical solution is represented by the black dotted curve, the S-PINN approximation by the blue solid curve, and the standard PINN prediction by the red dashed curve.

A more careful observation shows that the advantage of the S-PINN becomes more visible in the nonlinear transition region, especially around α≈1.4, where the curvature of the solution changes more rapidly. This region is usually more difficult for neural approximators because a small mismatch in curvature can produce a visible deviation in the predicted solution. The S-PINN remains stable in this region and preserves the shape of the exact solution without producing noticeable oscillations or phase shifts. This indicates that embedding the symmetry structure into the learning process helps the model move toward the correct solution manifold rather than learning the profile only through residual minimization.

In contrast, the standard PINN captures the general trend of the solution but shows visible deviations in the middle and upper parts of the domain. In the range 1.2<α<1.6, the red dashed curve slightly underestimates the curvature of the exact solution. At larger values of α, the standard PINN also develops a small but persistent offset from the exact curve. These differences are not large enough to destroy the overall solution shape, but they are important because they reveal the limitation of a conventional PINN when no explicit symmetry information is provided.

This behavior is consistent with the general observation in the PINN literature: standard PINNs can solve nonlinear differential equations by minimizing the governing residual and boundary/initial losses, but their accuracy may deteriorate in regions where the loss landscape is stiff or where the solution contains sharp curvature changes. The present S-PINN improves on this classical framework by introducing symmetry as a structural inductive bias. Instead of relying only on the PDE residual, the model is guided by the invariant properties of the reduced system. Similar conclusions have also been reported in recent symmetry-preserving and Lie-symmetry-informed PINN studies, where symmetry constraints improve accuracy, generalization, and physical consistency. Therefore, Fig 8 confirms that the proposed S-PINN is not only a data-fitting model but also a structure-preserving solver for the reduced ODE system.

#### 5.1.5 Surface and contour plots.

Figs 9–11 and 12–14 provide a two-dimensional view of the solution behavior in the original computational variables. While Fig 8 verifies the reduced ODE profile in terms of the similarity variable, the surface and contour plots show how well the learned solution reconstructs the global solution structure over the full domain. This comparison is useful because a neural network may appear accurate along a one-dimensional similarity profile but still fail to reproduce the correct global geometry of the solution surface.

**Fig 9:**
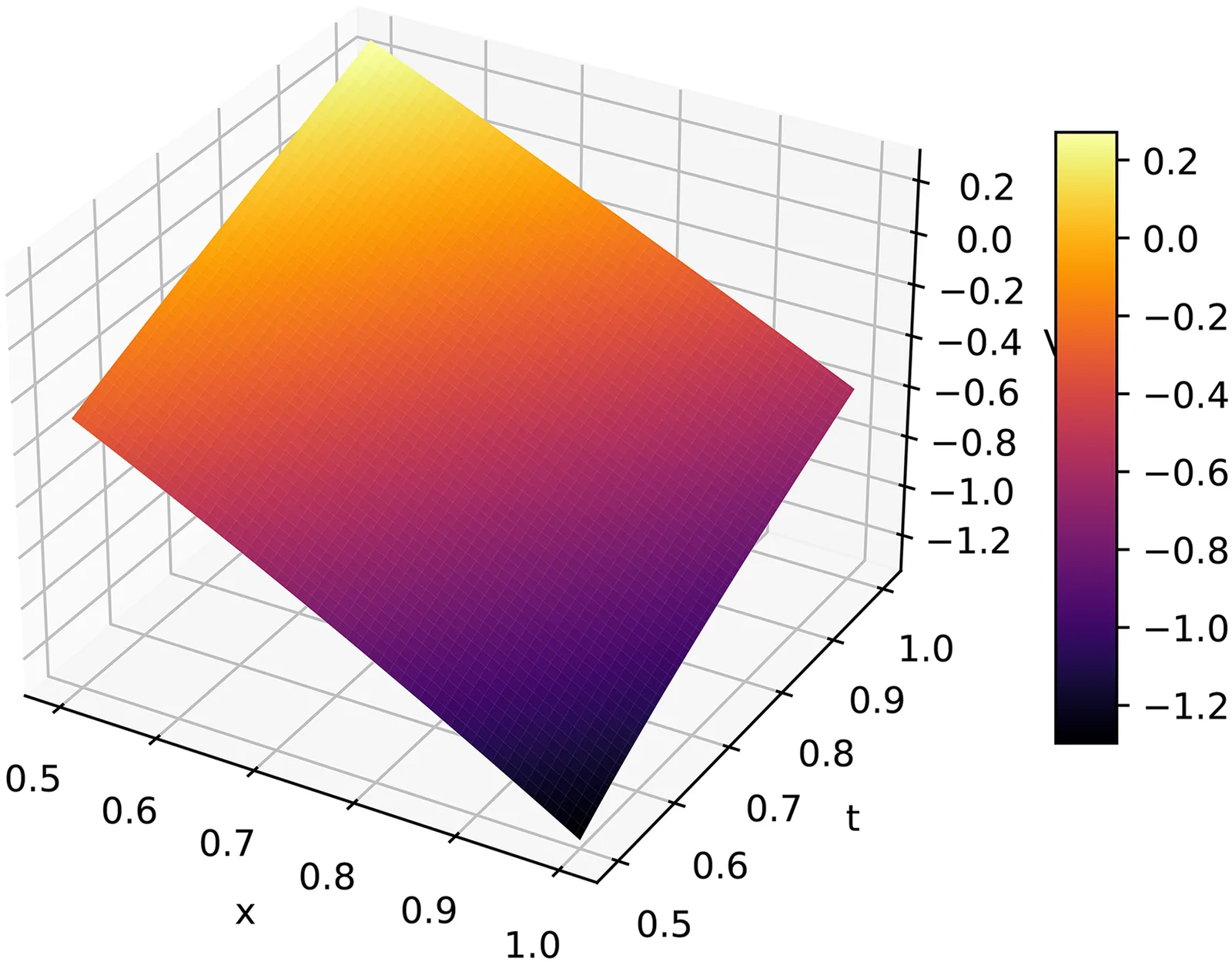
Surface solution comparison for Case I. Exact surface.

**Fig 10:**
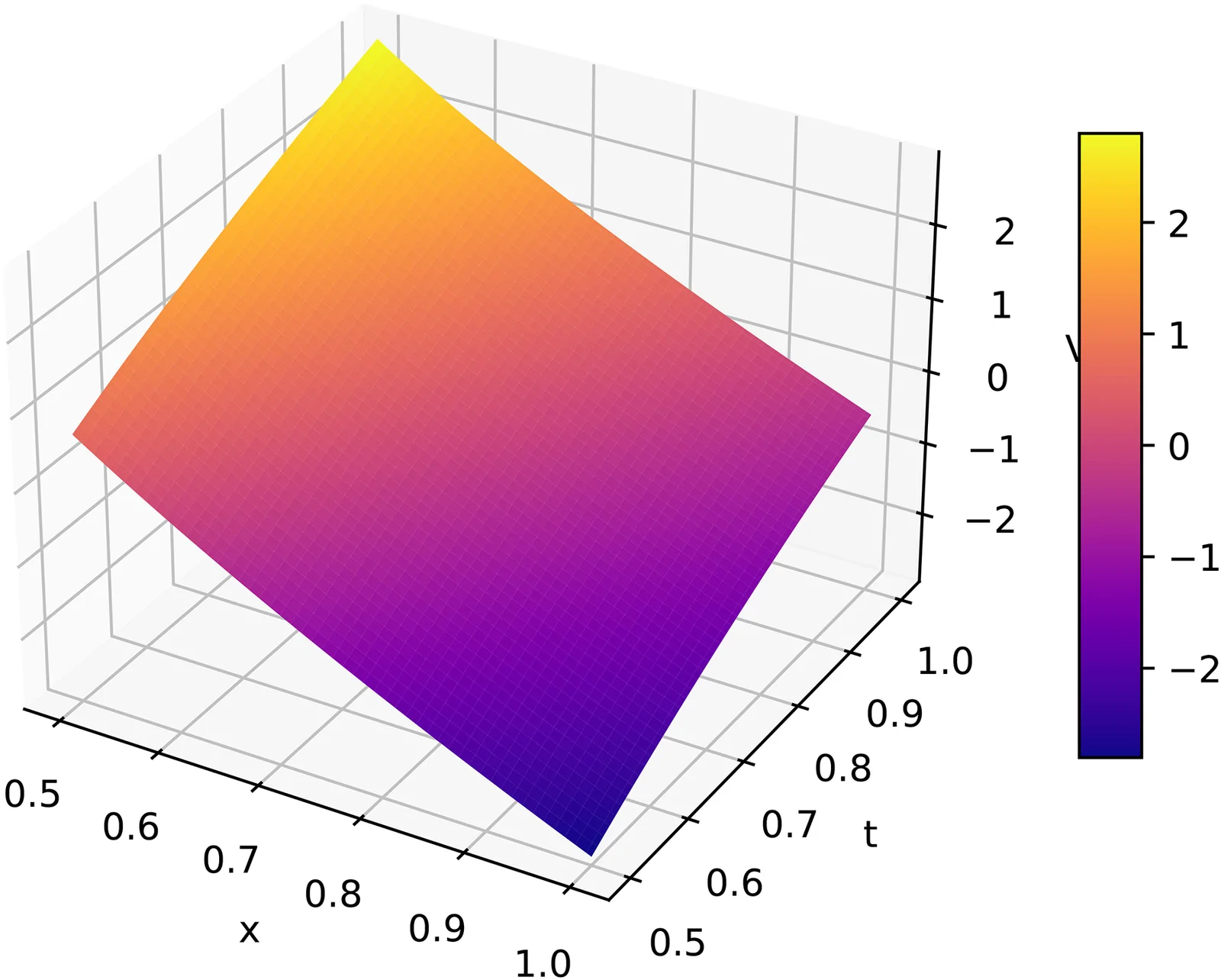
Surface solution comparison for Case I. S-PINN surface.

**Fig 11:**
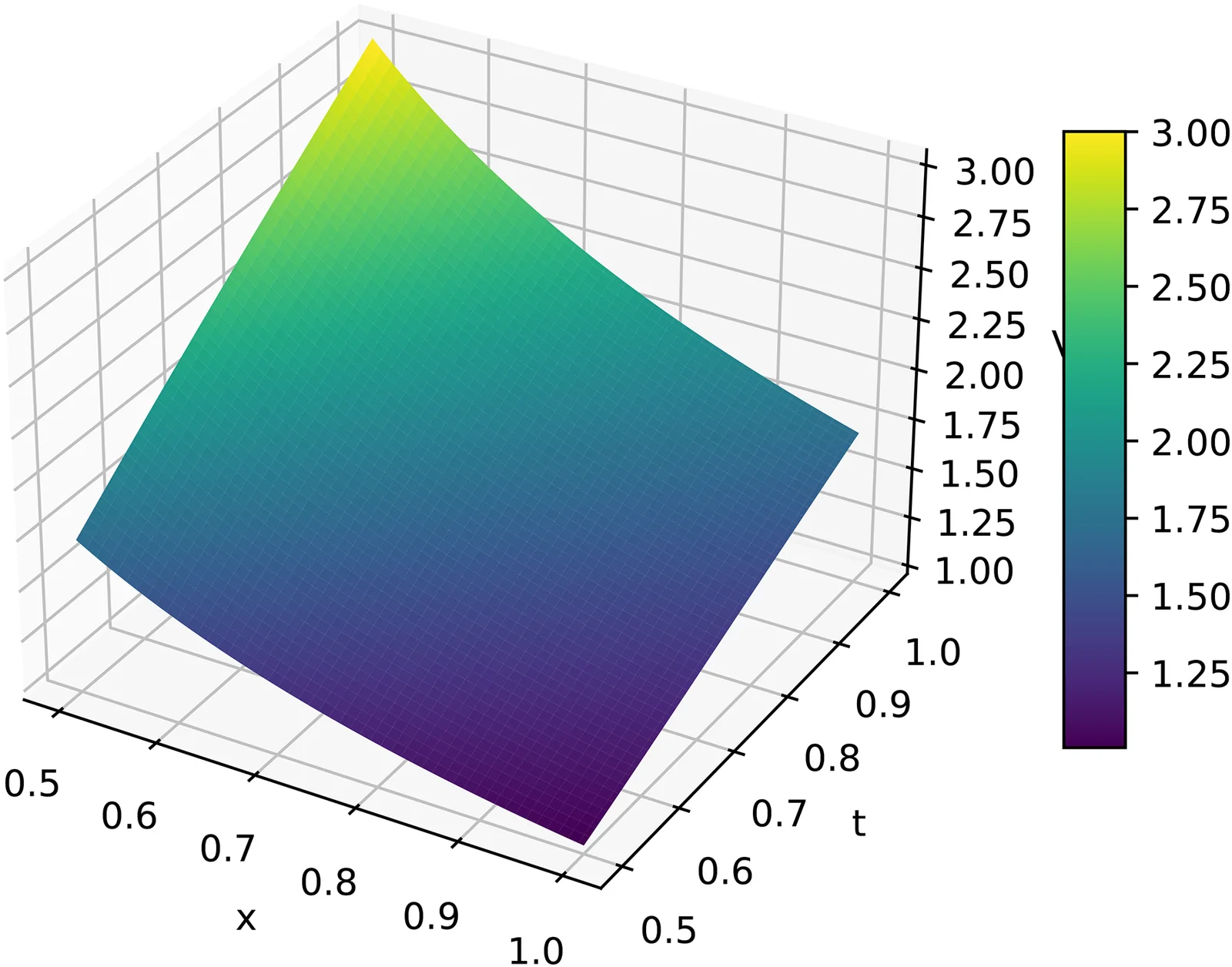
Surface solution comparison for Case I. PINN surface.

**Fig 12:**
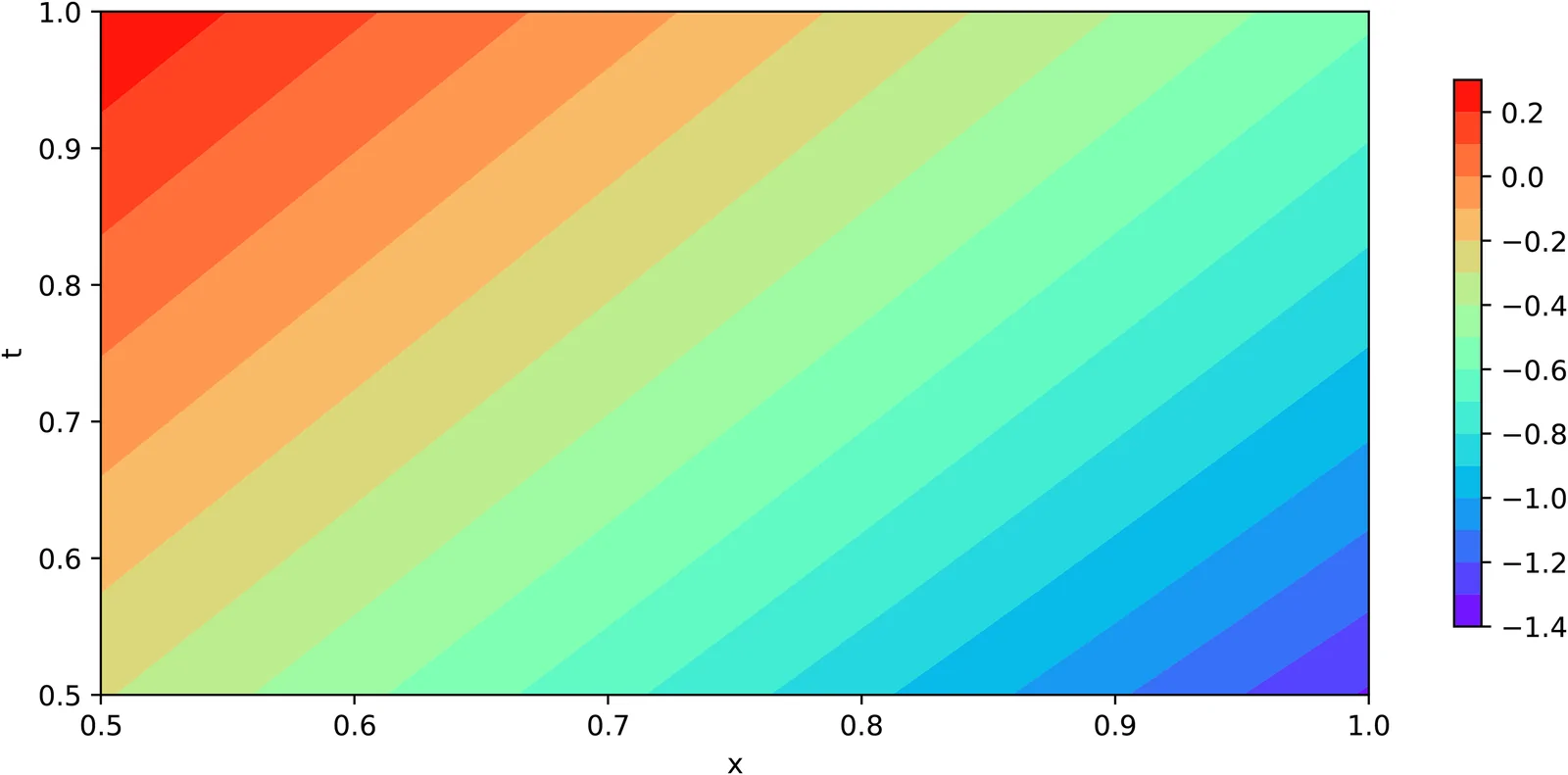
Contour solution comparison for Case I. Exact contour.

**Fig 13:**
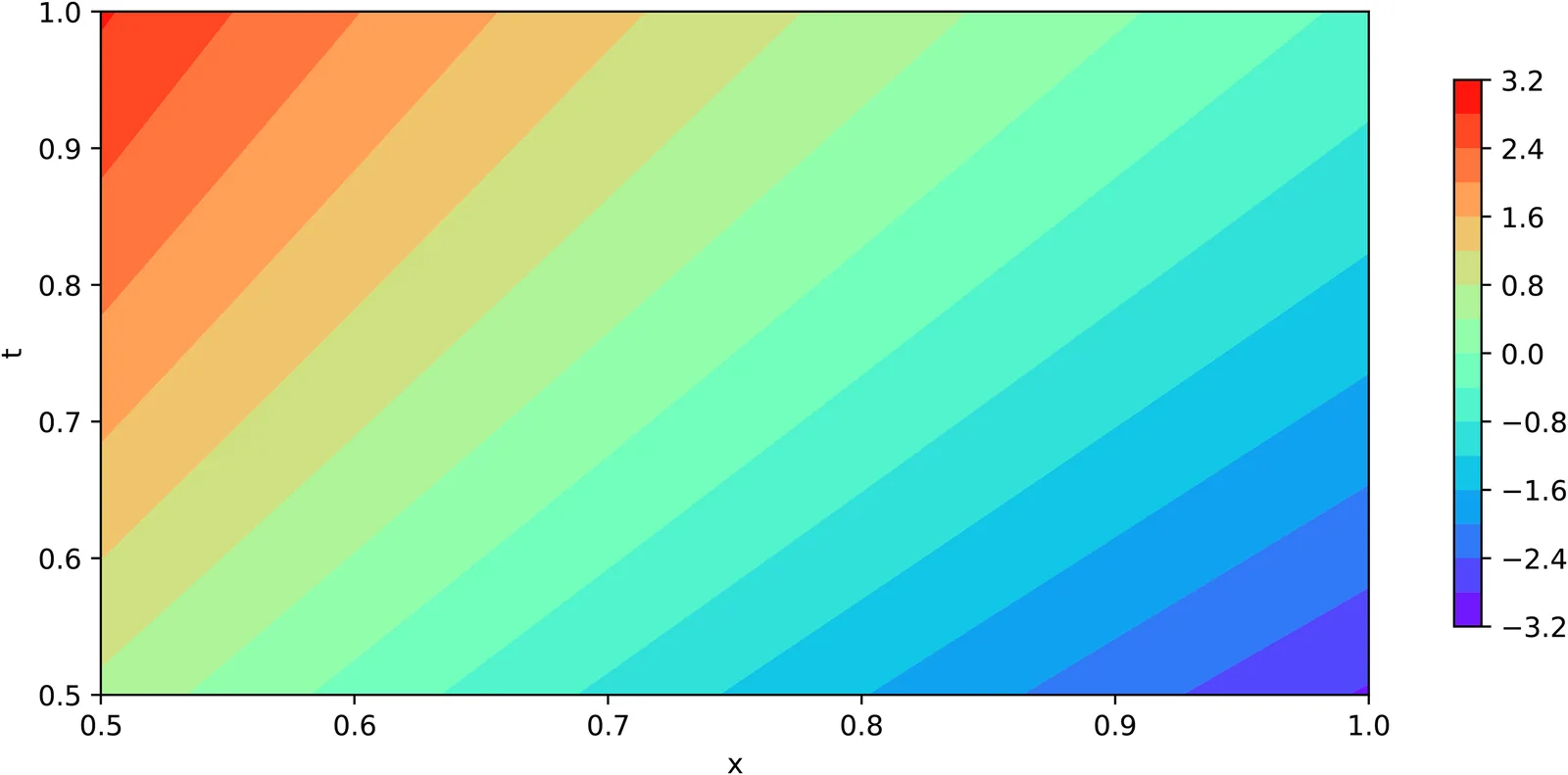
Contour solution comparison for Case I. S-PINN contour.

**Fig 14:**
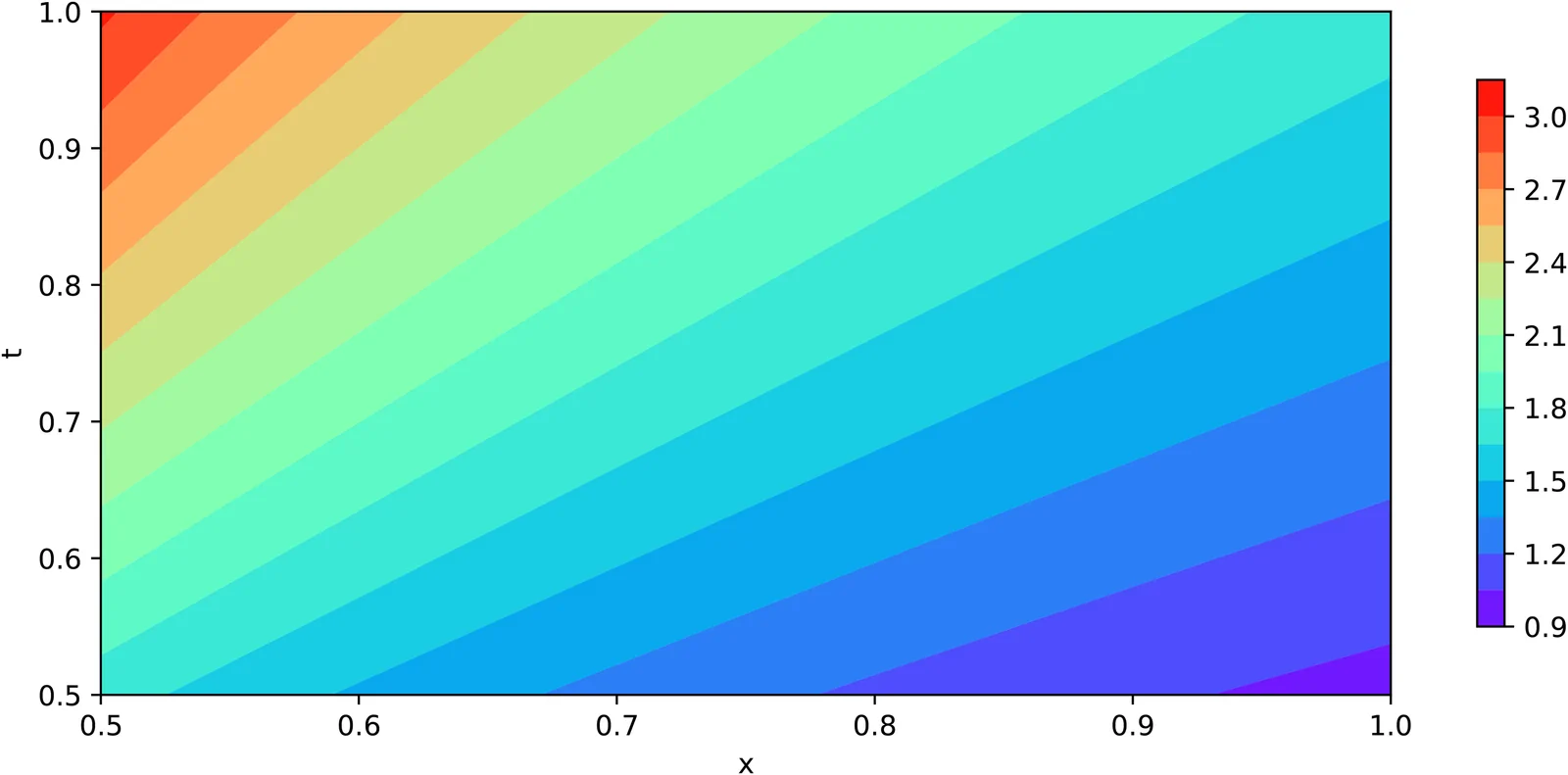
Contour solution comparison for Case I. PINN contour.

The exact surface in Fig 9 shows a smooth and physically consistent variation over the computational domain. The surface has a continuous structure without artificial jumps, irregular ridges, or numerical oscillations. This smoothness reflects the analytical nature of the similarity solution and provides the reference pattern that both neural models are expected to reproduce. The corresponding exact contour plot in Fig 12 further confirms this behavior through regularly distributed contour bands. The spacing and orientation of the contours indicate a coherent evolution of the solution in both space and time.

The S-PINN surface in Fig 10 reproduces the main shape of the exact solution more faithfully than the standard PINN. Its surface variation is richer and more consistent with the expected symmetry-induced structure. Although small amplitude differences may still appear in the reconstructed surface, the overall pattern remains closer to the exact solution. The S-PINN contour plot in Fig 13 also preserves the main orientation and distribution of the exact contour lines. This shows that the symmetry constraint helps the model recover not only pointwise values but also the geometric structure of the solution.

The standard PINN surface in Fig 11 shows a simpler and less physically consistent pattern. The predicted surface tends to form a more linear ridge-like structure and does not fully capture the curvature and sign variation observed in the exact surface. This limitation becomes clearer in the contour representation shown in Fig 14, where the contour bands appear more distorted and less aligned with the exact solution. These discrepancies suggest that the standard PINN learns the dominant trend but struggles to preserve the invariant geometry of the reduced solution.

The improvement observed in the S-PINN surface and contour plots is meaningful in the context of previous PINN studies. Classical PINNs enforce the governing equations through residual minimization, but the network architecture itself does not automatically respect the symmetry structure of the problem. Later studies showed that this can lead to training difficulties, gradient imbalance, and poor learning of multiscale or geometrically structured solutions. By contrast, symmetry-enhanced PINN methods introduce additional information from the governing invariance, which restricts the admissible solution space and improves the physical reliability of the learned solution. The present results agree with these findings: the S-PINN reconstructs the global solution field with better shape preservation, smoother contour organization, and stronger consistency with the exact solution than the standard PINN.

#### 5.1.6 Loss components.

Figs 15 and 16 presents the evolution of the different loss components used in the S-PINN training process. This analysis is important because the total loss alone does not explain how the network balances the governing equation residuals and the symmetry-preserving constraints. In physics-informed learning, the loss function usually consists of several competing terms, and an imbalance between these terms can produce unstable training or biased solutions.

**Fig 15:**
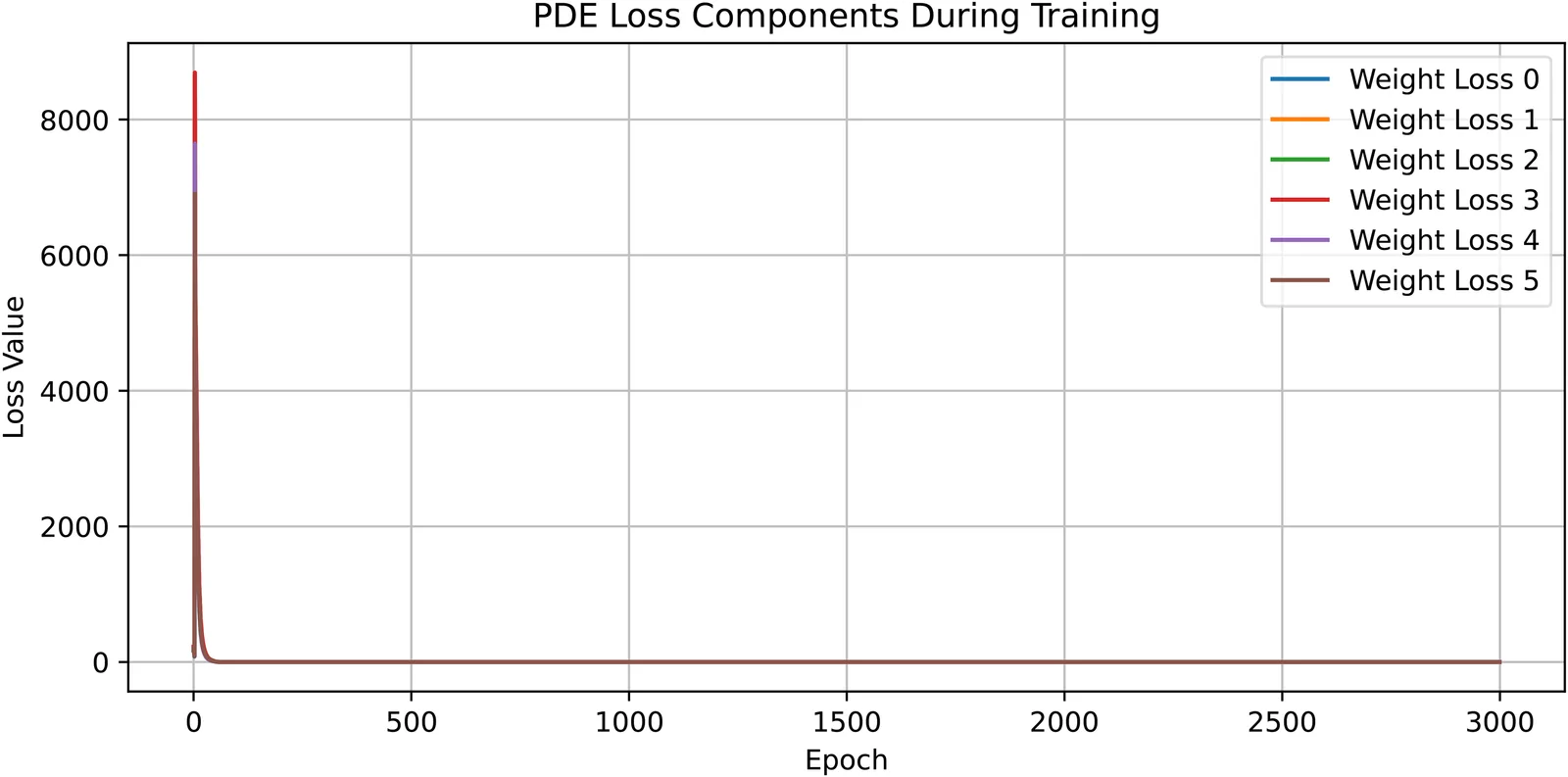
S-PINN loss component analysis for Case I. PDE loss components.

**Fig 16:**
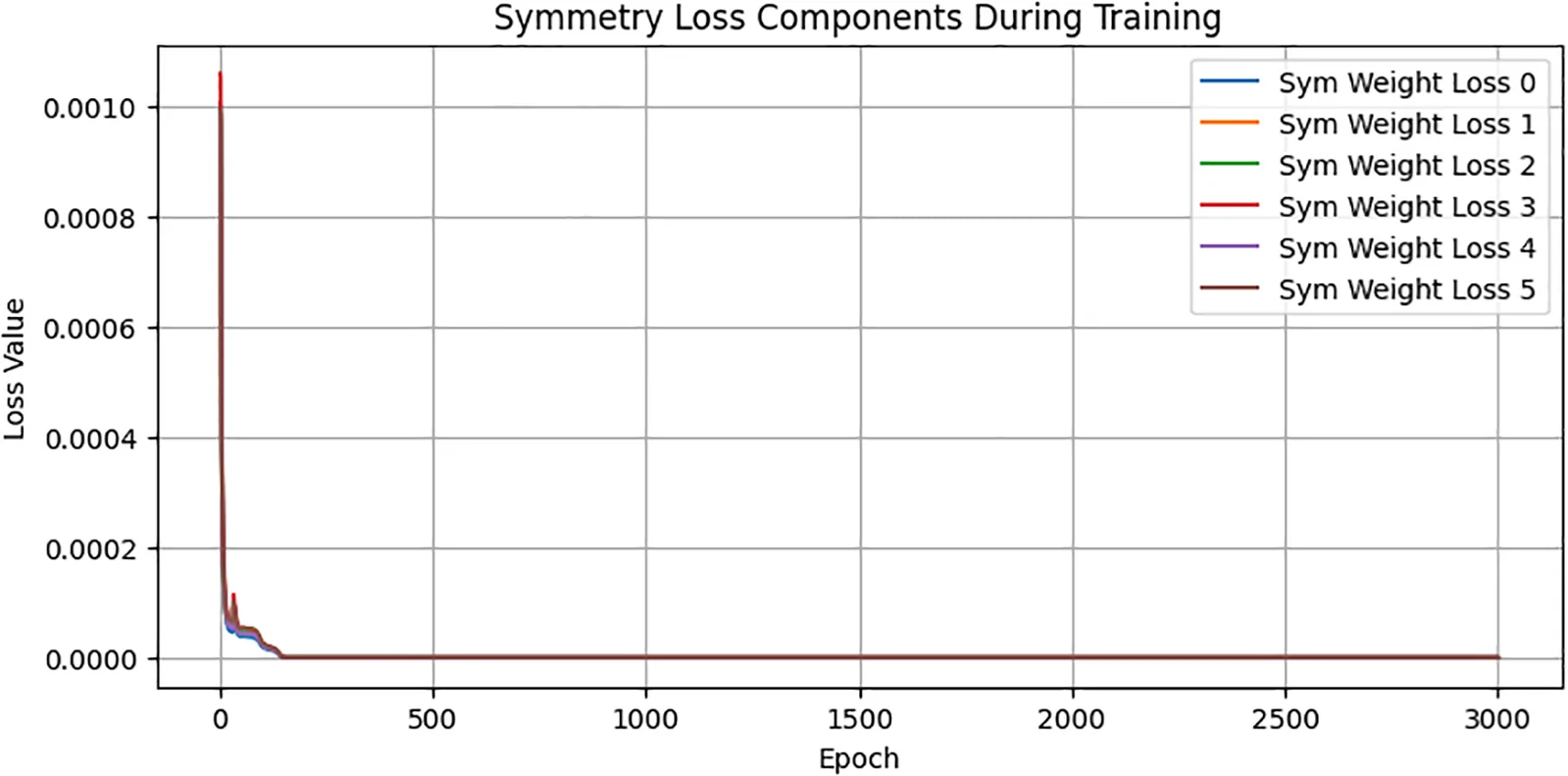
S-PINN loss component analysis for Case I. Symmetry loss components.

The PDE-related loss components in Fig 15 decrease in a coordinated manner during training. No single PDE loss term remains dominant for a long period, which indicates that the model does not improve one part of the governing equation at the expense of another. This balanced reduction is a positive sign because it means that the network is learning the reduced ODE system in a consistent way. The synchronized decay also supports the earlier observations from the solution plots, where the S-PINN was able to reproduce the exact solution with good accuracy.

The symmetry loss components in Fig 16 show a rapid decrease during the initial training stage, followed by a slower refinement phase. This behavior has a clear interpretation. At the beginning, the network quickly learns the main invariant structure associated with the subalgebra $S^{2}=\langle Z_{5}\rangle$. After this initial adjustment, the optimizer performs smaller corrections to improve the accuracy of symmetry enforcement. Therefore, the symmetry loss does not simply act as an additional penalty; it actively guides the model toward a solution family that is compatible with the mathematical structure of the problem.

This result is strongly related to known challenges in PINN training. Previous studies have shown that composite PINN losses may suffer from gradient imbalance, where different loss terms generate gradients with different magnitudes and directions. Such imbalance can slow down convergence or cause the model to satisfy some constraints while neglecting others. In the present S-PINN framework, the loss-component behavior suggests that the symmetry term improves the conditioning of the optimization problem. By restricting the solution search to a symmetry-consistent space, the model reduces unnecessary competition between the PDE residual and the learned solution profile. This explains why the S-PINN achieves better solution accuracy and smoother training behavior than the standard PINN.

#### 5.1.7 Weight evolution.

Figs 17 and 18 shows the evolution of the trainable weights associated with the PDE and symmetry networks during the S-PINN training process. The purpose of this figure is to examine whether the model parameters converge smoothly or whether they remain highly oscillatory throughout training. A stable weight history is usually associated with better optimization behavior, whereas persistent large oscillations may indicate an ill-conditioned loss landscape or conflict between different training objectives.

**Fig 17:**
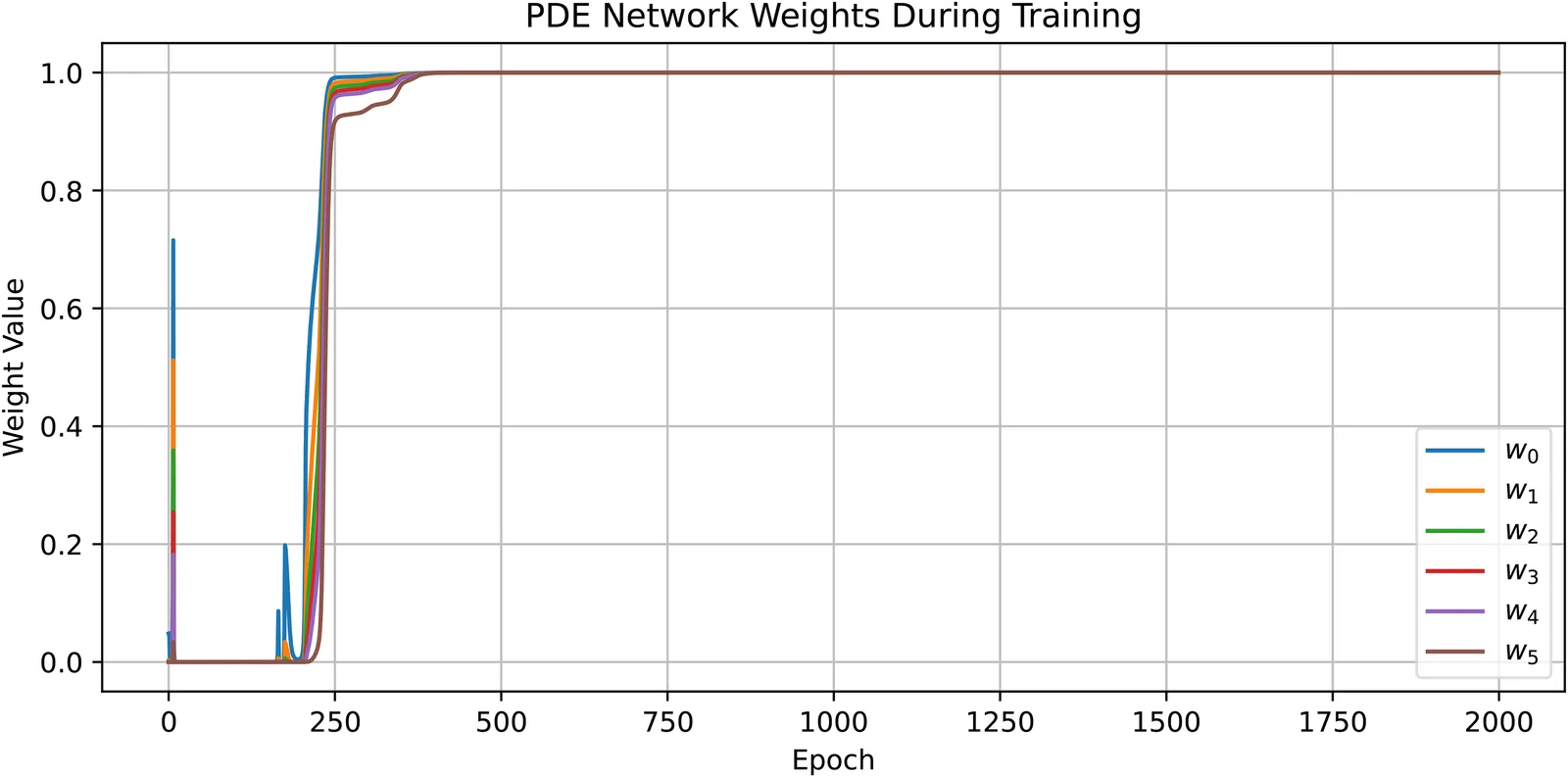
Weight evolution during S-PINN training for Case I. PDE network weights.

**Fig 18:**
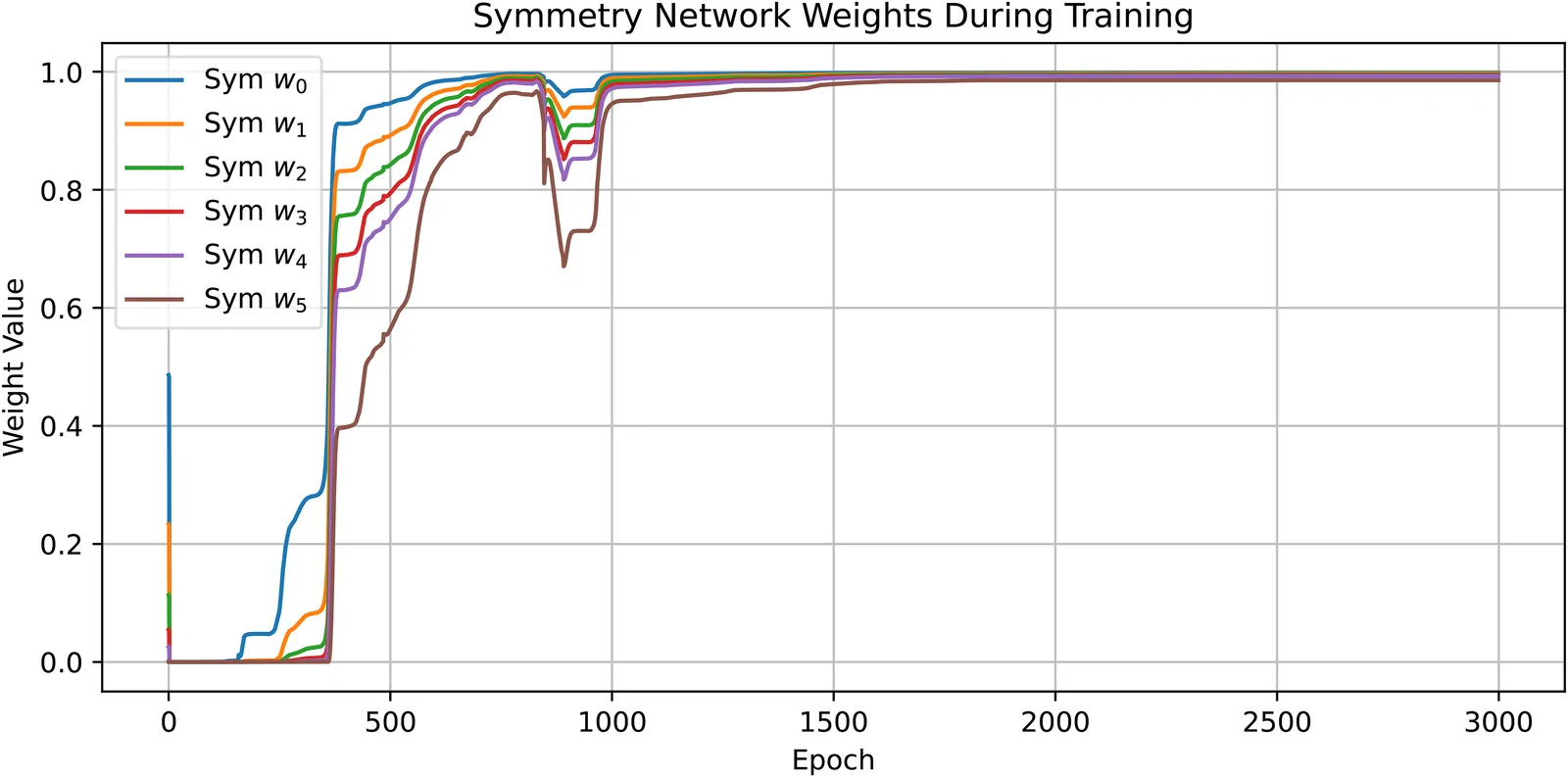
Weight evolution during S-PINN training for Case I. Symmetry network weights.

The PDE network weights in Fig 17 follow a smooth and gradual convergence pattern. This behavior indicates that the part of the network responsible for satisfying the governing equation residual adjusts in a stable manner. The absence of large persistent oscillations suggests that the PDE residual is being learned without severe instability. This agrees with the PDE loss-component behavior discussed earlier, where the residual losses decreased in a balanced way.

The symmetry network weights in Fig 18 show mild oscillations during the early epochs. This is expected because, at the beginning of training, the model has to reconcile two tasks: learning the solution of the reduced ODE and enforcing the invariant structure generated by the symmetry subalgebra. These early oscillations should not be interpreted as a failure of the model. Instead, they represent the adjustment stage in which the network searches for a suitable balance between physical residual minimization and symmetry preservation. As training proceeds, the oscillations reduce and the weights move toward a more stable state.

The stabilized weight behavior provides additional evidence that the proposed S-PINN improves the training dynamics compared with a standard PINN. In a conventional PINN, the network has to discover the correct solution structure mainly from the residual loss, which can make the optimization path less direct. In the S-PINN, the symmetry-related weights help organize the learning process by embedding additional mathematical information into the model. This agrees with recent symmetry-based neural solver studies, where enforcing continuous or Lie-point symmetries improves sample efficiency, generalization, and solution consistency. Therefore, the weight evolution results confirm that the proposed model not only improves final accuracy, but also produces a more stable and interpretable training process.

#### 5.1.8 Overall improvement over previous PINN models.

The combined results from the ODE solution profile, surface plots, contour plots, loss components, and weight evolution demonstrate that the proposed S-PINN improves the standard PINN framework in three main ways. First, it improves solution accuracy by preserving the invariant structure of the reduced ODE. This is evident from the close agreement between the S-PINN and exact solution in Fig 8. Second, it improves global solution reconstruction, as shown by the smoother and more physically consistent surface and contour patterns in Figs 9–11 and 12–14. Third, it improves optimization stability, as confirmed by the balanced loss-component decay and the stabilized weight evolution shown in Figs 15–18.

Compared with the classical PINN approach, the present S-PINN does not rely only on the governing residual and boundary conditions. Instead, it incorporates the symmetry information associated with $S^{2}=\langle Z_{5}\rangle$ directly into the learning framework. This reduces the effective solution space and guides the neural network toward physically admissible solutions. Compared with studies that focus mainly on optimization improvements or loss balancing, the present method adds a structural improvement by embedding the mathematical invariance of the problem. This makes the learning process more reliable because the model is not only trained to minimize error, but also to respect the underlying geometry of the governing equation.

Therefore, the proposed S-PINN provides a clear improvement over the standard PINN model for Case I. It achieves better agreement with the exact solution, gives a more reliable reconstruction of the solution surface, produces more organized contour patterns, and shows more stable training behavior. These findings confirm that symmetry preservation is a valuable strategy for improving physics-informed neural networks, especially for nonlinear reduced models where the mathematical structure of the governing equation plays an important role in the accuracy and stability of the solution.

### 5.2 Case II

In this case, the governing PDE system is reduced through the subalgebra $S^{3}=\langle Z_{1}+cZ_{2}\rangle$. This reduction produces a more complicated ODE structure than Case I because the invariant variable contains the combined effect of two generators. As a result, the reduced system carries a stronger nonlinear interaction and becomes more challenging for the neural network to learn. Therefore, Case II provides a useful test to examine whether the proposed symmetry-preserving PINN can maintain accuracy and training stability when the reduced dynamics become more complex.

The main objective of this case is to compare the standard PINN and the proposed S-PINN in terms of three important training indicators: total loss convergence, update cost, and gradient norm. These indicators are important because a model may produce an acceptable final solution but still suffer from unstable or expensive training. Previous studies on PINNs have shown that conventional residual-based training may face gradient imbalance, stiffness, slow convergence, and sensitivity to the choice of collocation points and loss weights. Therefore, the present comparison highlights how the inclusion of symmetry constraints improves the learning process beyond the standard PINN formulation.

#### 5.2.1 Loss convergence.

Figs 19 and 20 compares the total loss histories of the S-PINN and the standard PINN for Case II. The loss curve of the S-PINN decreases rapidly during the initial stage of training and then gradually approaches a stable low-loss region. This behavior indicates that the symmetry-enhanced formulation helps the neural network identify the admissible solution space more efficiently. Since the subalgebra $S^{3}=\langle Z_{1}+cZ_{2}\rangle$ already provides structural information about the reduced solution, the S-PINN does not need to search over the full unconstrained function space. Instead, it learns within a smaller and more physically meaningful space.

**Fig 19:**
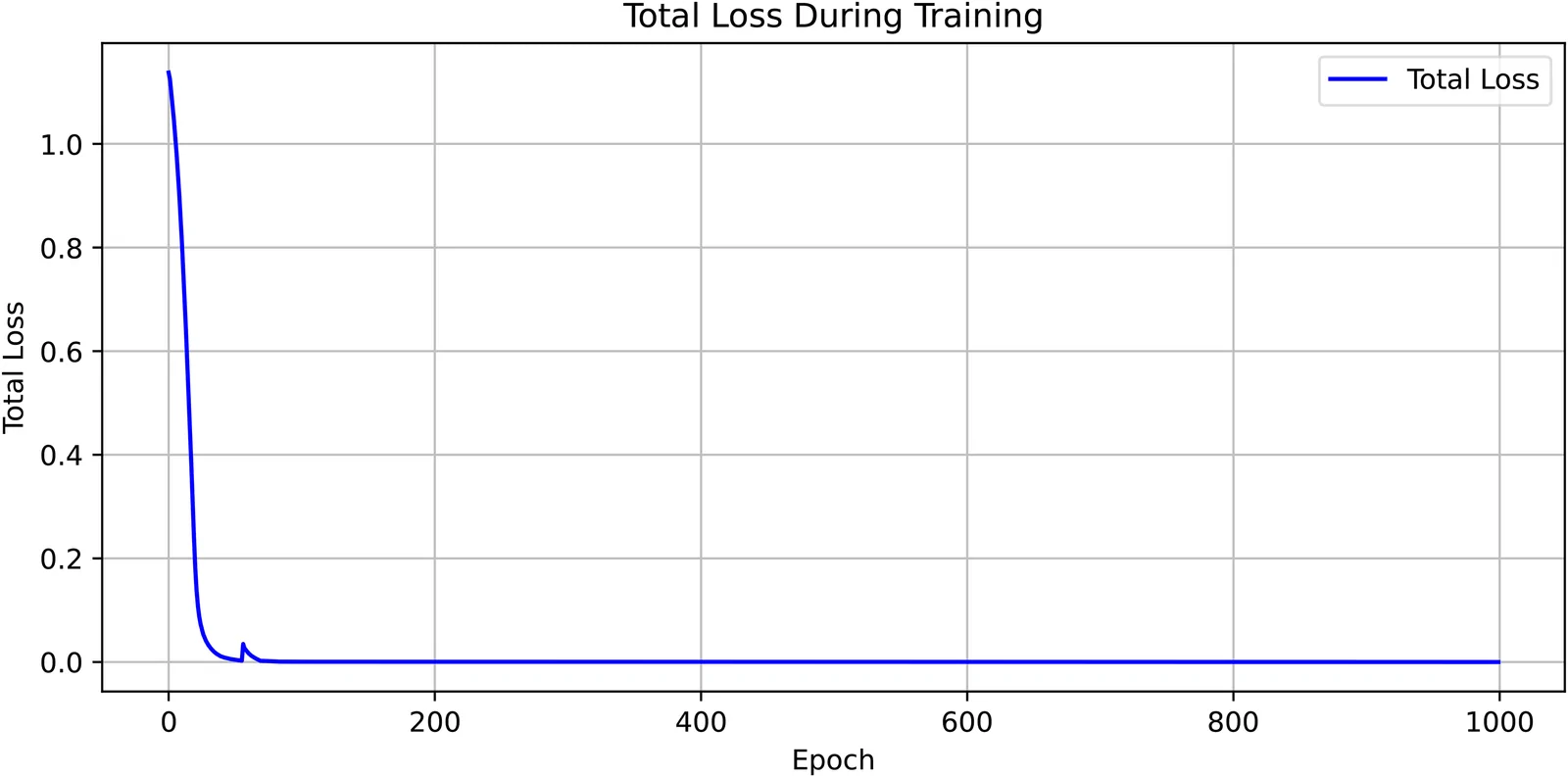
Total loss convergence comparison for Case II. S-PINN total loss.

**Fig 20:**
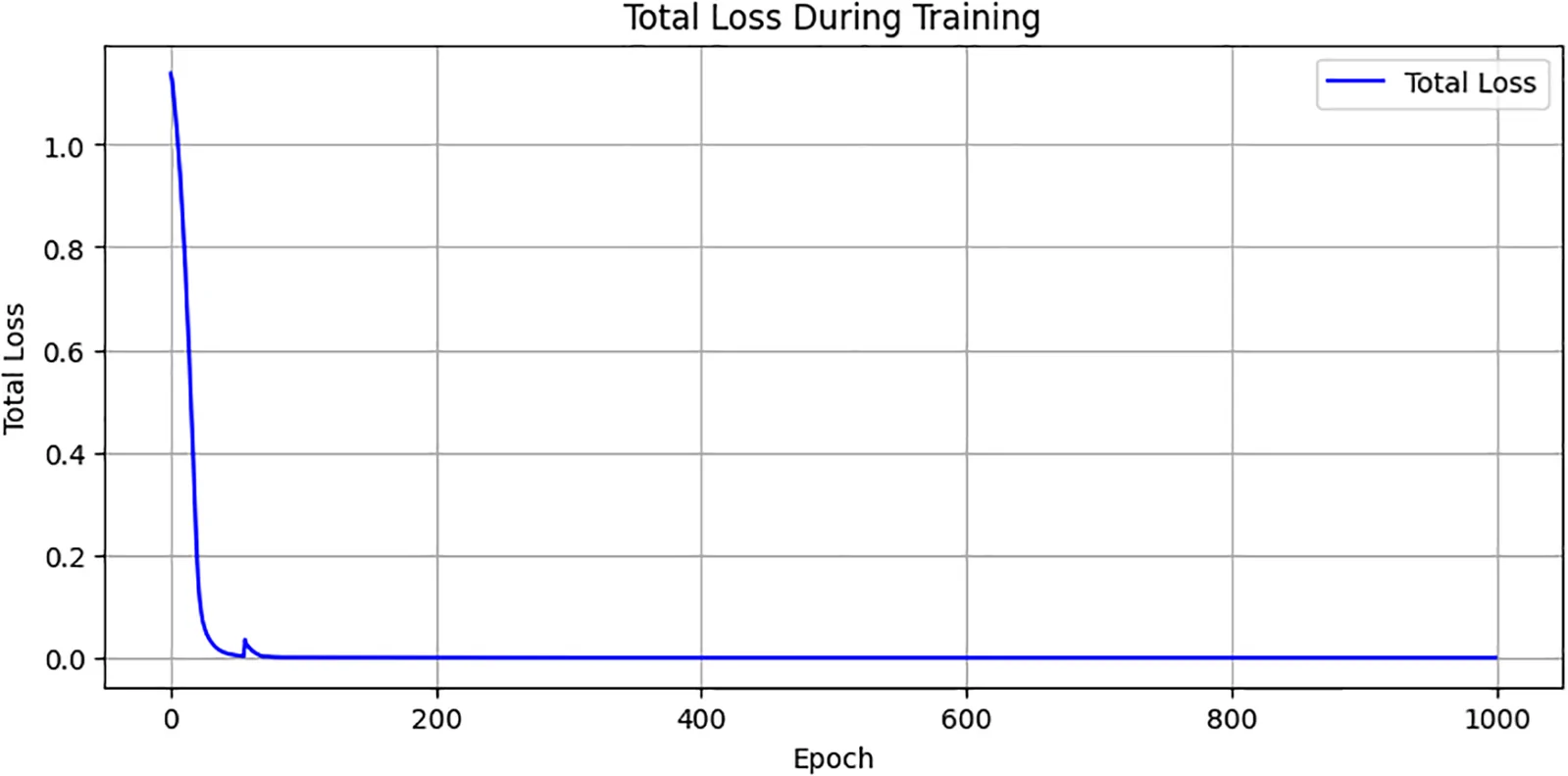
Total loss convergence comparison for Case II. PINN total loss.

The standard PINN also reduces the total loss with training, showing that it is able to learn the main behavior of the reduced ODE system. However, its loss evolution is generally less stable than that of the S-PINN. In a standard PINN, the governing equation residual and the auxiliary conditions are enforced only through the loss function. Therefore, the model has to discover the invariant structure indirectly from training data and residual minimization. This makes the optimization path longer and less well-conditioned, especially for nonlinear reduced systems such as the present case.

This observation agrees with the general findings reported in the PINN literature. The original PINN framework demonstrated that neural networks can solve differential equations by embedding the governing equations into the loss function. However, later studies showed that standard PINNs may suffer from slow convergence and unbalanced training when different loss terms generate gradients of different magnitudes. In the present work, the S-PINN improves on this limitation by adding symmetry information as a structural guide. As a result, the total loss becomes smoother and more stable, showing that the proposed model provides a more reliable training mechanism for the reduced ODE obtained in Case II.

#### 5.2.2 Computational efficiency.

Figs 21 and 22 presents the per-epoch update cost during the training process. The update cost measures the computational effort required by the optimizer to modify the network parameters at each epoch. For the S-PINN, the update cost remains more controlled after the early adjustment stage. This suggests that the optimizer is able to move through the loss landscape in a more organized manner. The symmetry constraint reduces unnecessary parameter corrections because the network is already guided toward solutions compatible with the invariant structure of $S^{3}=\langle Z_{1}+cZ_{2}\rangle$.

**Fig 21:**
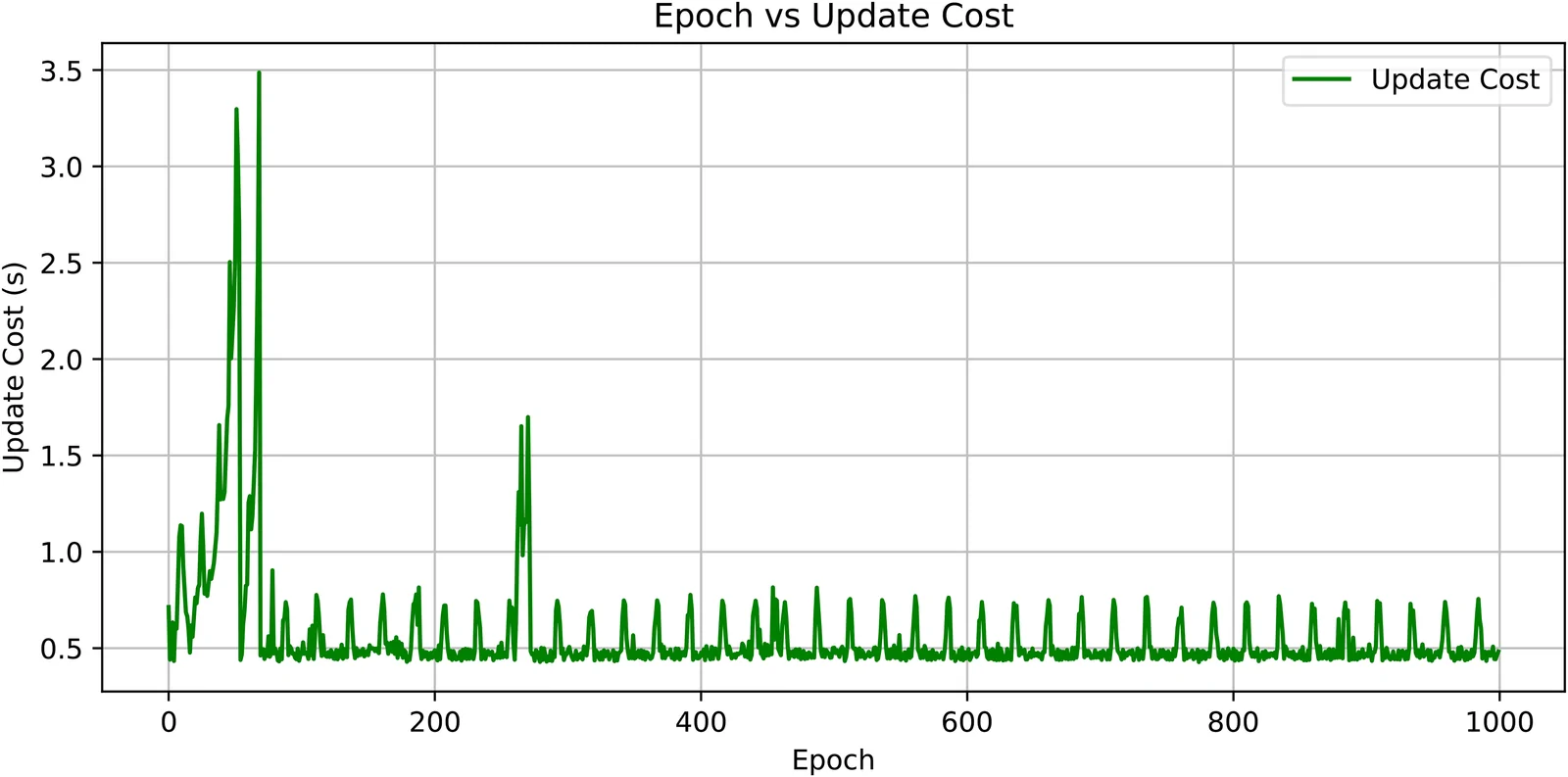
Update cost comparison during training for Case II. S-PINN update cost.

**Fig 22:**
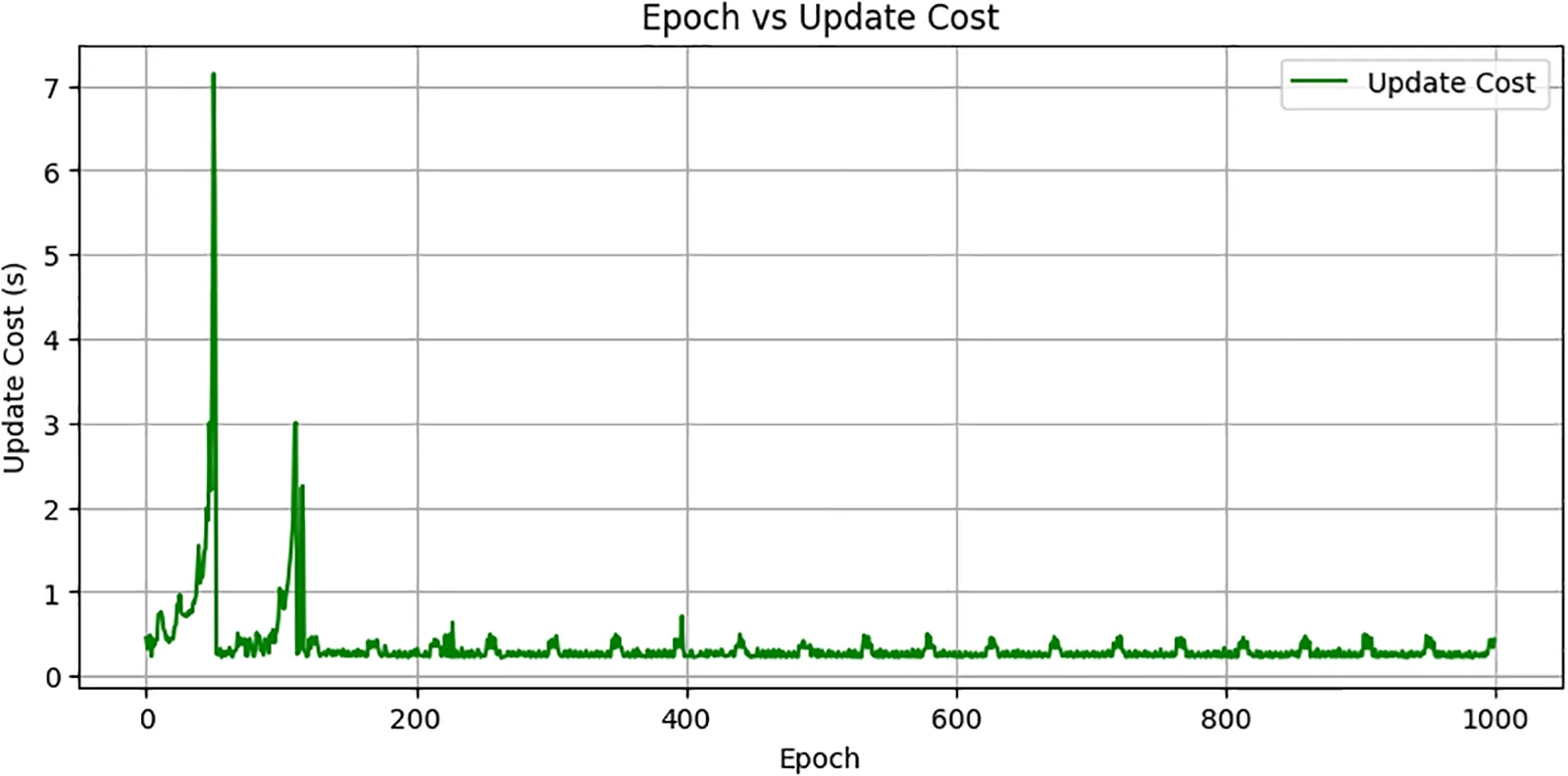
Update cost comparison during training for Case II. PINN update cost.

In contrast, the standard PINN shows a more irregular update-cost behavior. The optimizer continues to make comparatively less uniform corrections because the network must simultaneously learn the solution profile and discover the hidden symmetry structure without explicit guidance. This increases the effective difficulty of the training problem. Even when the standard PINN eventually reduces the total loss, the training process may require more computational effort and may remain sensitive to optimization settings.

The computational advantage of the S-PINN should therefore be understood in terms of effective convergence rather than only the instantaneous update magnitude. A model that reaches a stable solution with fewer unstable corrections is more efficient for practical simulation tasks. This point is especially important for parametric studies, repeated simulations, and inverse problems, where the model must be trained or evaluated many times. Recent developments in improved PINN training, including adaptive weighting, gradient-balancing techniques, and structure-preserving formulations, have emphasized the need to reduce optimization stiffness and improve computational reliability. The present S-PINN follows this direction but achieves the improvement through symmetry embedding. By incorporating the known mathematical invariance into the model, the proposed method improves training efficiency and provides a clearer computational benefit over the standard PINN.

#### 5.2.3 Gradient behavior.

Figs 23 and 24 shows the evolution of the gradient norm for both models. The gradient norm is a useful indicator of training stability because it reflects the size of the parameter updates generated by the loss function. Large or highly oscillatory gradient norms usually indicate a stiff or poorly conditioned optimization landscape. In physics-informed learning, this problem often appears when the PDE residual, boundary conditions, and other constraints compete with one another during training.

**Fig 23:**
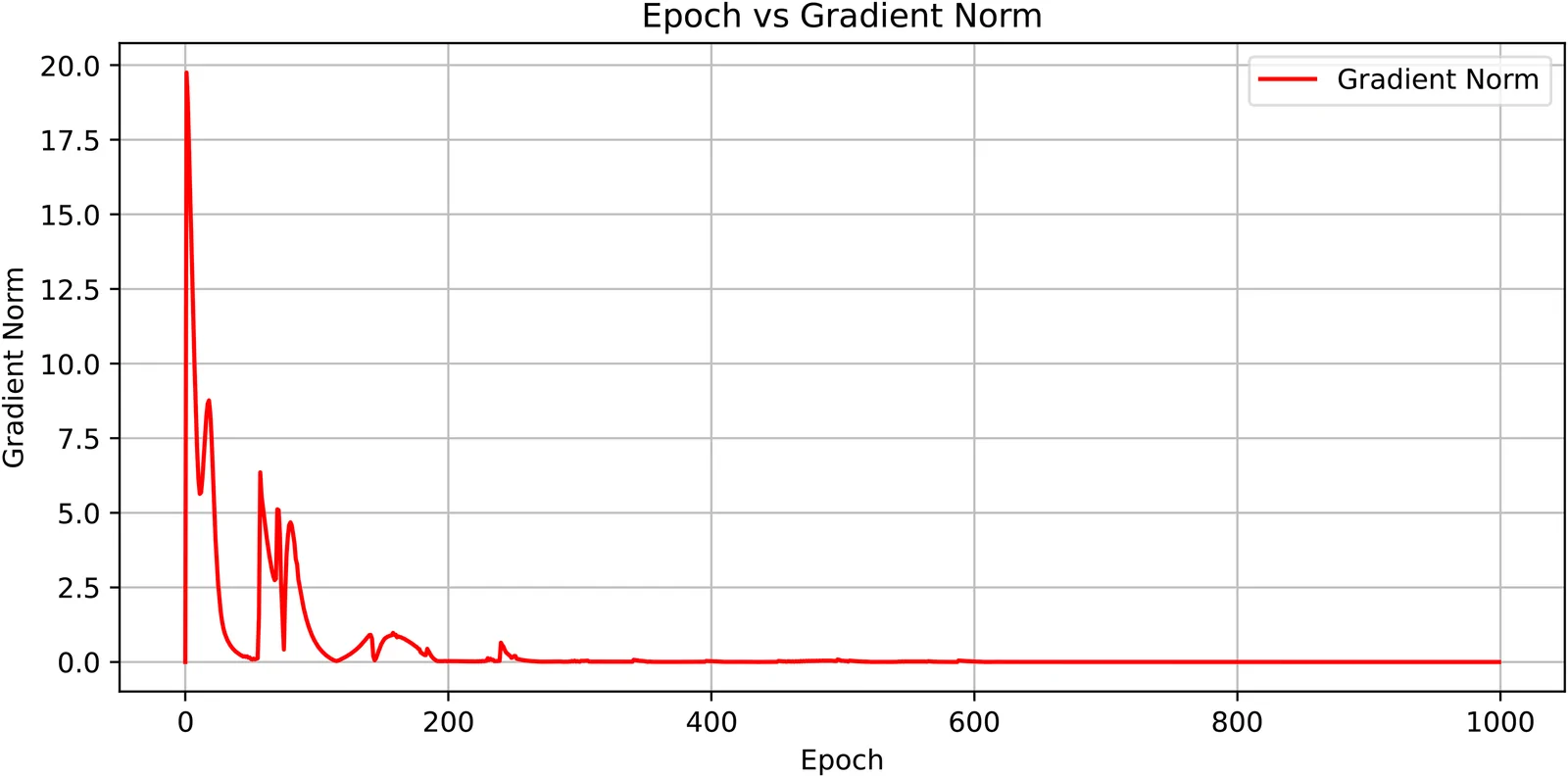
Gradient norm evolution during training for Case II. S-PINN gradient norm.

**Fig 24:**
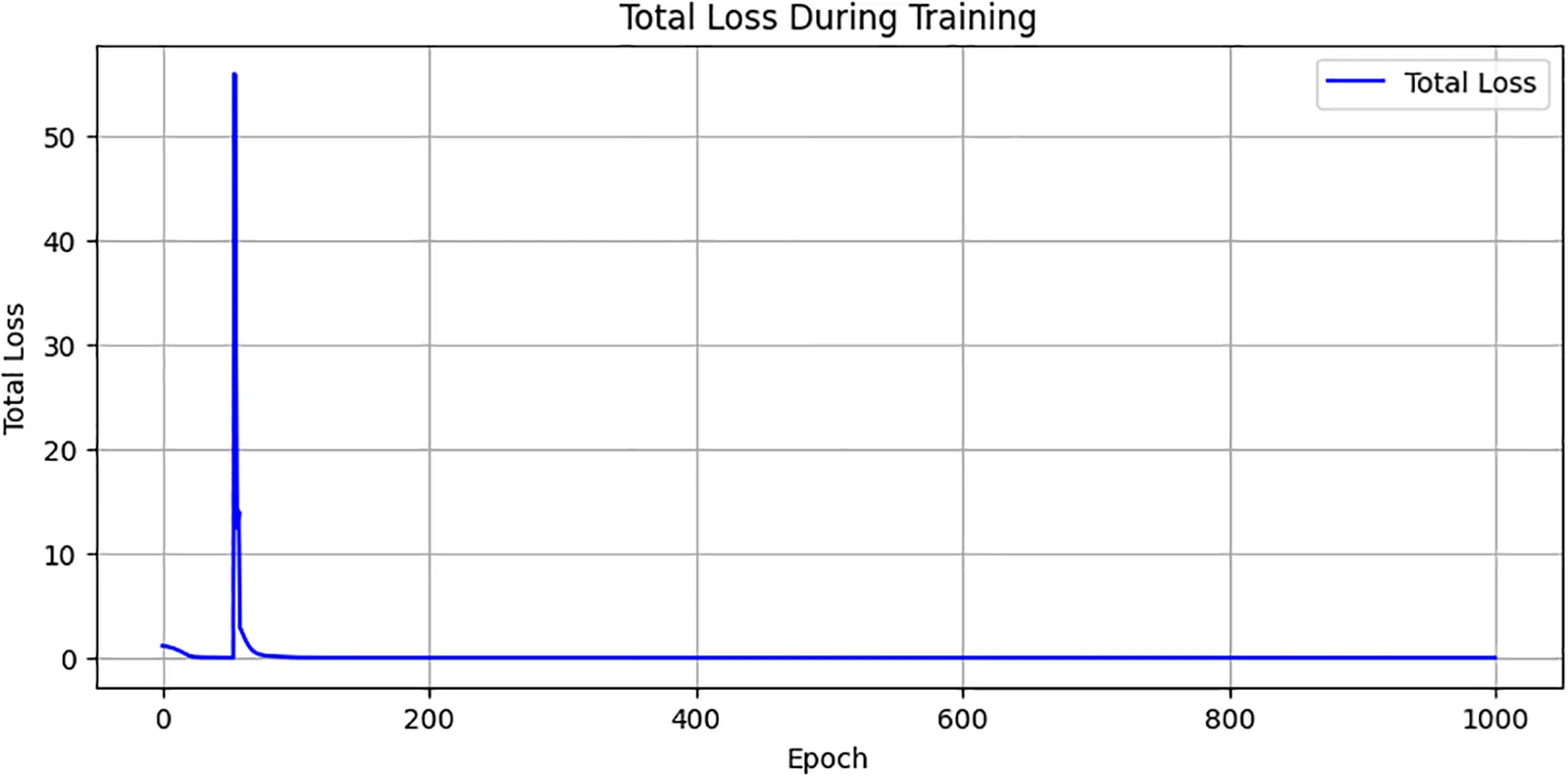
Gradient norm evolution during training for Case II. PINN gradient norm.

For the S-PINN, the gradient norm decreases rapidly after the initial training phase and then remains within a more stable range. This means that the largest parameter corrections occur at the beginning, when the network is adjusting itself to the reduced ODE and the symmetry constraint. After this stage, the optimizer requires only smaller corrections, indicating that the model has entered a more well-conditioned region of the loss landscape. This stable gradient behavior supports the earlier observations from the loss and update-cost plots.

The standard PINN shows less favorable gradient behavior. Since it lacks explicit symmetry guidance, the optimizer has to work harder to identify the correct solution structure. This can lead to larger gradient variations and less stable parameter updates. Such behavior is consistent with the gradient-pathology problem reported in PINN studies, where the imbalance between different components of the loss function can produce unstable or slow convergence. In the present case, the S-PINN reduces this difficulty by embedding the invariant structure directly into the learning process.

The improved gradient behavior of the S-PINN is important because it explains why the model performs better in the more complicated reduced system of Case II. The symmetry constraint acts as a stabilizing mechanism. It restricts the search space, reduces unnecessary gradient fluctuations, and helps the optimizer follow a more physically meaningful descent direction. Therefore, the gradient norm comparison confirms that the proposed S-PINN improves not only the final approximation but also the internal optimization dynamics.

#### 5.2.4 Overall discussion for case II.

The results of Case II demonstrate that the proposed S-PINN provides a clear improvement over the standard PINN when the governing PDE is reduced through the subalgebra $S^{3}=\langle Z_{1}+cZ_{2}\rangle$. This reduction produces a more demanding learning problem than Case I because the reduced ODE contains the combined influence of two symmetry generators. Despite this additional complexity, the S-PINN achieves smoother loss convergence, more controlled update-cost behavior, and more stable gradient evolution.

Compared with the classical PINN framework, the proposed S-PINN does not rely only on residual minimization. Instead, it incorporates the available symmetry information into the learning process. This additional mathematical structure reduces the effective solution space and guides the network toward physically admissible solutions. Compared with later PINN improvements that mainly focus on adaptive loss weighting or optimization stabilization, the present approach adds a structural improvement by preserving the invariance of the reduced system.

Therefore, the figures confirm that the S-PINN is more robust for Case II. The method improves convergence, reduces training irregularities, and provides better gradient conditioning. These improvements are particularly valuable for nonlinear reduced ODE systems, where standard PINNs may struggle because of stiffness, gradient imbalance, and loss competition. The proposed symmetry-preserving model therefore represents a stronger and more reliable alternative to conventional PINNs for learning reduced dynamics generated by Lie-symmetry subalgebras.

Fig 25 illustrates the evolution of the layer-wise gradient norm during the S-PINN training process. The gradient norms are shown on a logarithmic scale for six network layers, which allows the behavior of both large and small gradient magnitudes to be clearly observed. At the initial training stage, all layers exhibit a sharp increase in gradient magnitude, reaching values close to 10^2^. This indicates that the optimizer performs strong parameter corrections at the beginning of training in order to rapidly reduce the dominant residual and symmetry errors. After the initial transient phase, the gradient norms decrease significantly and enter a bounded oscillatory regime. These oscillations are expected in physics-informed optimization because the total loss contains several competing components, including the reduced ODE residual, the symmetry constraint, and the boundary or anchor loss. The repeated peaks around intermediate epochs show that the optimizer periodically re-adjusts the network parameters to balance these different physical constraints. However, the gradient values remain controlled throughout the training process, which confirms that no gradient explosion occurs after the early optimization phase. A layer-dependent pattern is also visible. The first layers generally maintain larger gradient norms than the deeper layers, indicating that the early layers are more active in learning the dominant features of the similarity-variable representation. In contrast, the deeper layers show smaller gradient magnitudes, especially during later epochs, suggesting that they perform more refined corrections to improve the final approximation. This behavior demonstrates stable gradient propagation across the network depth and indicates that the proposed S-PINN formulation avoids severe vanishing-gradient effects. Toward the final training stage, the gradient norms decrease to approximately 10^–3^–10^–2^ for most layers, with only small residual oscillations. This shows that the optimizer approaches a stable solution while still preserving enough gradient activity for fine-scale parameter updates. Overall, the layer-wise gradient norm evolution confirms that the symmetry-preserving S-PINN framework maintains stable and well-balanced training dynamics across all layers, supporting the robustness of the proposed learning strategy.

**Fig 25:**
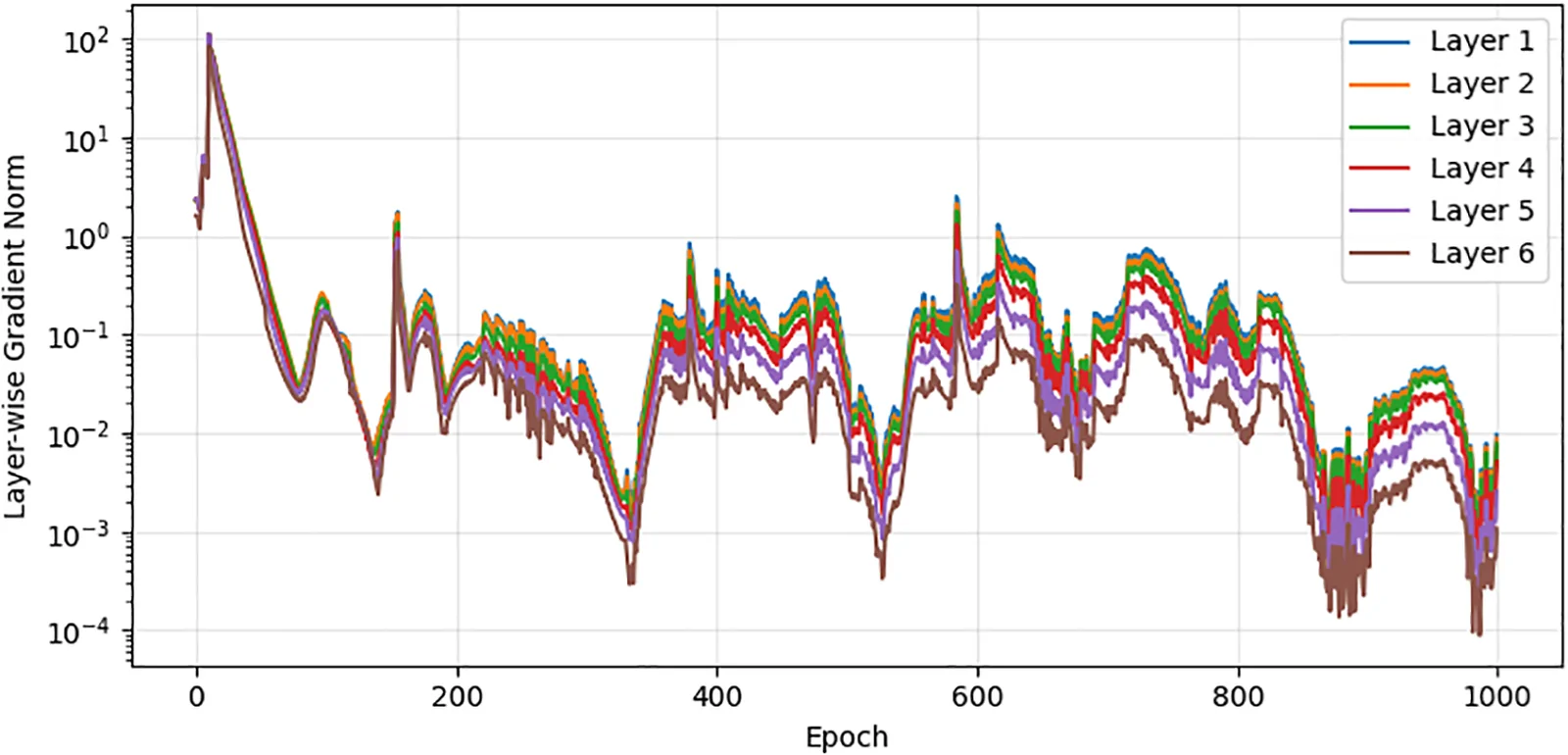
Layer-wise gradient norm evolution during S-PINN training.

#### 5.2.5 ODE solution comparison.

Fig 26 compares the exact analytical solution with the numerical predictions obtained from the standard PINN and the proposed S-PINN. This comparison is important because it directly shows whether the trained model can preserve the translation-invariant structure generated by the subalgebra $S^{3}=\langle Z_{1}+cZ_{2}\rangle$. In this case, the invariant structure is related to the condition $\partial_{x}v+c,\partial_{t}v=0,$ which means that the solution should evolve along a symmetry-preserving direction in the (*x*,*t*)-plane.

**Fig 26:**
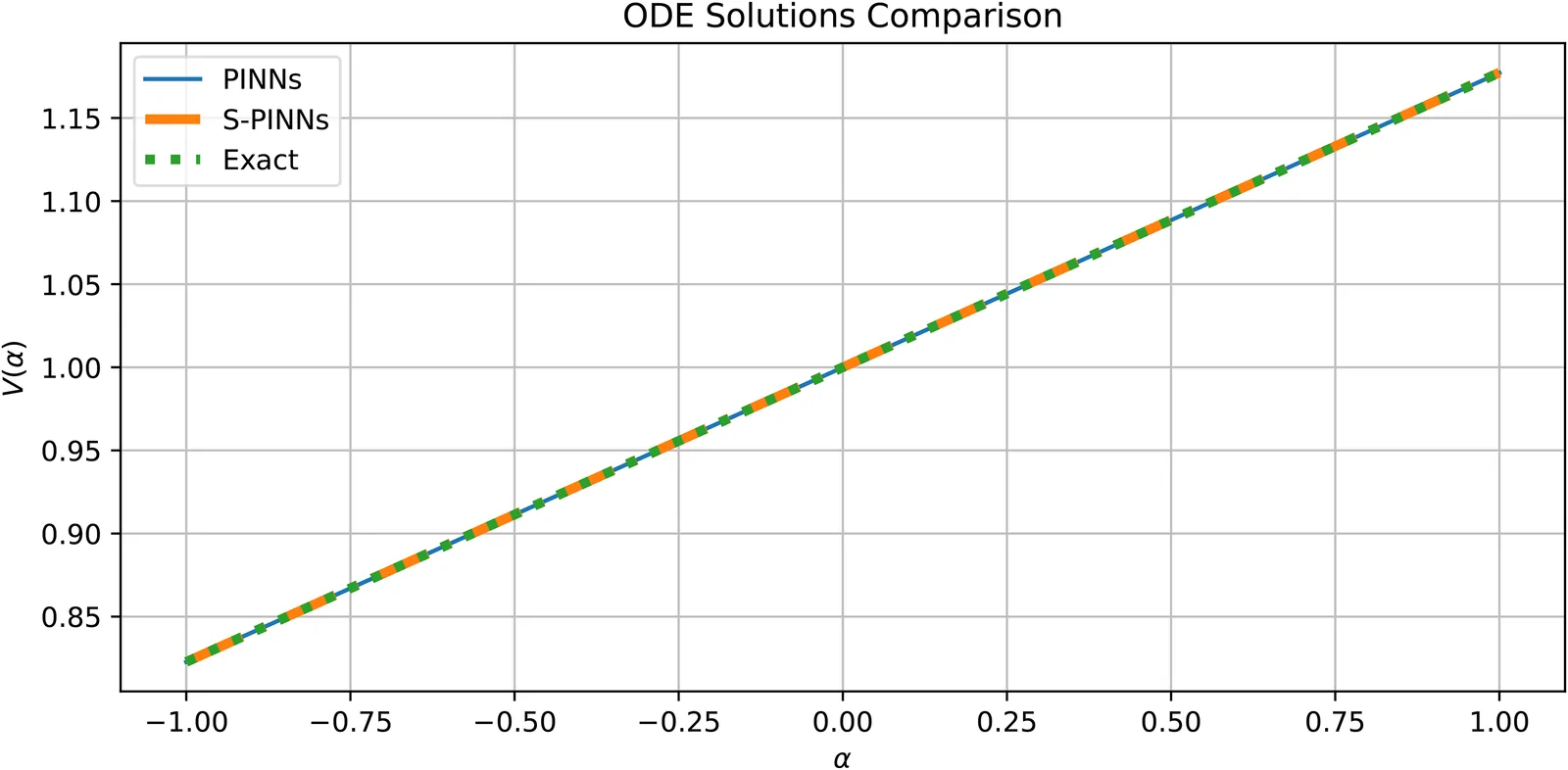
Comparative analysis of the reduced ODE solution profiles obtained through the translation-type symmetry reduction generated by $S^{3}=\langle Z_{1}+cZ_{2}\rangle$. The exact analytical solution is represented by the black dotted curve, the S-PINN approximation by the blue solid curve, and the standard PINN prediction by the red dashed curve.

The S-PINN solution follows the exact analytical profile very closely over the whole computational interval. The blue curve remains almost attached to the black dotted curve, which confirms that the additional symmetry loss $L\_{sym}^{\left(2\right)}$ helps the network learn the correct invariant solution family. This agreement is especially meaningful in the middle part of the domain, where the solution variation is more sensitive and small errors in the learned curvature may lead to visible deviations. The ability of S-PINN to remain stable in this region shows that the model is not simply fitting pointwise values, but is also learning the underlying mathematical structure of the reduced ODE.

In contrast, the standard PINN captures the general trend of the exact solution but shows noticeable deviation from the reference profile. The red dashed curve moves away from the exact solution in the mid-range of the domain and continues to show a small mismatch as the independent variable increases. This behavior indicates that the standard PINN can minimize the residual loss, but it does not automatically preserve the symmetry-induced solution structure. Since the standard PINN is trained mainly through the PDE residual and boundary/initial information, it has to discover the invariant behavior indirectly. This makes the learning process more difficult, particularly for reduced equations generated by combined symmetry operators.

This observation is consistent with the classical PINN literature. The original PINN framework showed that neural networks can solve forward and inverse differential equation problems by embedding the governing equations into the loss function. However, later studies reported that standard PINNs may suffer from slow convergence, loss imbalance, and gradient-related training difficulties when the solution structure is nonlinear or highly constrained. The present S-PINN improves on this limitation by adding symmetry information as a mathematical guide. Similar improvements have also been reported in symmetry-enhanced and Lie-symmetry-informed PINNs, where invariant surface conditions or Lie generators are incorporated into the loss function to improve accuracy, generalization, and sample efficiency. Therefore, Fig 26 confirms that the proposed S-PINN is more accurate and more physically consistent than the conventional PINN for Case II.

#### 5.2.6 Surface and contour plots.

Figs 27–29 and 30–32 provide a broader view of the learned solution in the original computational domain. While the ODE profile in Fig 26 verifies the solution along the reduced similarity variable, the surface and contour plots show whether the neural network can reconstruct the global solution pattern in the full (*x*,*t*)-domain. This is a stronger test because a model may appear accurate along one curve but still fail to preserve the correct global geometry of the solution field.

**Fig 27:**
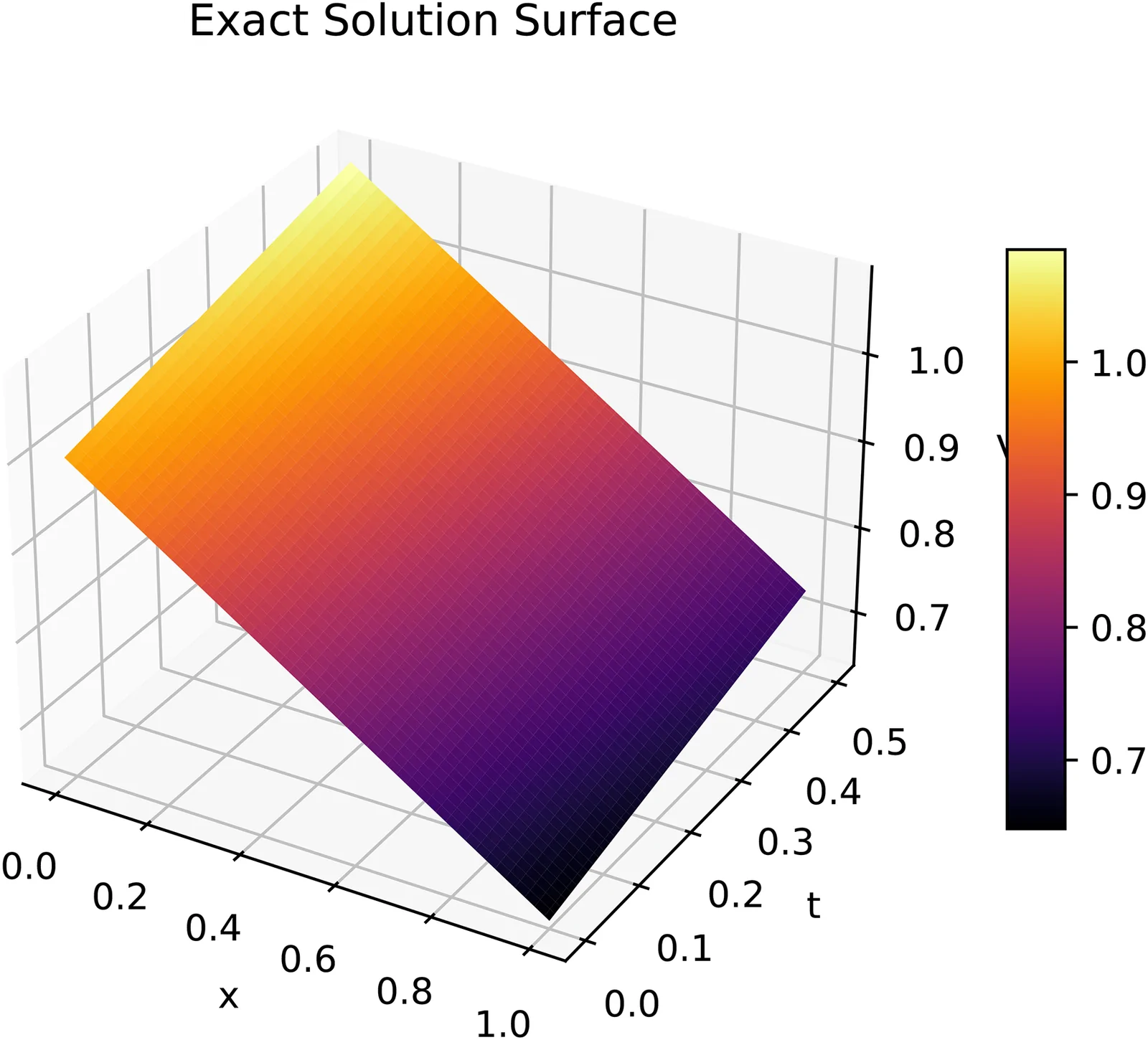
Surface solution comparison for Case II. Exact surface.

**Fig 28:**
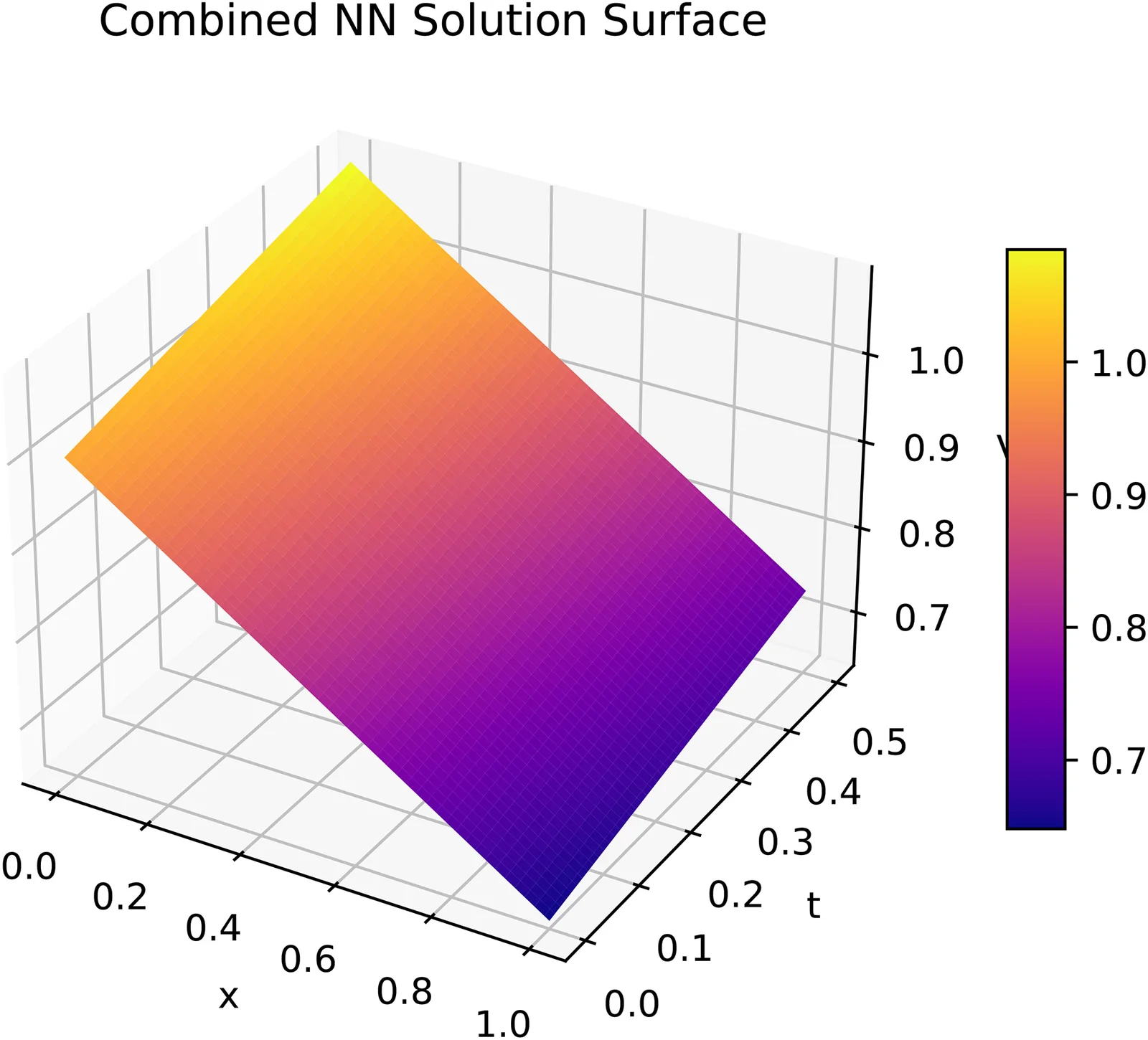
Surface solution comparison for Case II. Standard PINN surface.

**Fig 29:**
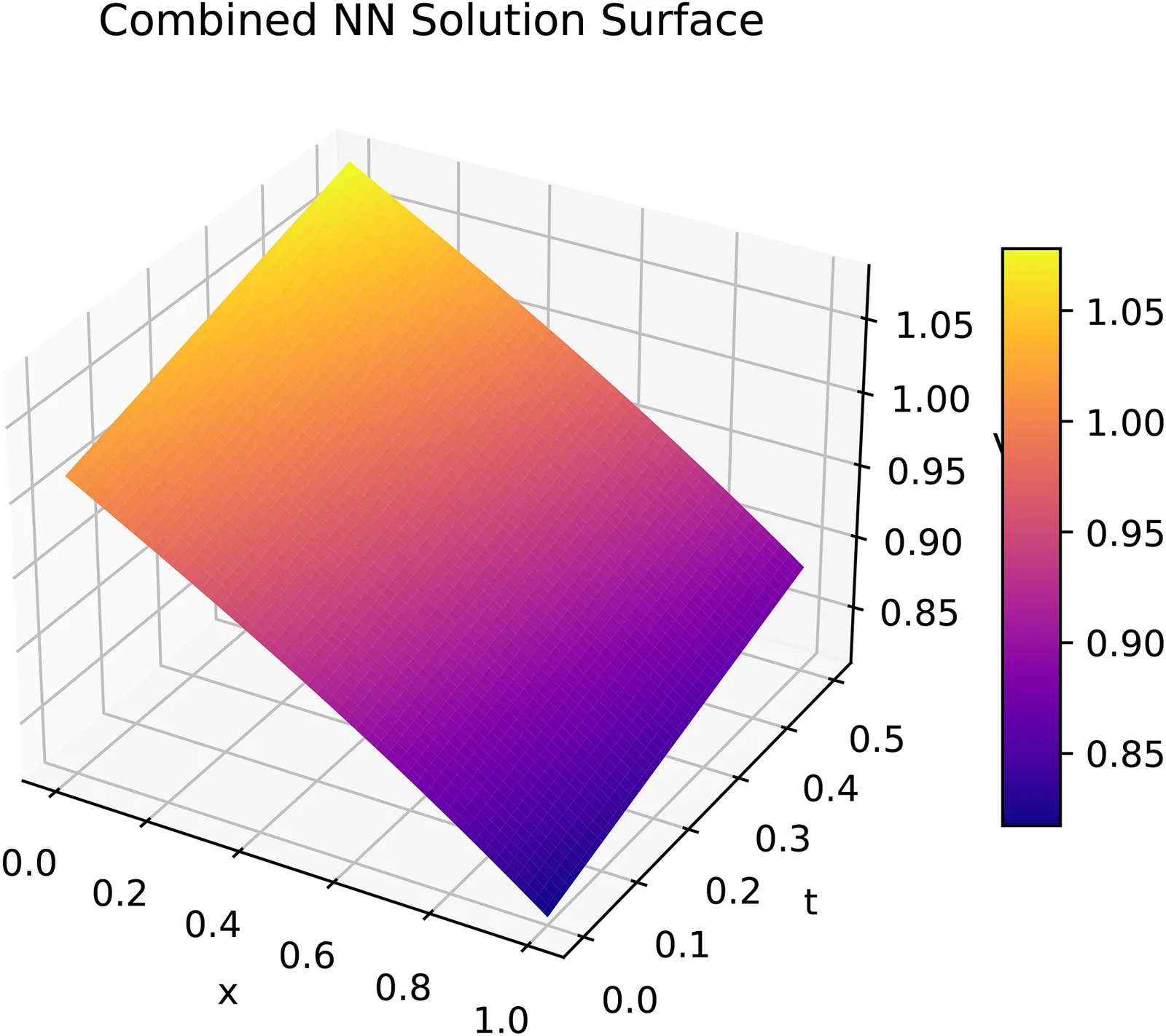
Surface solution comparison for Case II. S-PINN surface.

**Fig 30:**
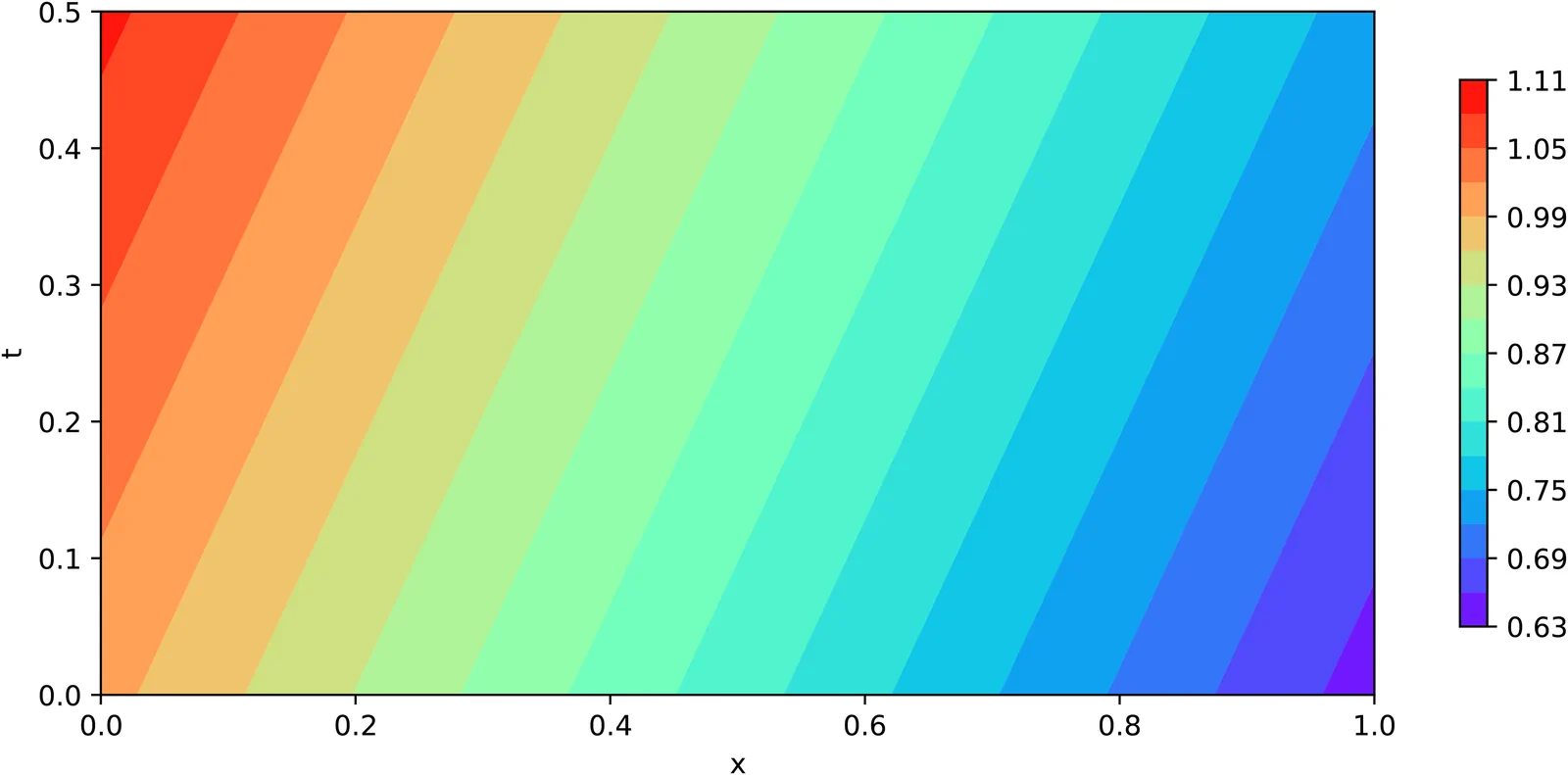
Contour solution comparison for Case II. Exact contour.

**Fig 31:**
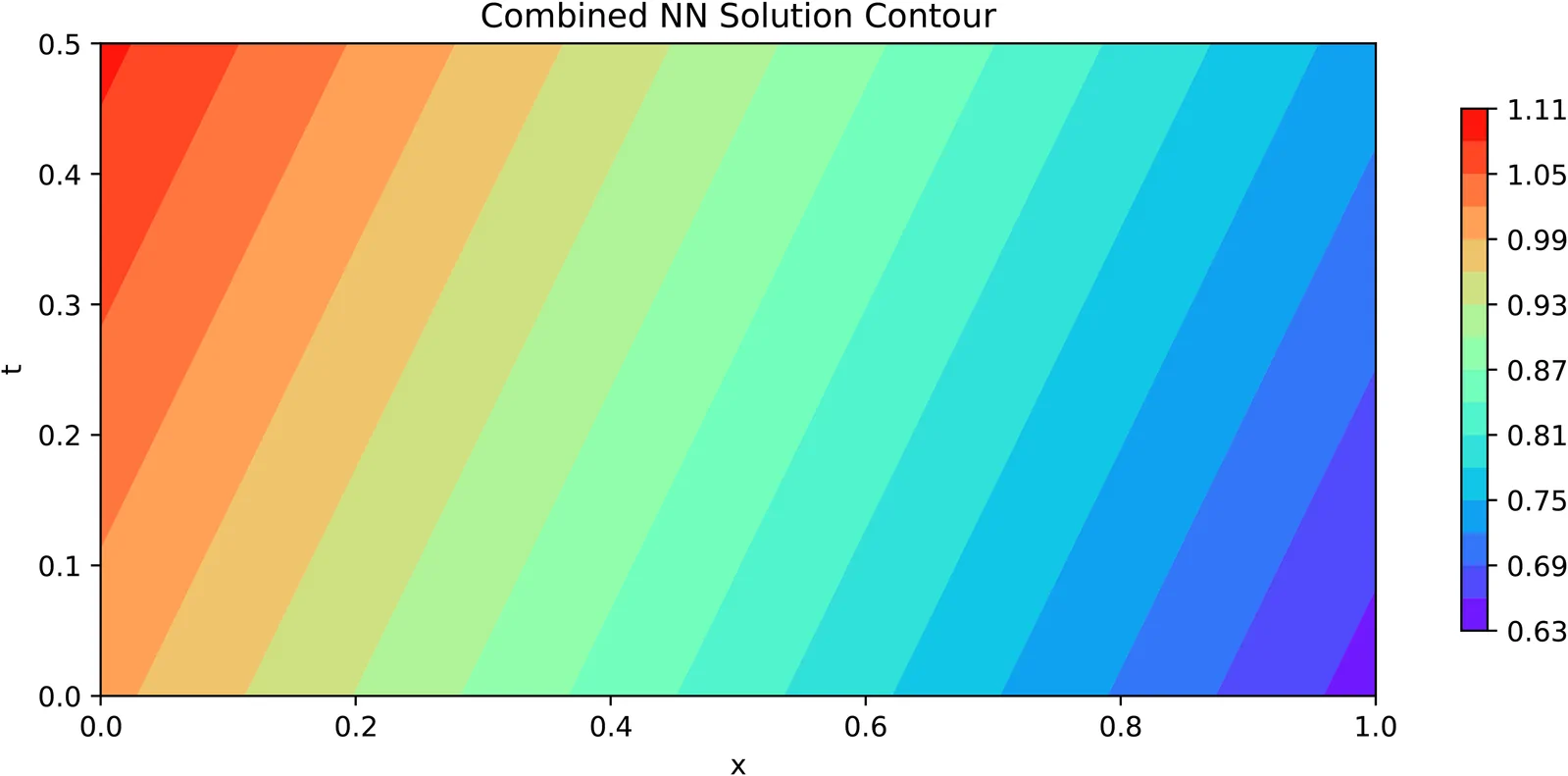
Contour solution comparison for Case II. Standard PINN contour.

**Fig 32:**
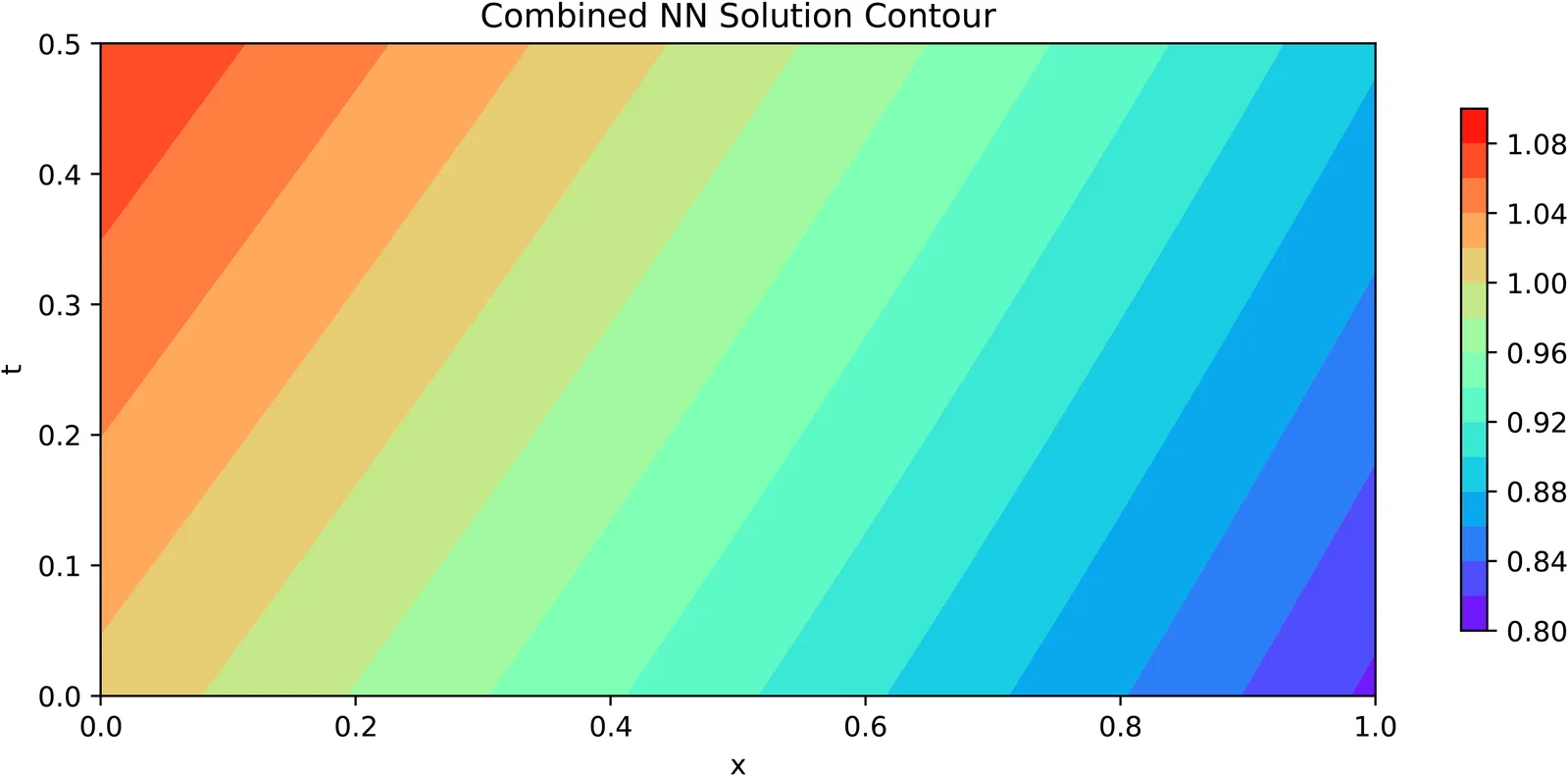
Contour solution comparison for Case II. S-PINN contour.

The exact surface in Fig 27 shows a smooth and coherent solution structure. The surface varies regularly without artificial oscillations, abrupt jumps, or distorted ridges. This smooth behavior is expected from the analytical solution and serves as the reference pattern for evaluating both neural network models. The corresponding exact contour plot in Fig 30 shows well-organized contour bands. These bands follow the translation-symmetry direction and reflect the invariant nature of the reduced solution.

The standard PINN surface in Fig 28 reproduces the general solution trend, but the surface is less consistent with the exact geometry. Some regions show weaker curvature representation and less accurate amplitude distribution. This indicates that the standard PINN learns the dominant solution behavior but does not fully preserve the invariant surface structure. The same limitation can also be observed in Fig 31, where the contour bands are less aligned with the exact contour pattern. This means that the standard PINN may satisfy the residual approximately while still producing a solution field that is geometrically less faithful to the exact symmetry pattern.

The S-PINN surface in Fig 29 shows a stronger agreement with the exact solution surface. The reconstructed surface is smoother, more organized, and more consistent with the translation-invariant structure of the problem. Similarly, the contour plot in Fig 32 preserves the main direction, spacing, and regularity of the exact contour bands. This confirms that the S-PINN is able to learn not only the numerical values of the solution but also the geometry of the symmetry-reduced solution field.

This improvement is directly related to the recent development of structure-preserving PINNs. Standard PINNs use the governing residual as a soft constraint, but they do not necessarily encode the geometric or symmetry properties of the differential equation. Because of this, the model may require more training points or a stronger optimizer to recover physically meaningful patterns. In contrast, symmetry-enhanced PINNs embed invariant conditions into the loss function, which restricts the neural approximation to a more physically admissible solution space. The present surface and contour results support this idea. By enforcing the translation symmetry associated with $Z_{1}+cZ_{2}$, the S-PINN improves the global shape of the solution and reduces the mismatch observed in the standard PINN output.

#### 5.2.7 Loss components.

Figs 33 and 34 presents the decomposition of the S-PINN training loss into PDE-related and symmetry-related components. This figure is important because the total loss alone cannot show how the network balances different physical and structural constraints during training. In physics-informed learning, the loss function usually contains several terms, and poor balance among these terms can lead to unstable convergence or inaccurate solutions.

**Fig 33:**
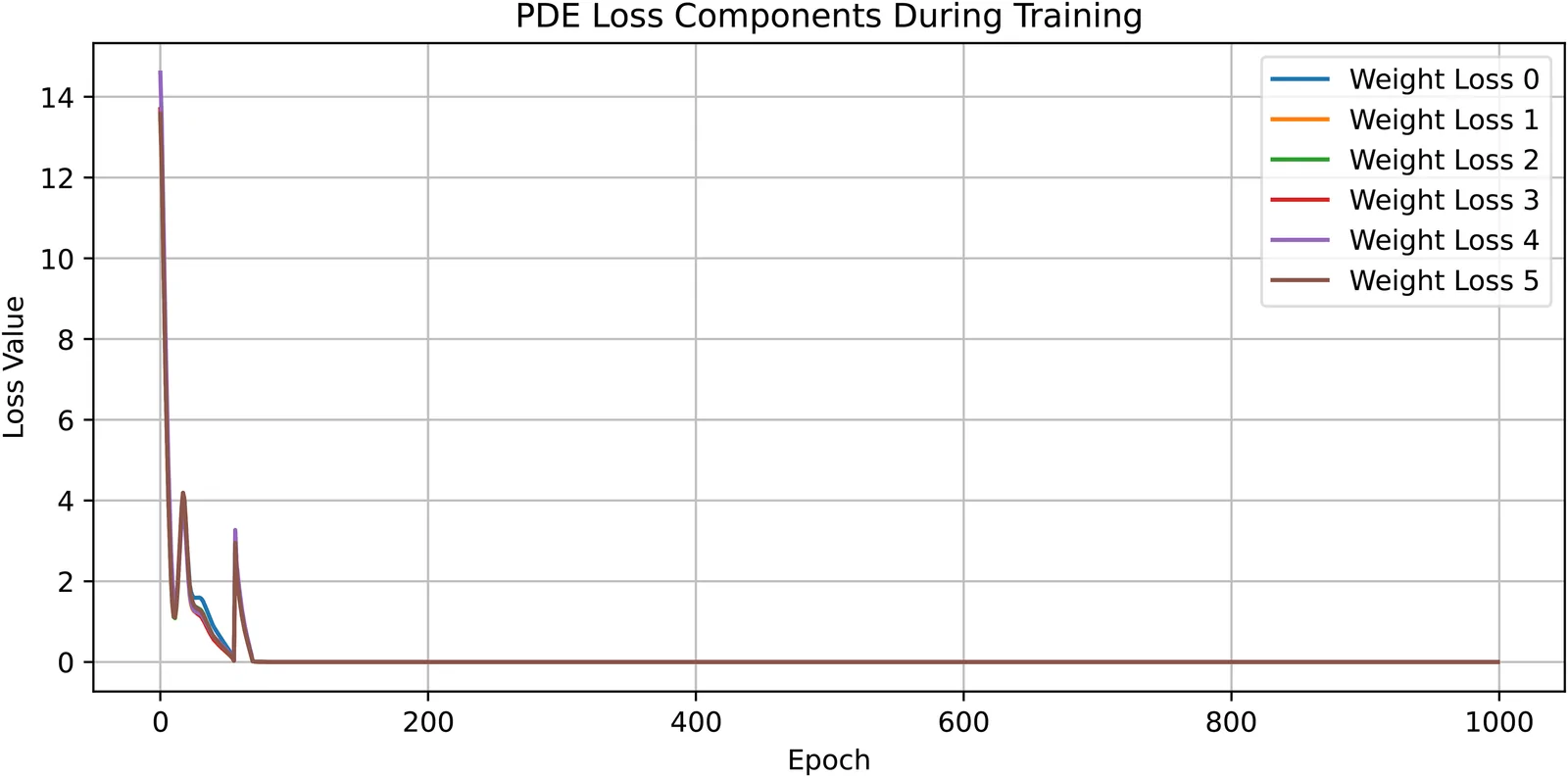
S-PINN loss component analysis for Case II. PDE loss components.

**Fig 34:**
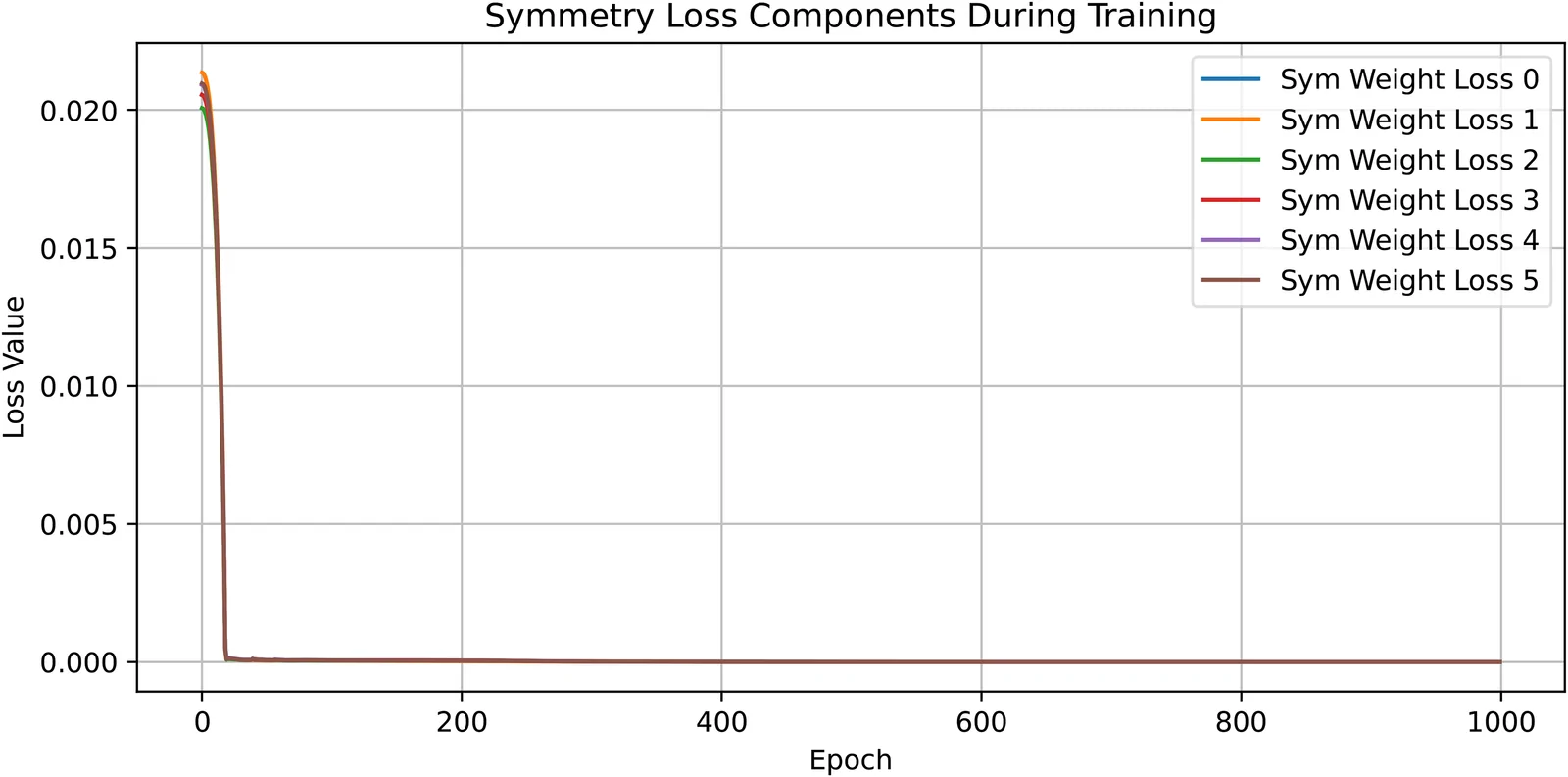
S-PINN loss component analysis for Case II. Symmetry loss components.

The PDE loss components in Fig 33 decrease in a coordinated manner as training progresses. This means that the network does not reduce one part of the governing residual while neglecting the others. Such balanced decay is a positive sign because it shows that the S-PINN is learning the reduced equation in a stable and consistent way. The absence of a dominant residual term also suggests that the optimization process is well-conditioned after the early epochs.

The symmetry loss components in Fig 34 also decrease steadily. At the beginning of training, these components drop rapidly, which indicates that the network quickly learns the main translation-invariant structure. After this initial stage, the decrease becomes more gradual, showing that the model performs finer corrections to improve symmetry enforcement. This two-stage behavior is physically reasonable: first, the model identifies the broad invariant pattern, and then it refines the solution so that both the PDE residual and the symmetry constraint are satisfied more accurately.

This loss behavior addresses one of the common difficulties of standard PINNs. Previous studies showed that PINNs can suffer from gradient pathologies because the different terms in the composite loss may produce unbalanced back-propagated gradients. Their later NTK-based analysis also showed that different loss components may converge at different rates, which can explain why some PINNs train slowly or fail to learn certain parts of the solution. In the present S-PINN, the symmetry term helps regularize the training process by guiding the network toward a smaller and more structured solution space. Therefore, the balanced decay of the PDE and symmetry losses confirms that the proposed model improves the internal training dynamics compared with the standard residual-based PINN.

#### 5.2.8 Weight evolution.

Figs 35 and 36 shows how the trainable weights evolve during the S-PINN training process. The weight history provides useful information about the stability of the optimizer. If the weights show persistent large oscillations, it usually means that the network is struggling to balance the different parts of the loss function. On the other hand, smooth stabilization of the weights indicates that the optimizer is moving toward a stable region of the loss landscape.

**Fig 35:**
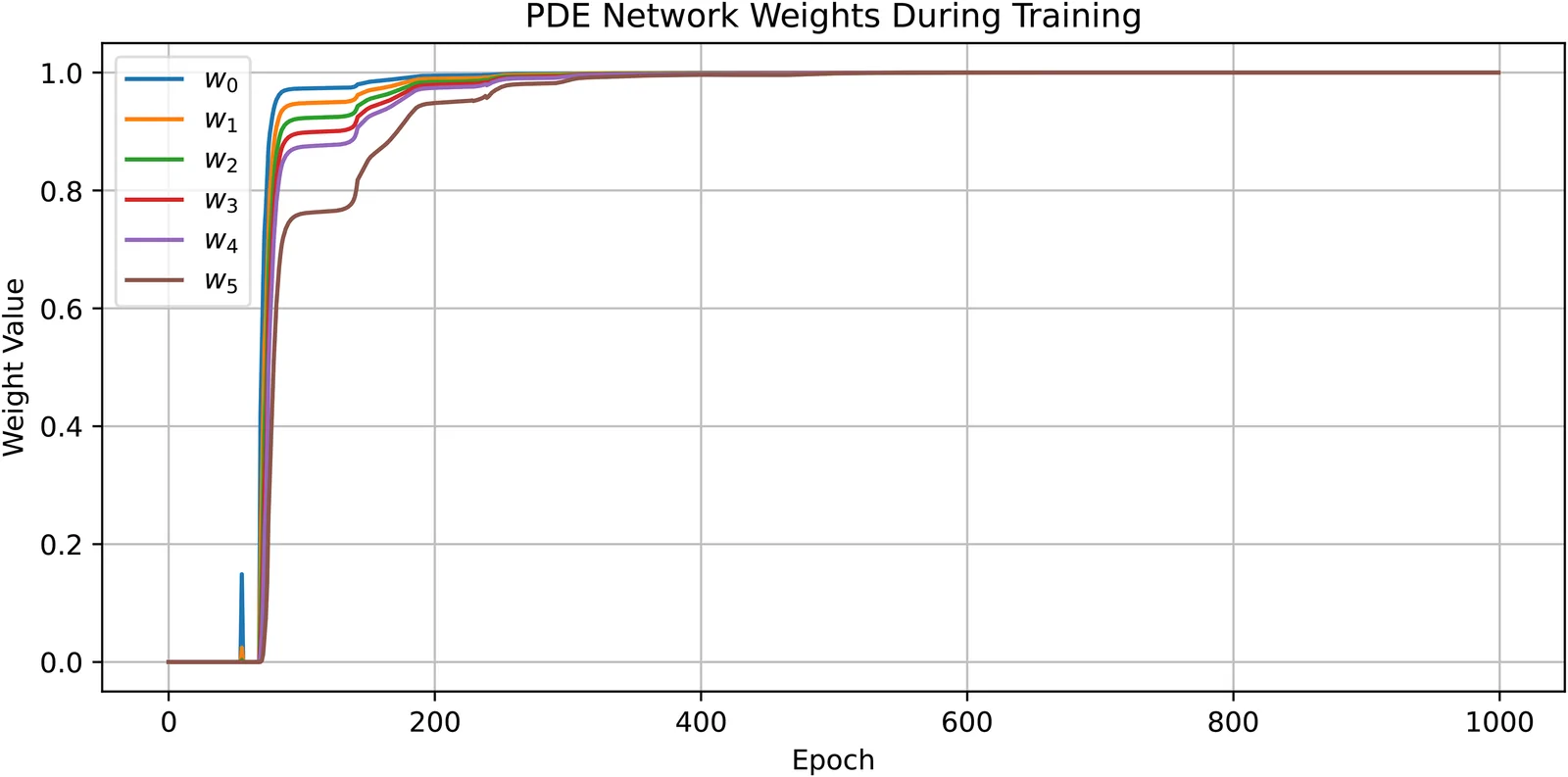
Weight evolution during S-PINN training for Case II. PDE network weights.

**Fig 36:**
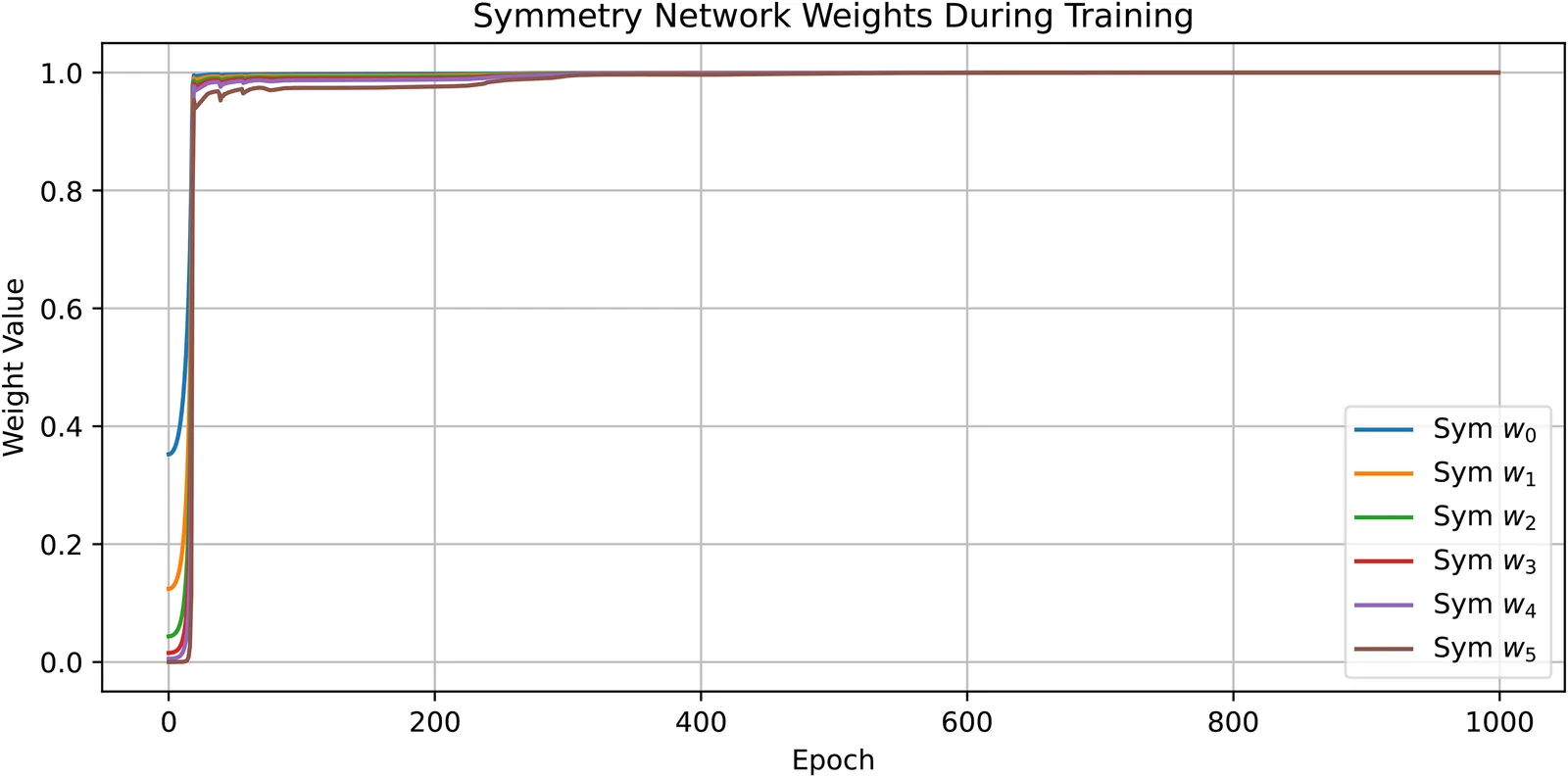
Weight evolution during S-PINN training for Case II. Symmetry network weights.

The PDE network weights in Fig 35 show a gradual adjustment pattern. This means that the part of the model responsible for satisfying the governing equation residual learns progressively and does not suffer from severe instability. Some mild changes during the early epochs are expected because the network initially adjusts its parameters to represent the reduced ODE solution. However, as training proceeds, the weights become more stable, showing that the PDE residual is being learned in a controlled way.

The symmetry network weights in Fig 36 stabilize more clearly after the early learning stage. This indicates that the model internalizes the translation-symmetry condition relatively early in the optimization process. Once the invariant direction is learned, the symmetry-related weights require only smaller corrections. This behavior explains why the S-PINN produces a more accurate ODE profile and more organized surface and contour patterns. The symmetry component acts as a regularizing mechanism that prevents the network from drifting toward solutions that may reduce the residual but violate the invariant structure.

The weight evolution results also connect well with earlier studies on symmetry-preserving neural solvers. Previous studies showed that adding invariant surface conditions to the PINN loss can improve accuracy and reduce the need for larger training sets or more complex architectures. Other studies also emphasized that Lie-point-symmetry information can improve the sample efficiency of PINN-type solvers by teaching the network neighboring symmetry-generated solutions. The present weight evolution supports these findings. The S-PINN does not merely improve the final error; it improves the route through which the model learns the solution.

#### 5.2.9 Overall discussion for case II.

The results of Case II clearly demonstrate the benefit of incorporating the translation symmetry generated by $S^{3}=\langle Z_{1}+cZ_{2}\rangle$ into the physics-informed learning framework. The ODE solution comparison shows that the S-PINN prediction remains closer to the exact analytical solution than the standard PINN. The surface and contour plots further confirm that the S-PINN reconstructs the global solution geometry more accurately and preserves the symmetry-aligned structure of the exact solution. The loss-component analysis shows that the PDE and symmetry losses decrease in a balanced way, while the weight evolution confirms that the symmetry-related learning process stabilizes during training.

Compared with the original PINN framework, the proposed S-PINN does not depend only on residual minimization. Instead, it includes the known invariant condition as an additional mathematical constraint. This makes the learning problem more structured and reduces unnecessary exploration of the solution space. Compared with studies that focus mainly on optimizer tuning or adaptive loss balancing, the present approach improves the model at the structural level by embedding the symmetry of the governing equation directly into the training process.

Therefore, the proposed S-PINN offers a clear improvement over previous standard PINN models for Case II. It provides better agreement with the exact solution, stronger preservation of the global solution geometry, more balanced loss convergence, and more stable parameter evolution. These results confirm that symmetry preservation is a powerful strategy for improving the accuracy, stability, and physical reliability of PINNs, especially for nonlinear reduced systems obtained through Lie-symmetry reductions.

Fig 37 shows the layer-wise gradient norm evolution during the S-PINN training process for all nine network layers. The logarithmic scale clearly illustrates the variation of gradient magnitudes over several orders of magnitude. At the beginning of training, the gradient norms rapidly increase and reach values close to 10^2^, indicating strong initial parameter corrections by the optimizer. This large early gradient response is expected because the network parameters are still far from satisfying the reduced residual equation, the symmetry constraint, and the imposed physical conditions. After the initial transient stage, the gradient norms decrease sharply and enter a bounded oscillatory regime. Several localized peaks can be observed, particularly around the early and intermediate epochs. These peaks represent temporary increases in optimization activity as the network attempts to balance the competing contributions of the residual loss, symmetry loss, and boundary or anchor loss. However, the magnitude of these peaks gradually decreases as training proceeds, which indicates that the optimization trajectory becomes more stable with increasing epochs. A clear layer-dependent behavior is also visible. The earlier and middle layers generally exhibit larger gradient magnitudes compared with the deeper layers, suggesting that they are more actively involved in learning the dominant representation of the reduced similarity solution. The deeper layers, especially the last layers, show smaller gradient values during the later stages of training, which indicates that they mainly perform fine-scale corrections rather than large structural updates. This layered behavior confirms that the network distributes the learning process across different depths in a stable manner. Toward the final epochs, most layer-wise gradient norms remain between approximately 10^–3^ and 10^–2^, with only mild oscillations. This behavior shows that the optimizer approaches a stable region of the loss landscape without suffering from severe gradient explosion or complete gradient collapse. Therefore, the layer-wise gradient norm evolution supports the stability and robustness of the proposed S-PINN training process. The bounded gradient propagation across all nine layers confirms that the symmetry-preserving formulation provides a well-conditioned optimization path for learning the reduced nonlinear dynamics.

**Fig 37:**
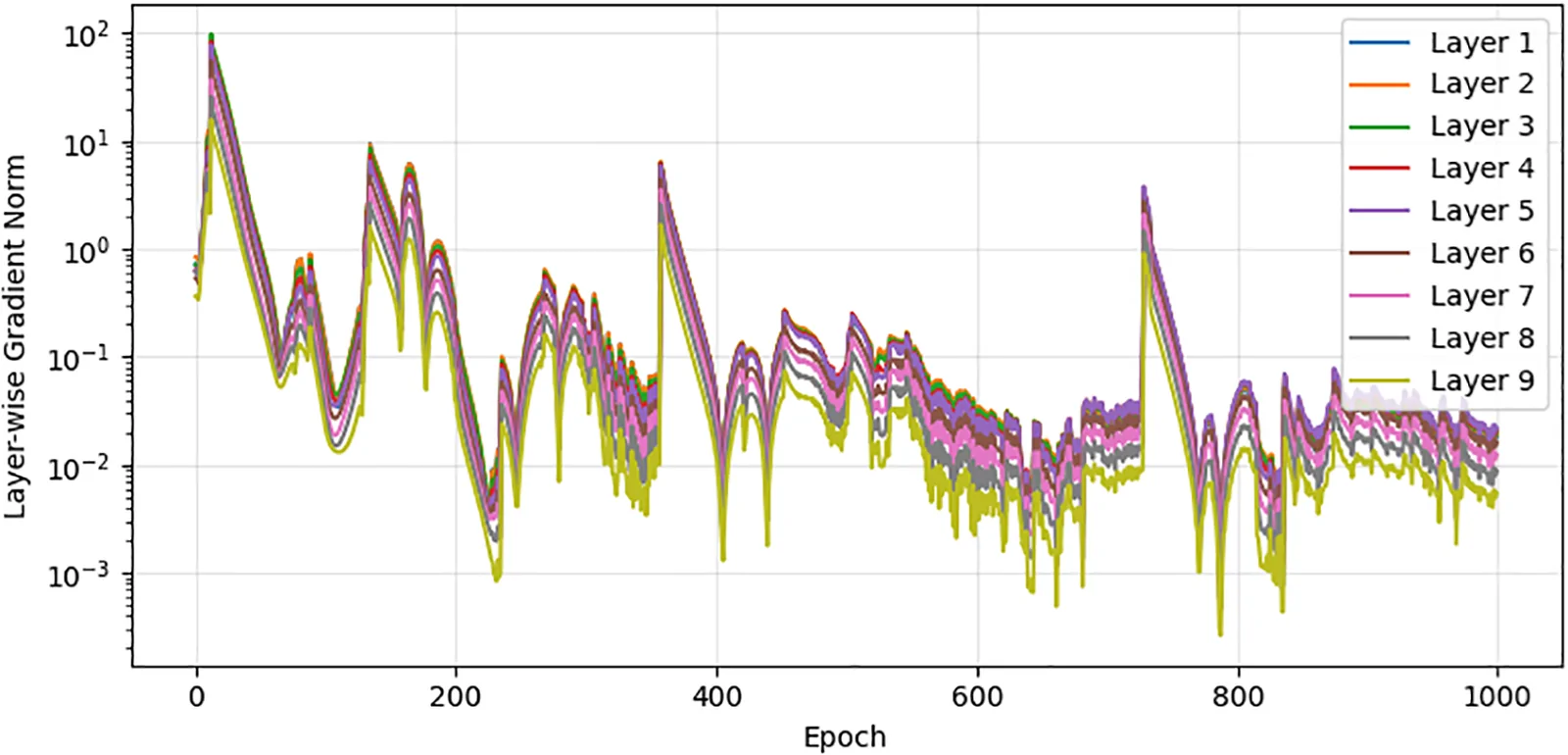
Layer-wise gradient norm evolution during S-PINN training.

#### 5.2.10 ODE solution comparison.

Fig 38 compares the exact solution with the predictions obtained from the standard PINN and the proposed S-PINN. This figure is important because it directly evaluates the quality of the learned reduced solution. The S-PINN prediction remains very close to the exact solution throughout the computational interval. The agreement is especially clear near the boundary regions, where standard PINNs often show larger deviations due to weak enforcement of structural information.

**Fig 38:**
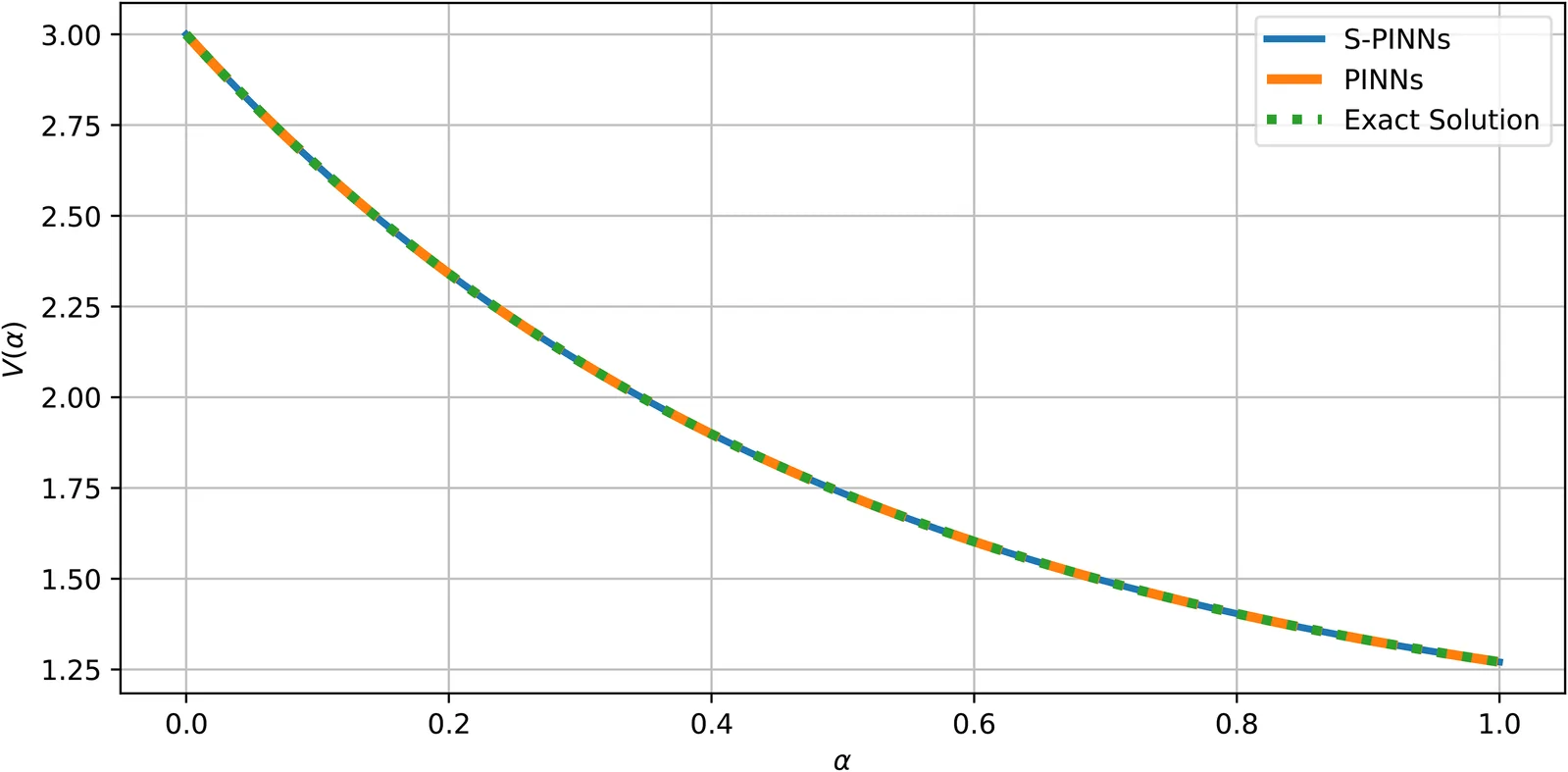
Solution accuracy comparison for Case III. The exact analytical solution is compared with the S-PINN and standard PINN approximations.

The standard PINN captures the main trend of the solution, but its curve shows visible departure from the exact profile in sensitive parts of the domain. These deviations indicate that the standard PINN is able to reduce the governing residual, but it does not fully preserve the structure of the exact solution. In contrast, the S-PINN benefits from the additional symmetry constraint, which guides the network toward the physically admissible solution family. As a result, the S-PINN not only approximates the numerical values more accurately, but also preserves the qualitative behavior of the solution.

This improvement is consistent with the known limitations of classical PINNs. The original PINN framework relies on minimizing the PDE residual together with boundary or initial losses. Although this approach is flexible, later studies showed that standard PINNs may suffer from slow convergence, gradient imbalance, and difficulty in learning constrained nonlinear solutions. The present S-PINN improves on these earlier models by adding symmetry information as an extra mathematical guide. In this way, the network searches in a smaller and more physically meaningful solution space, which explains the closer agreement observed in Fig 38.

#### 5.2.11 Surface and contour plots.

Figs 39–41 and 42–44 provide a global view of the learned solution over the computational domain. While the ODE comparison in Fig 38 evaluates the solution along the reduced variable, the surface and contour plots show whether the trained model can reconstruct the full solution geometry. This is a stronger test because a model may perform well along one profile but still fail to reproduce the correct surface structure.

**Fig 39:**
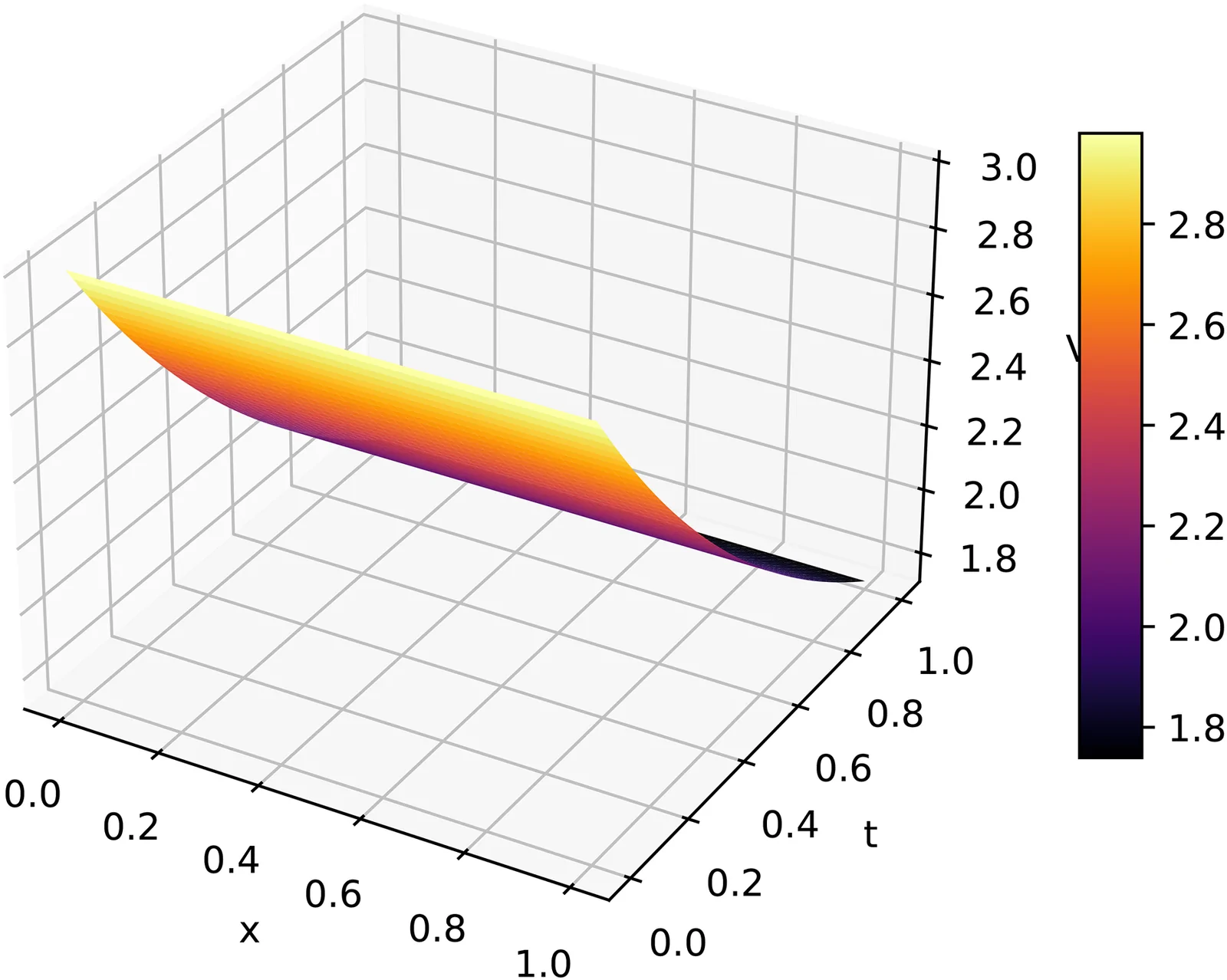
Surface solution comparison for Case III. Exact surface.

**Fig 40:**
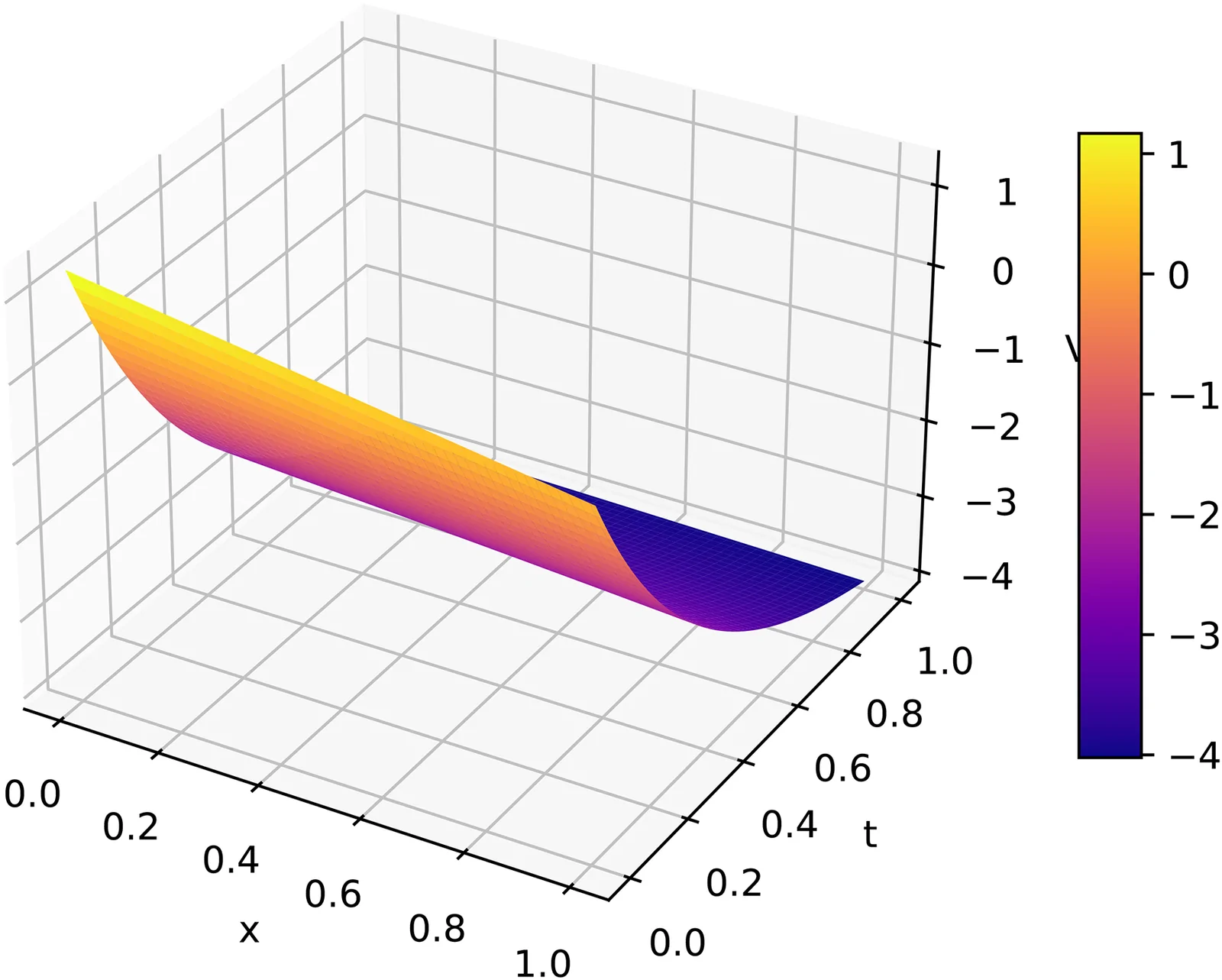
Surface solution comparison for Case III. S-PINN surface.

**Fig 41:**
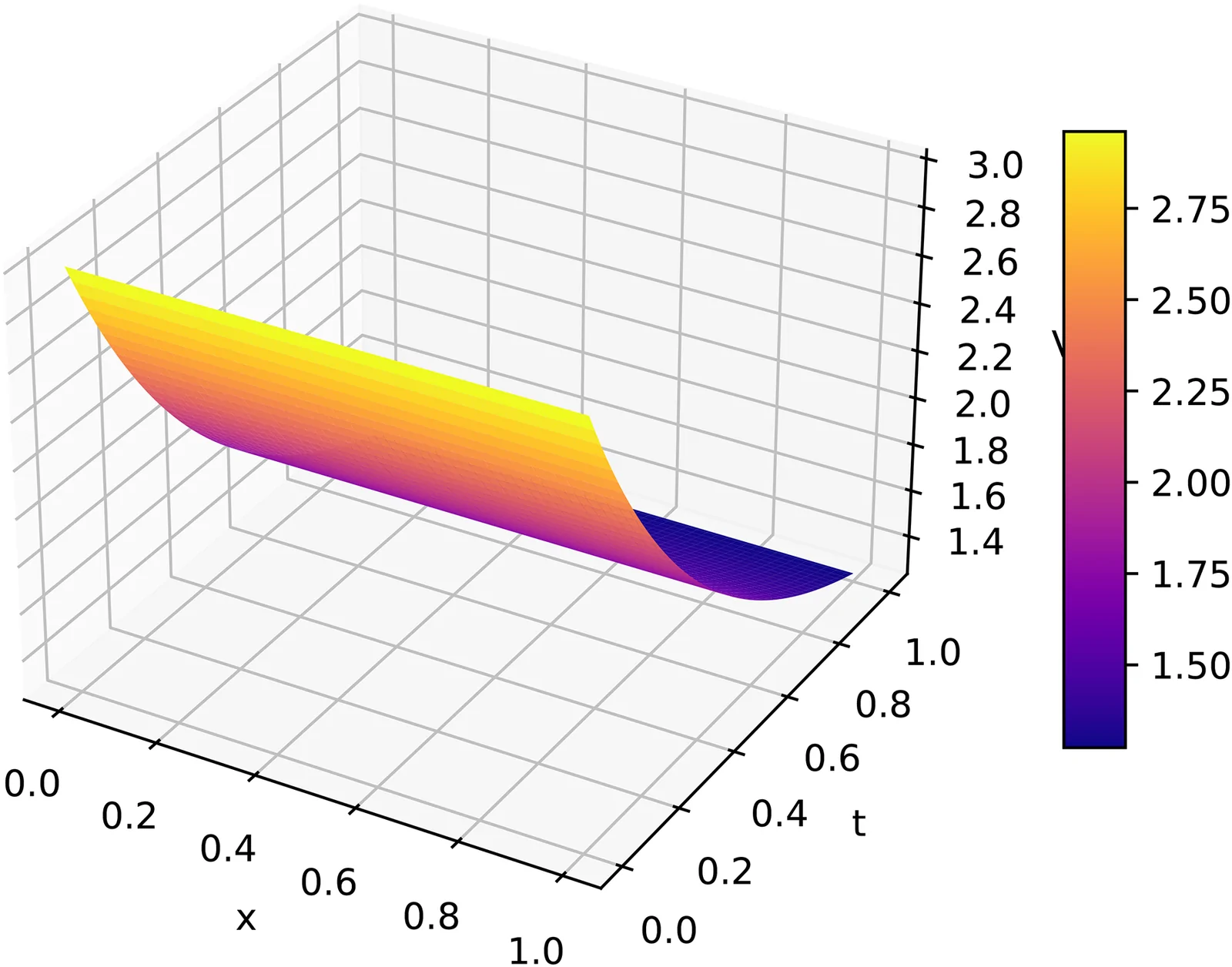
Surface solution comparison for Case III. PINN surface.

**Fig 42:**
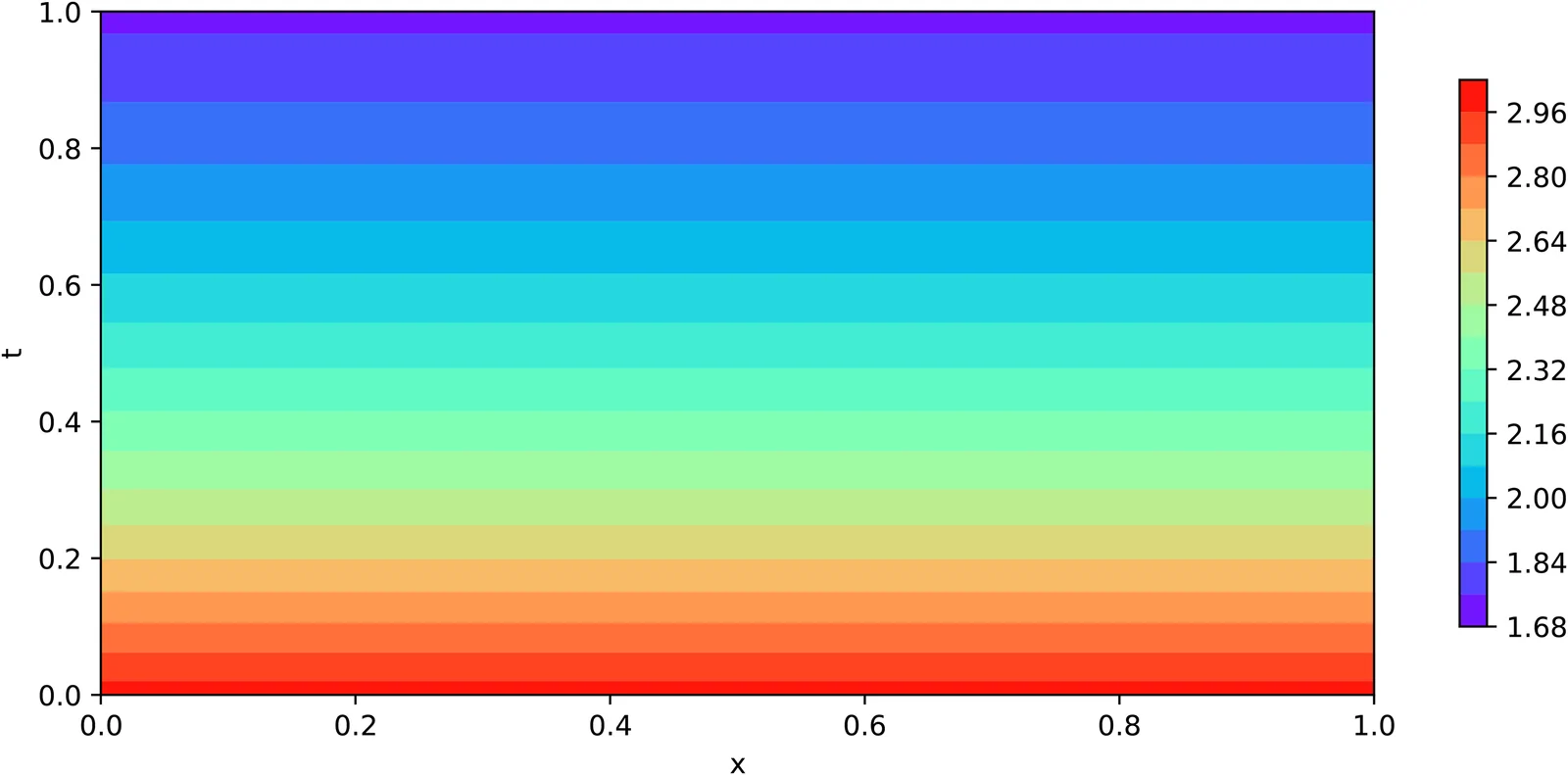
Contour solution comparison for Case III. Exact contour.

**Fig 43:**
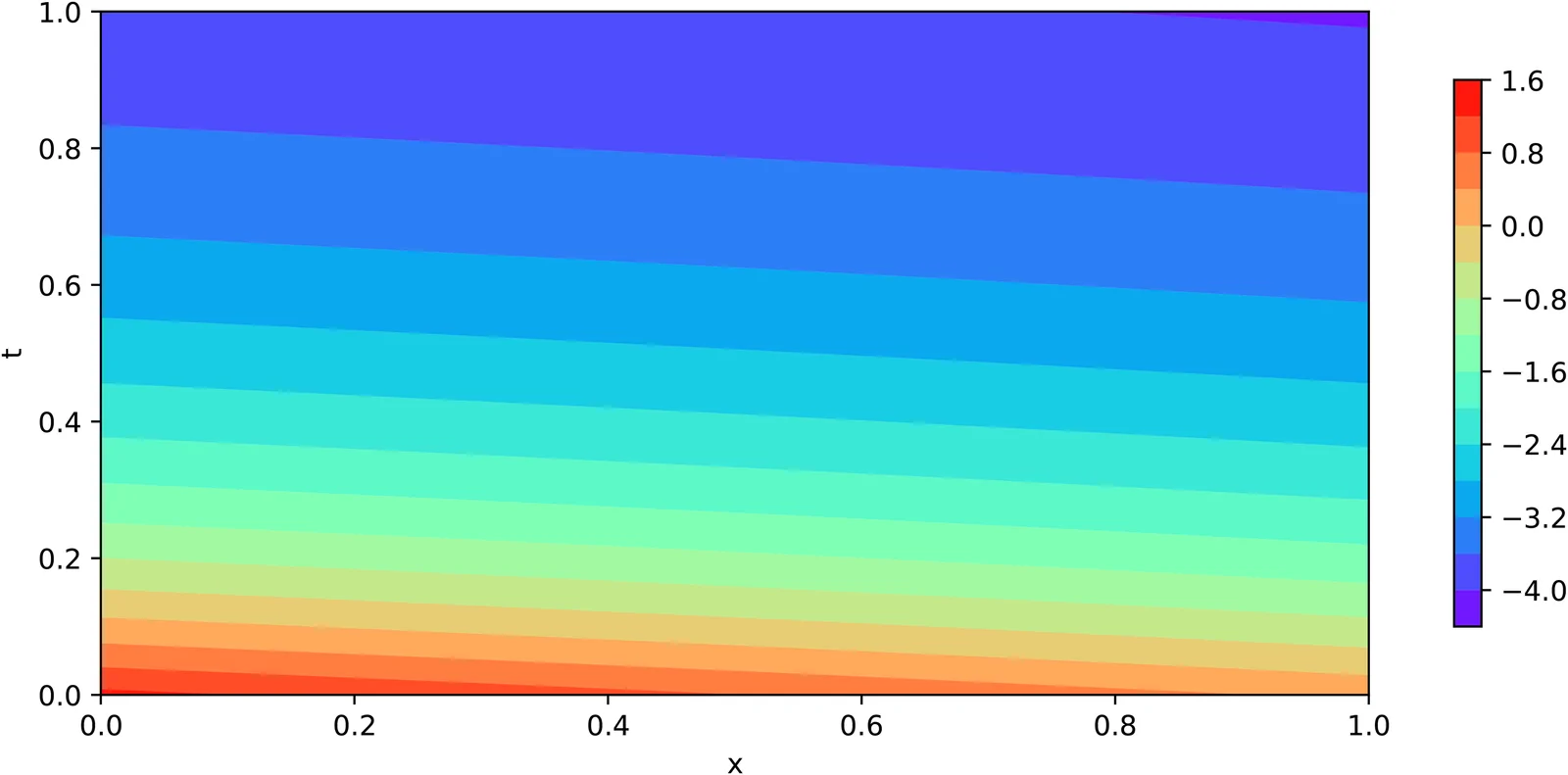
Contour solution comparison for Case III. S-PINN contour.

**Fig 44:**
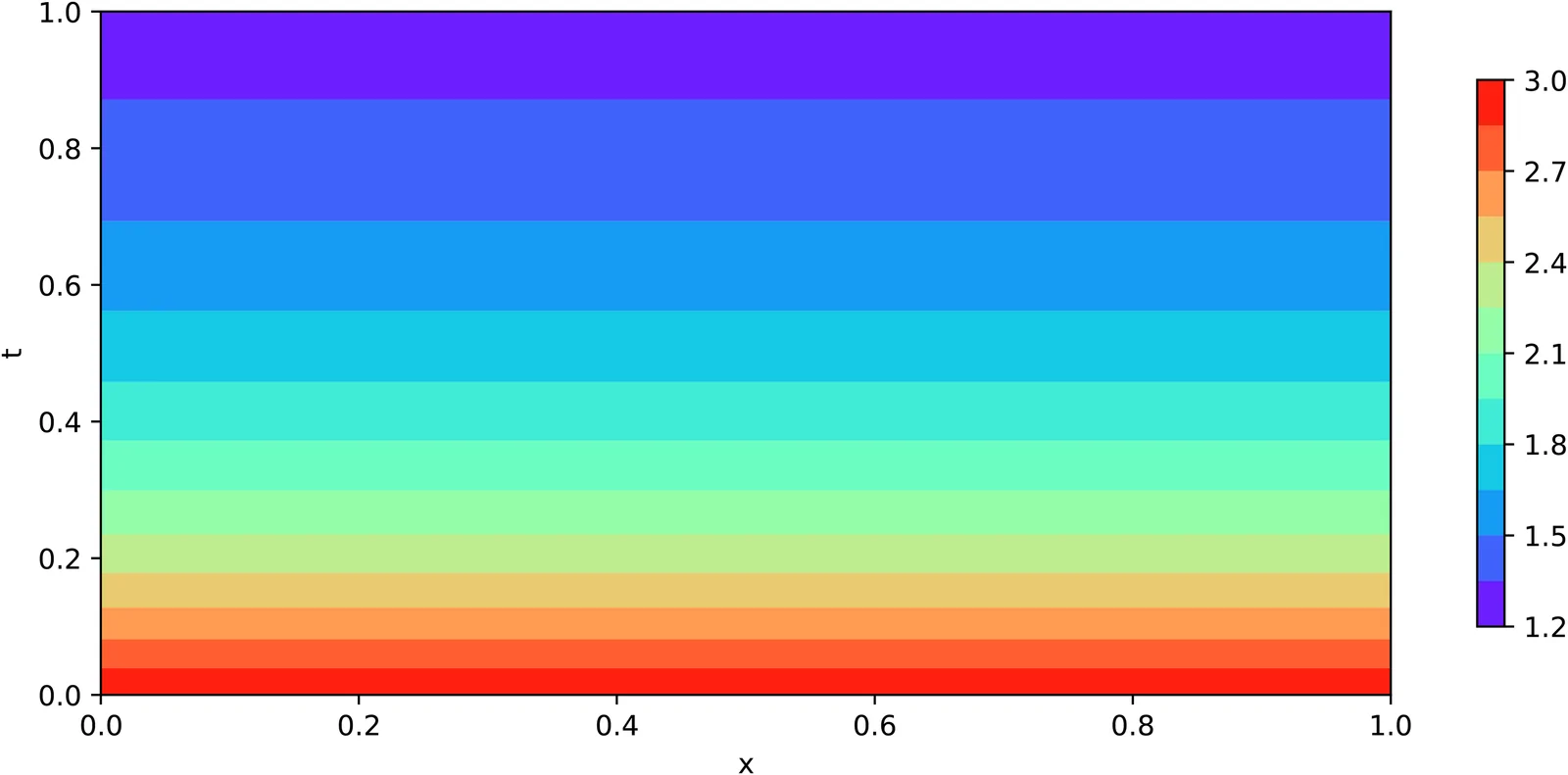
Contour solution comparison for Case III. PINN contour.

The exact surface in Fig 39 shows a smooth and coherent pattern. The solution changes continuously over the domain without artificial jumps or irregular ridges. The corresponding exact contour plot in Fig 42 shows well-organized contour lines, which represent the natural structure of the analytical solution.

The S-PINN surface in Fig 40 closely follows the structure of the exact surface. It preserves the smooth variation of the solution and captures the global shape more accurately than the standard PINN. The S-PINN contour plot in Fig 43 also shows smoother and more regularly spaced contour lines. This indicates that the proposed model preserves the geometric pattern of the solution, not only its pointwise magnitude.

In comparison, the standard PINN surface in Fig 41 shows the general trend but with weaker structural agreement. Some local irregularities and less accurate curvature representation can be observed. These differences become more visible in the contour plot shown in Fig 44, where the contour lines are less regular and less aligned with the exact pattern. This behavior suggests that the standard PINN approximates the solution but does not fully preserve the hidden symmetry structure of the reduced problem.

These results agree with recent symmetry-preserving PINN studies, where symmetry constraints are used as additional inductive biases to improve physical consistency and generalization. Compared with previous residual-only PINN models, the present S-PINN introduces symmetry information directly into the training process. Therefore, the network is encouraged to learn a solution that satisfies both the differential equation and the invariant structure. This explains the improved surface smoothness and contour regularity observed in Figs 39–41 and 42–44.

#### 5.2.12 Loss Components.

Figs 45 and 46 shows how the different loss components evolve during S-PINN training. This analysis is useful because the total loss alone cannot explain whether the network is learning all physical constraints in a balanced way. In PINN-type models, the total objective usually contains several competing terms, such as PDE residual losses, boundary losses, initial losses, and additional physical constraints. If one term dominates, the model may reduce the total loss while still producing an inaccurate or physically inconsistent solution.

**Fig 45:**
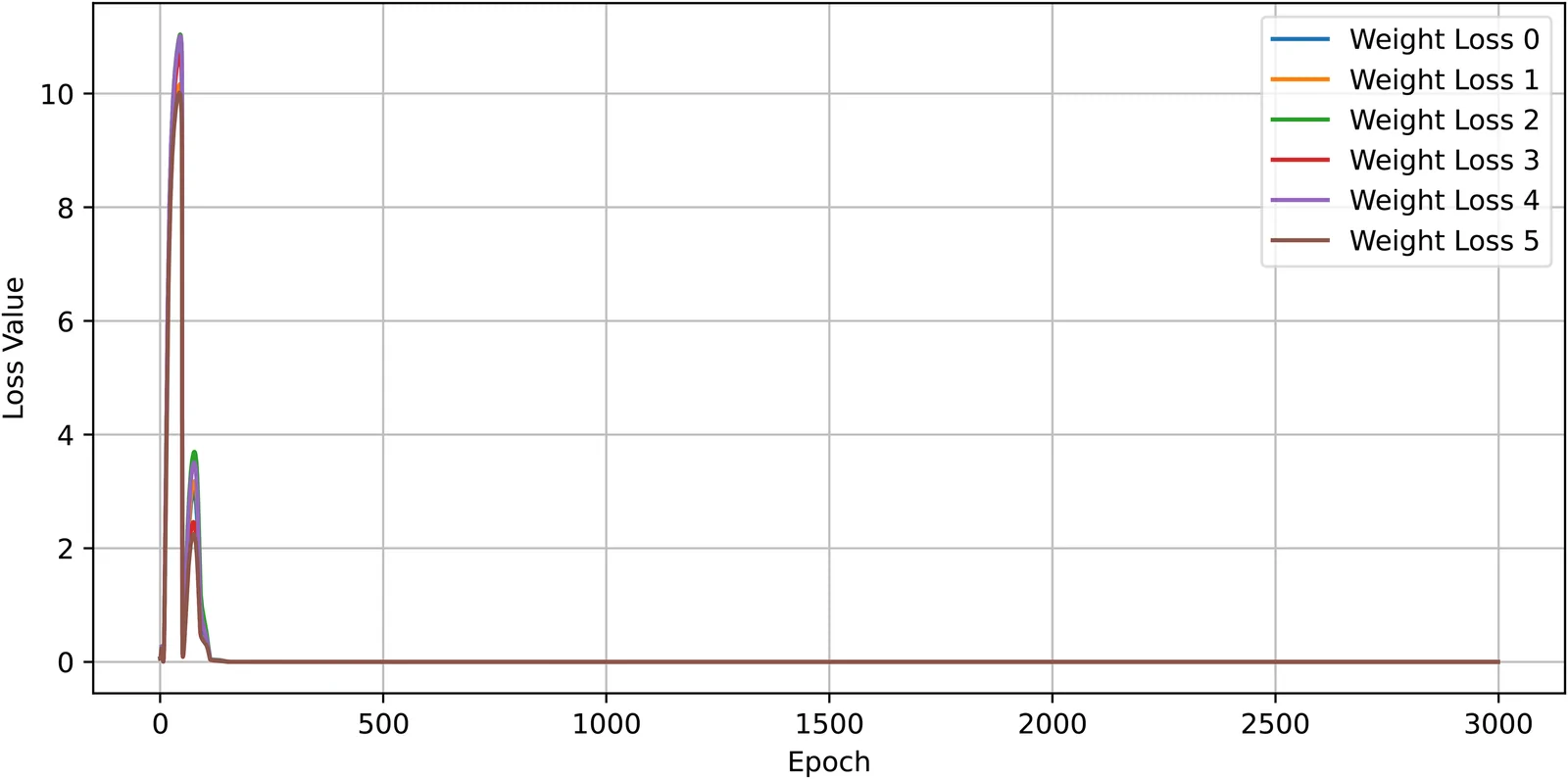
Loss component evolution for the S-PINN model in Case III. PDE loss components.

**Fig 46:**
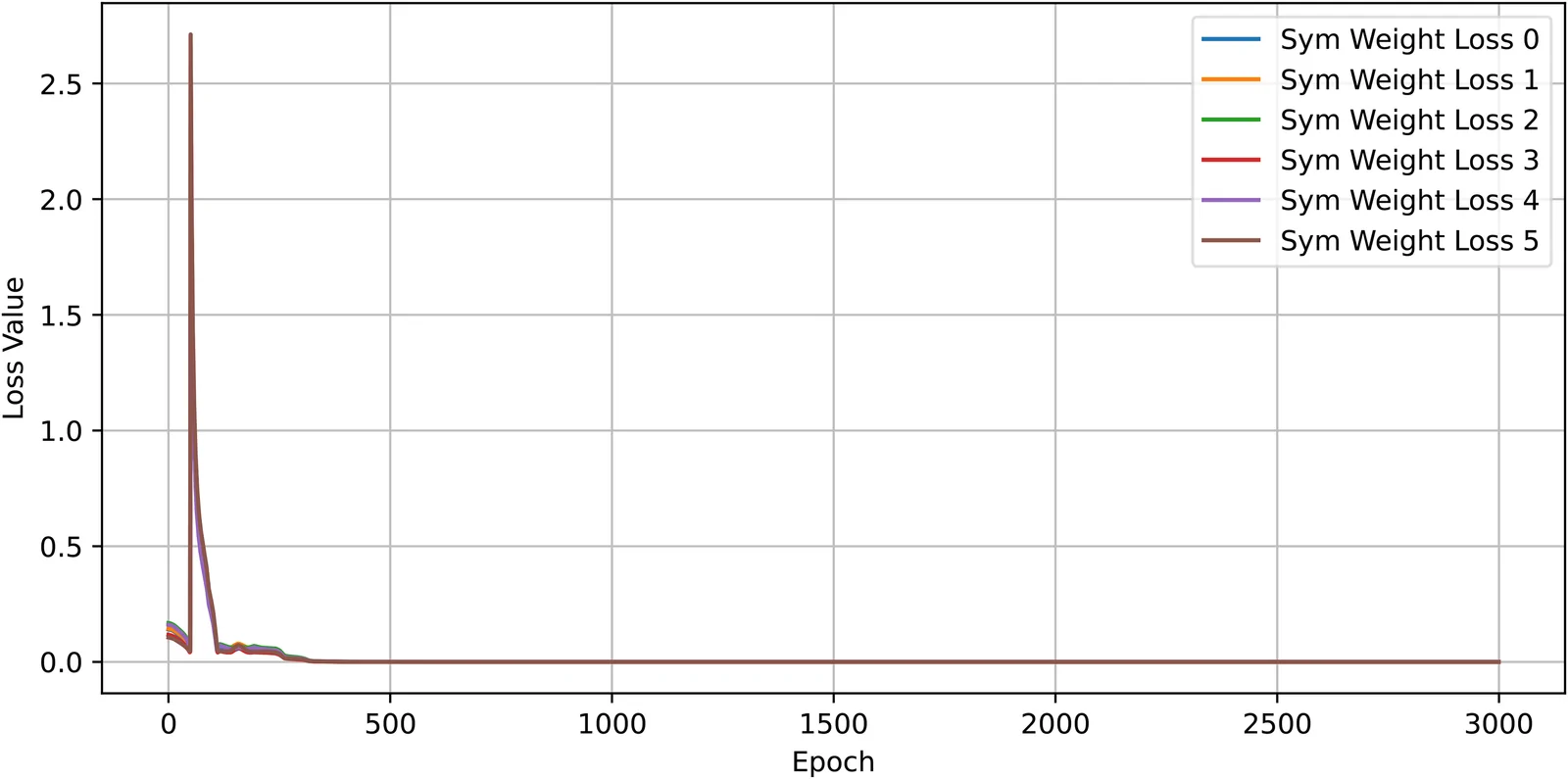
Loss component evolution for the S-PINN model in Case III. Symmetry loss components.

The PDE loss components in Fig 45 decrease steadily during training. This indicates that the S-PINN learns the governing equation in a balanced manner. No single residual component dominates the training process for a long time, which suggests that the reduced system is being learned consistently. This behavior supports the accuracy shown earlier in the ODE, surface, and contour comparisons.

The symmetry loss components in Fig 46 also show a stable decreasing trend. At the early stage of training, the network quickly captures the main symmetry pattern. After that, the symmetry loss decreases more gradually, which indicates a refinement stage where the model improves the accuracy of symmetry enforcement. This two-step behavior is physically meaningful: first, the model learns the dominant invariant structure, and then it adjusts the solution to satisfy the PDE and symmetry constraints simultaneously.

This result directly addresses a common problem in standard PINNs. Previous studies showed that PINNs can suffer from gradient pathologies when different loss terms create unbalanced gradients during back-propagation. Similarly, NTK-based studies showed that different components of the PINN loss may converge at different rates. In the present S-PINN, the balanced decay of PDE and symmetry losses indicates that the added symmetry constraint improves the conditioning of the training process. Thus, the proposed model improves on previous PINN formulations by producing a more organized and physically guided optimization path.

#### 5.2.13 Weight evolution.

Figs 47 and 48 illustrates the evolution of the trainable weights during S-PINN training. The PDE network weights in Fig 47 gradually move toward a stable regime. This indicates that the network learns the governing dynamics without strong oscillatory behavior. A stable weight pattern is an important sign of healthy optimization because it means that the model is not making large and inconsistent parameter corrections throughout training.

**Fig 47:**
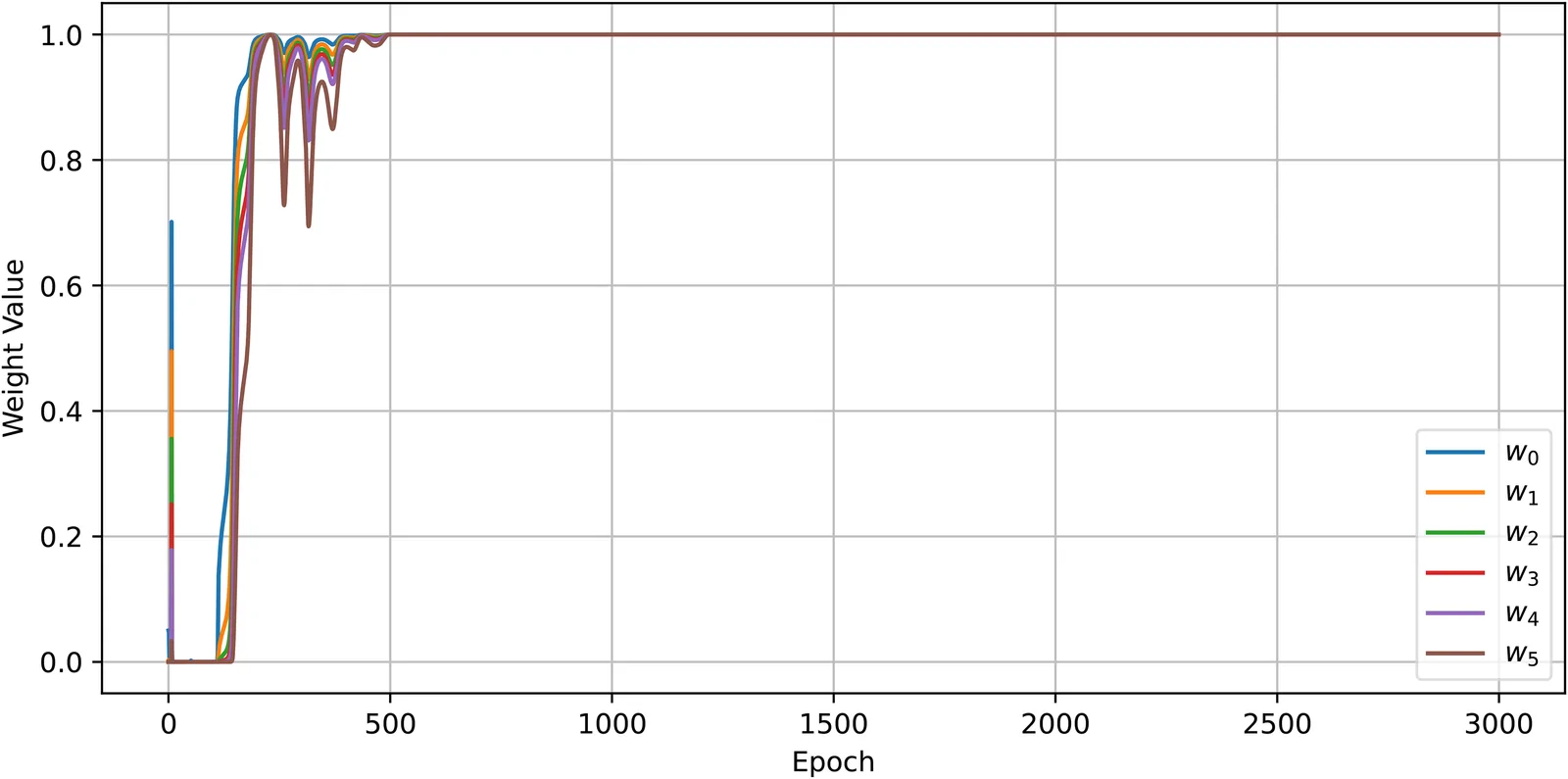
Weight evolution during S-PINN training for Case III. PDE network weights.

**Fig 48:**
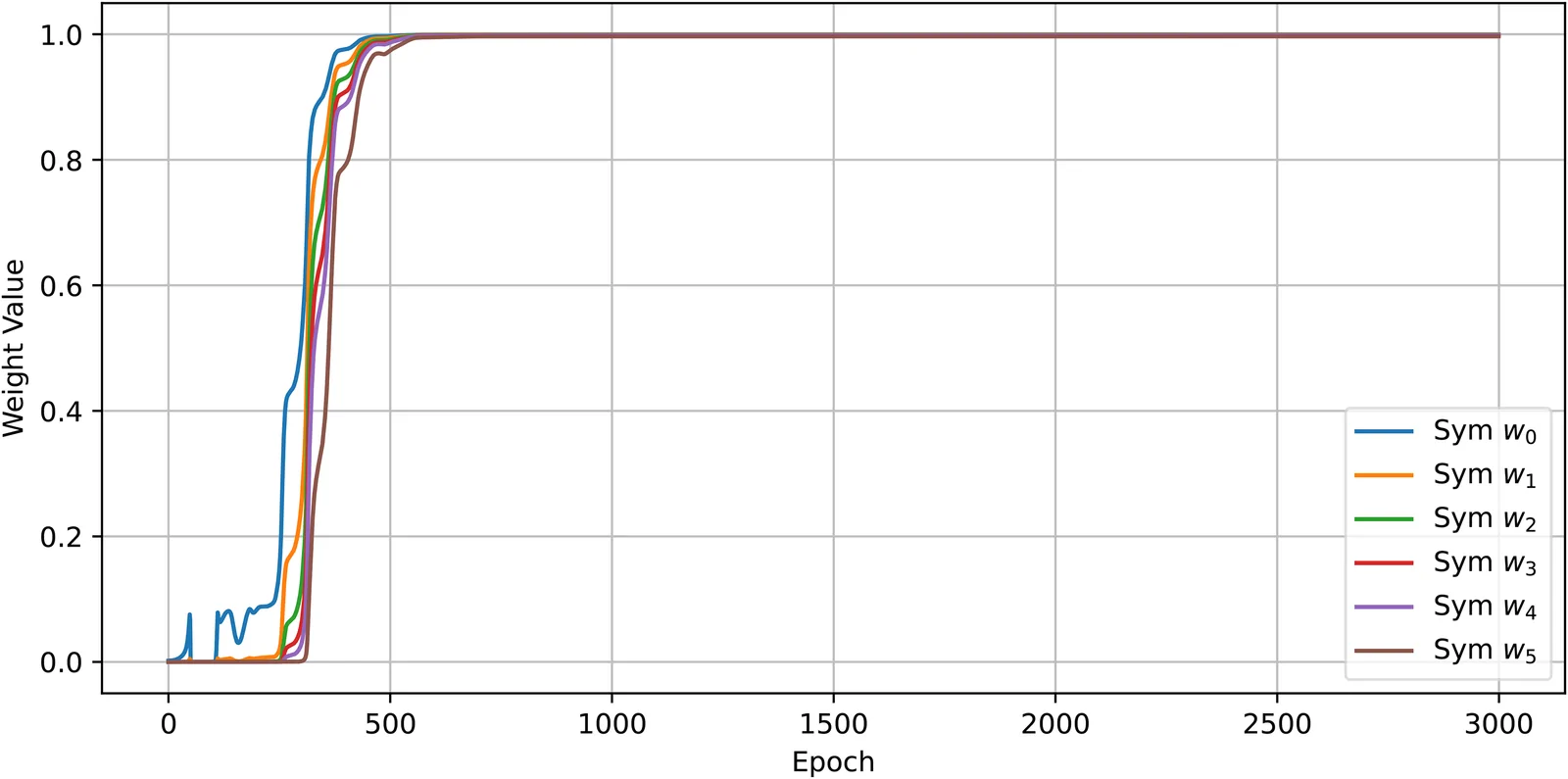
Weight evolution during S-PINN training for Case III. Symmetry network weights.

The symmetry network weights in Fig 48 show coordinated adaptation during the early training stage and then become more stable. This behavior suggests that the symmetry component is learned early and then acts as a regularizing mechanism for the remaining optimization process. Once the network understands the invariant structure, the later updates become smaller and more controlled.

The weight evolution results explain why the S-PINN performs better than the standard PINN in the solution plots. The symmetry weights help guide the learning process toward a structurally meaningful solution. This is consistent with Lie-symmetry-informed PINN studies, where symmetry losses are shown to improve sample efficiency and help the model learn neighboring symmetry-generated solutions. Therefore, the proposed S-PINN improves not only the final solution accuracy but also the learning dynamics of the neural network.

#### 5.2.14 Overall discussion for case III.

The complete comparison for Case III shows that the S-PINN provides clear advantages over the standard PINN. The ODE solution comparison confirms closer agreement with the exact solution. The surface and contour plots show that the S-PINN better preserves the global geometry of the analytical solution. The loss-component analysis shows that the PDE and symmetry losses decrease in a balanced way, while the weight evolution confirms that the training process becomes stable after the early adjustment stage.

Compared with classical PINNs, the proposed S-PINN does not rely only on residual minimization. Instead, it incorporates symmetry information into the learning process. This additional structure reduces unnecessary exploration of the solution space and guides the model toward physically admissible solutions. Compared with previous PINN improvements that mainly address optimization or loss balancing, the present method improves the model at the mathematical-structure level. Therefore, the S-PINN gives a more accurate, stable, and physically consistent approximation for Case III.

### 5.3 Case IV

This section presents a detailed comparison between the standard Physics-Informed Neural Network and the proposed symmetry-preserving S-PINN for Case IV. The comparison focuses on loss convergence, computational update cost, and gradient behavior. These three indicators are important because they show not only whether the model reaches a good final solution, but also how stable and efficient the training process is.

#### 5.3.1 Loss convergence.

Figs 49 and 50 compares the total loss convergence histories of the S-PINN and the standard PINN. The S-PINN loss decreases more smoothly and reaches a stable region earlier than the standard PINN. This indicates that the symmetry-preserving formulation improves the optimization path and helps the model approach the physically relevant solution space more directly.

**Fig 49:**
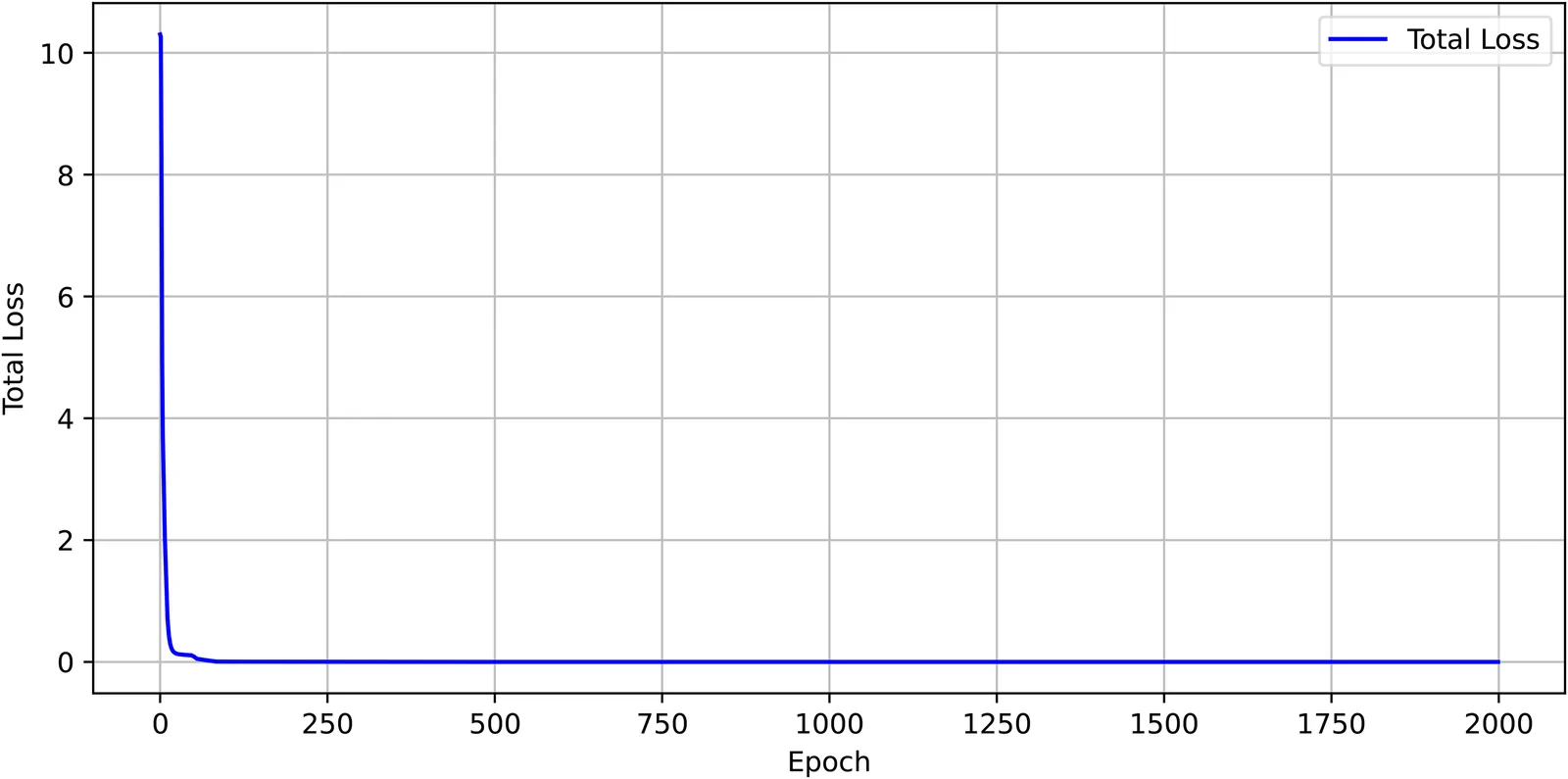
Total loss convergence comparison for Case IV. S-PINN total loss.

**Fig 50:**
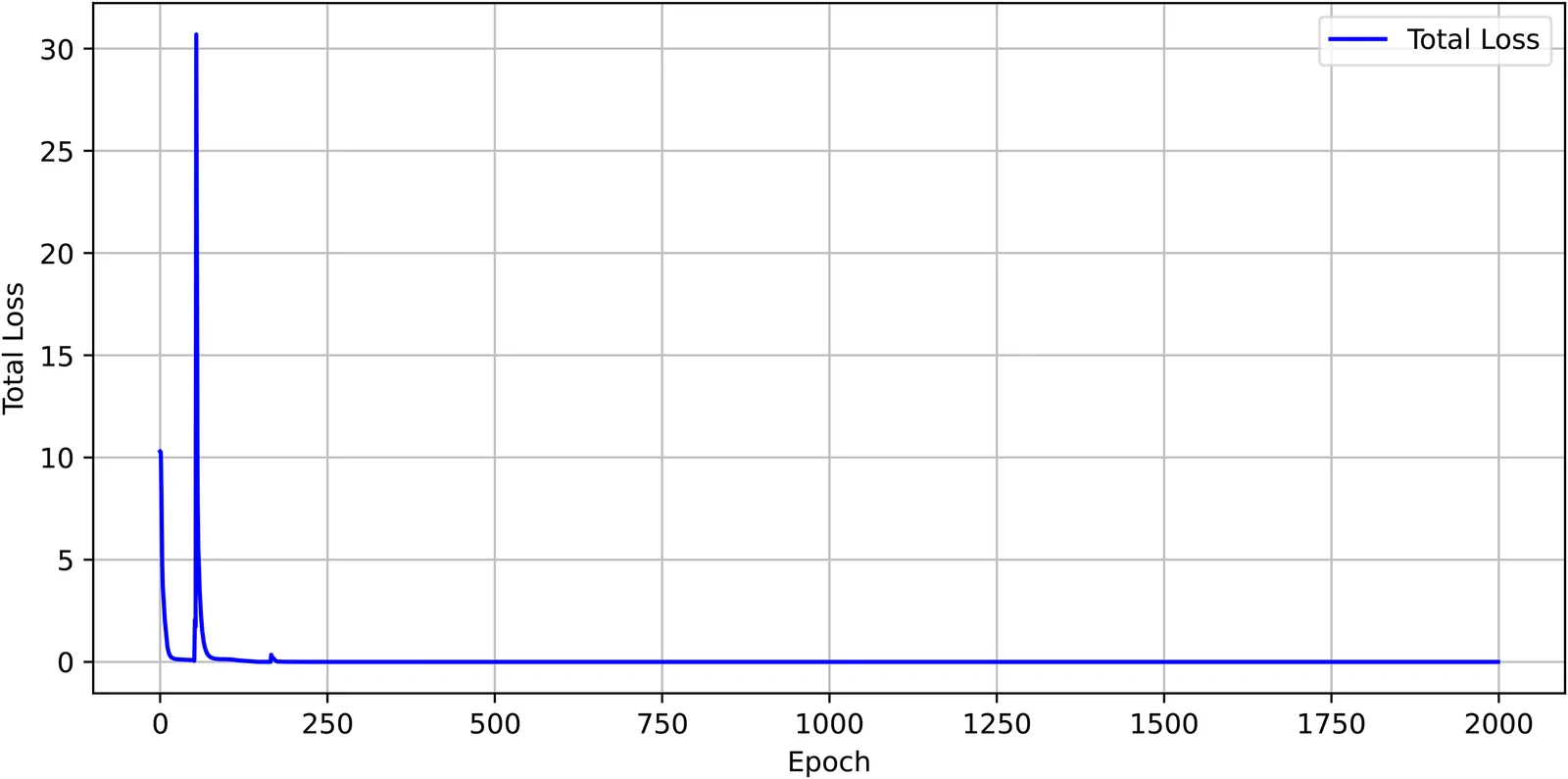
Total loss convergence comparison for Case IV. PINN total loss.

The standard PINN also reduces the loss, but its convergence curve shows more visible fluctuations during training. These oscillations suggest that the optimizer has to work harder to balance the different parts of the loss function. Since the standard PINN does not explicitly include the symmetry structure, it must learn the physical solution mainly through residual minimization. This makes the training process more sensitive to the loss landscape and may result in slower stabilization.

The improved convergence of the S-PINN agrees with earlier findings in the PINN literature. Classical PINNs are powerful because they embed differential equations into the training loss; however, later studies showed that their training can become difficult when the composite loss terms are not balanced properly. In the present case, the symmetry constraint acts as a structural guide. It reduces the effective search space and encourages the network to learn solutions that are already compatible with the mathematical invariance of the problem. Therefore, Figs 49 and 50 shows that the proposed S-PINN improves the convergence behavior compared with the standard PINN.

#### 5.3.2 Computational efficiency.

Figs 51 and 52 shows the per-epoch update cost for both models. The update cost reflects the computational effort required by the optimizer to adjust the trainable parameters at each epoch. In the S-PINN model, the update cost may initially be higher because the training process includes both the PDE residual and the symmetry-enforcement term. This extra cost is expected because the model is learning not only the solution but also the invariant structure of the reduced problem.

**Fig 51:**
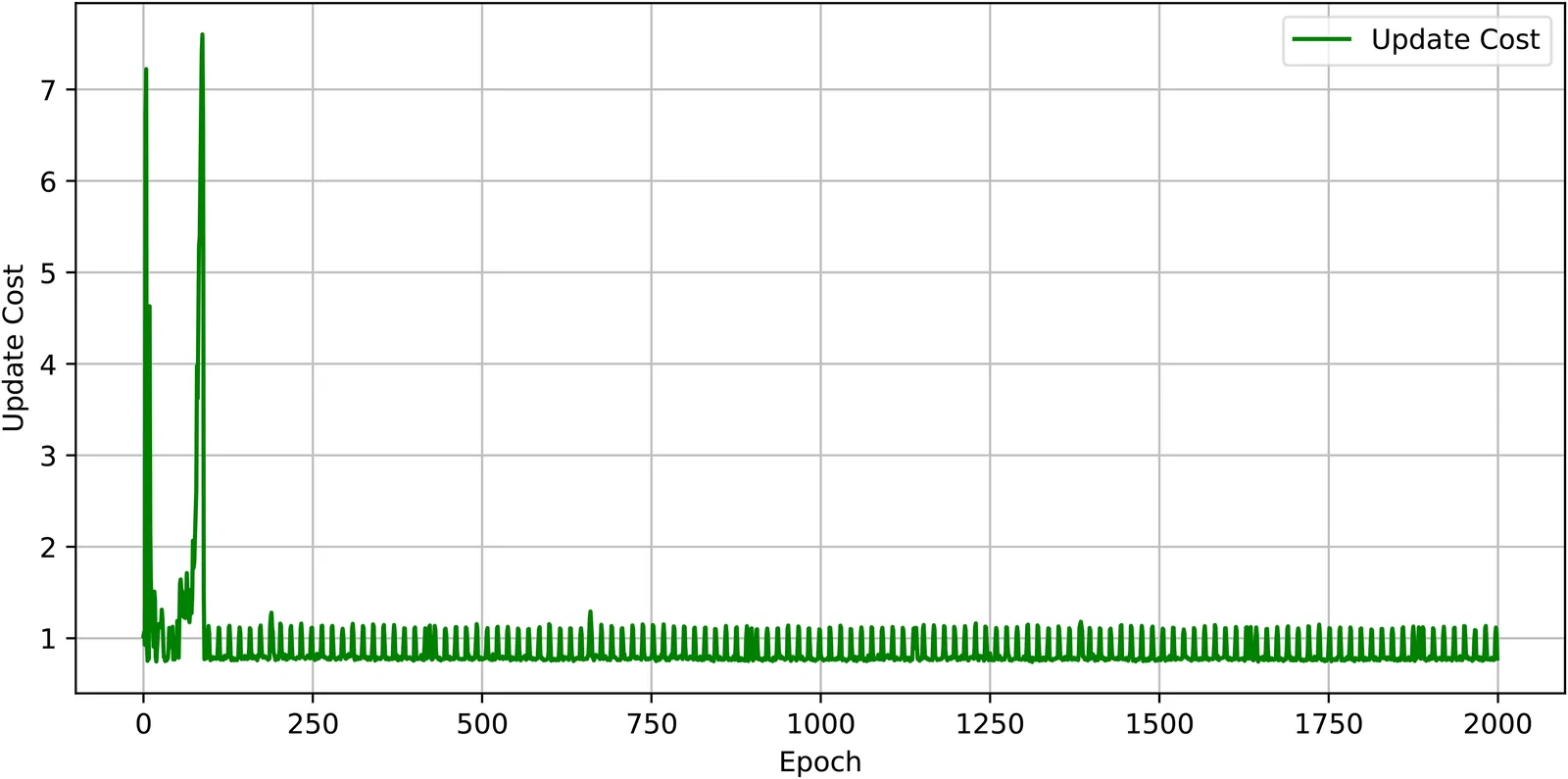
Update cost comparison during training for Case IV. S-PINN update cost.

**Fig 52:**
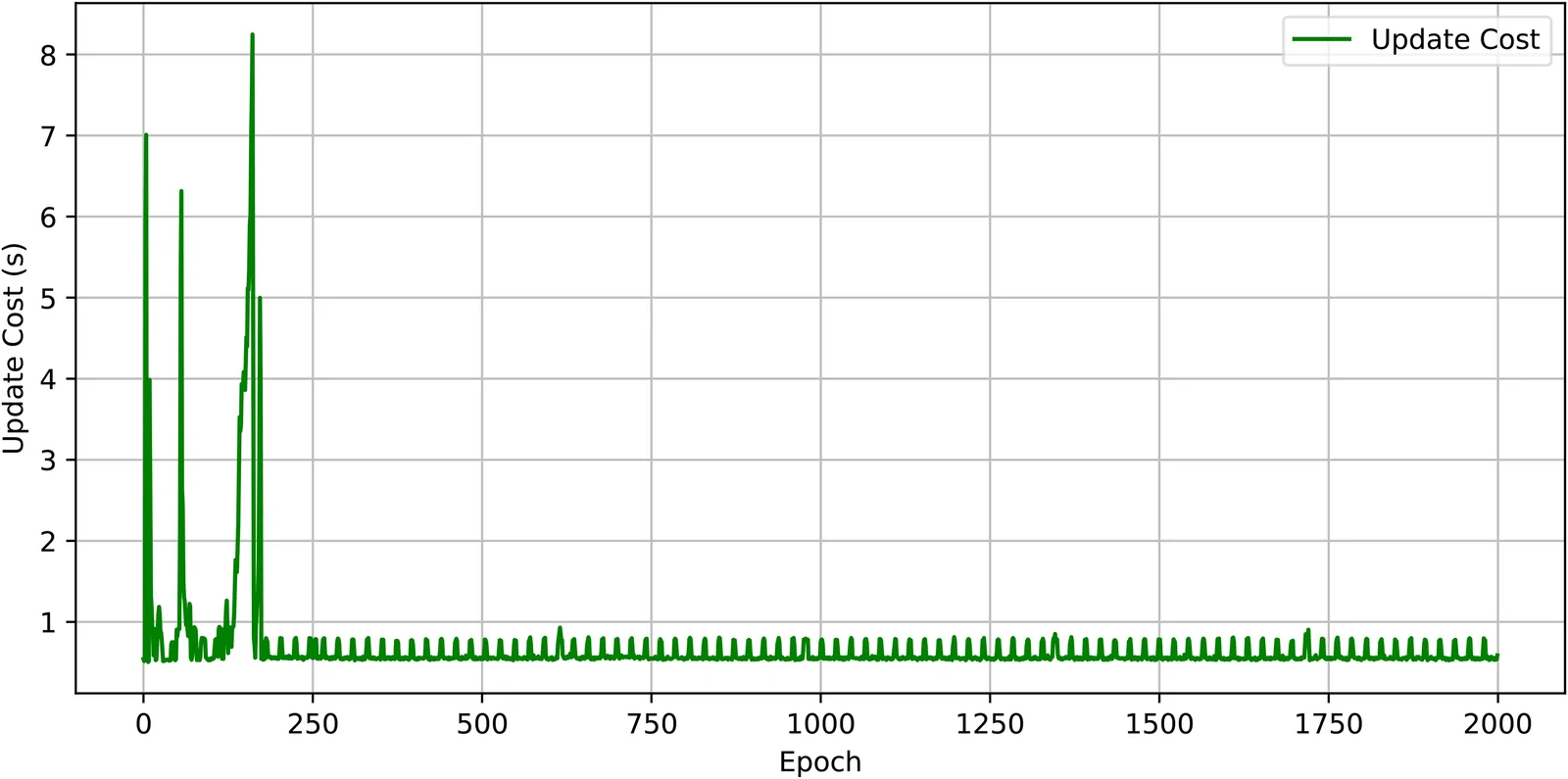
Update cost comparison during training for Case IV. PINN update cost.

However, the important point is that this additional cost is compensated by better training stability and faster effective convergence. After the early training stage, the S-PINN update cost becomes more regular, indicating that the optimizer enters a more stable regime. The standard PINN shows a comparatively less organized update pattern, which suggests that the optimizer continues to make less uniform corrections during training.

This result shows that computational efficiency should not be judged only by the cost of one epoch. A method that requires slightly more work per epoch can still be more efficient overall if it converges faster and produces a more reliable solution. This is particularly important in scientific machine learning, where models are often used for repeated simulations, inverse problems, and parametric studies. The present S-PINN improves on previous residual-only PINNs by using symmetry information to make each training direction more physically meaningful. This agrees with recent structure-preserving PINN studies, where adding symmetry constraints improves generalization and reduces unnecessary exploration of the solution space.

#### 5.3.3 Gradient behavior.

Figs 53 and 54 compares the gradient norm evolution for the S-PINN and the standard PINN. The gradient norm is an important measure of training stability because it indicates the size of the parameter updates suggested by the loss function. Large or highly fluctuating gradient norms usually indicate stiffness, poor conditioning, or conflict between different loss terms.

**Fig 53:**
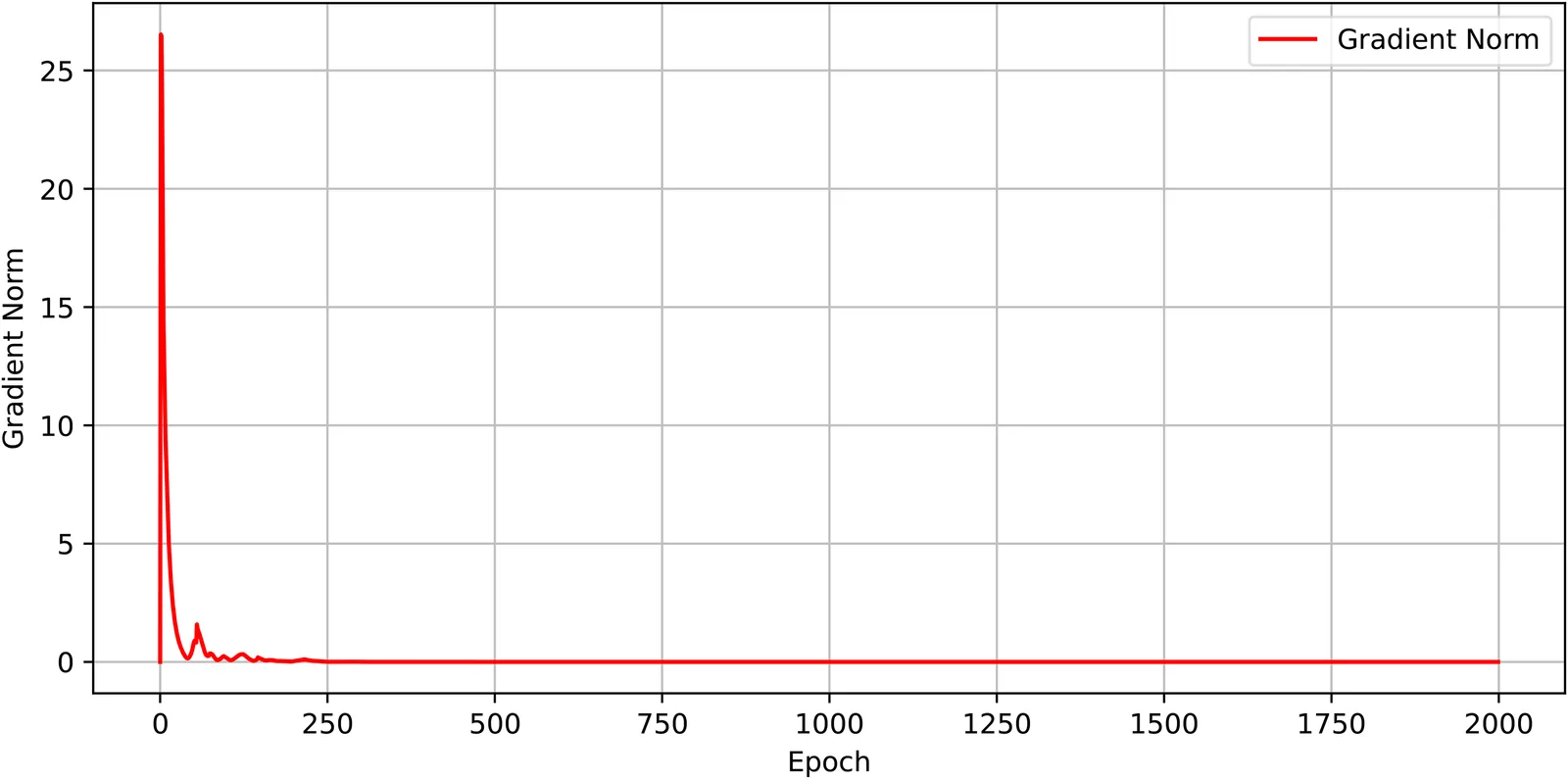
Gradient norm evolution during training for Case IV. S-PINN gradient norm.

**Fig 54:**
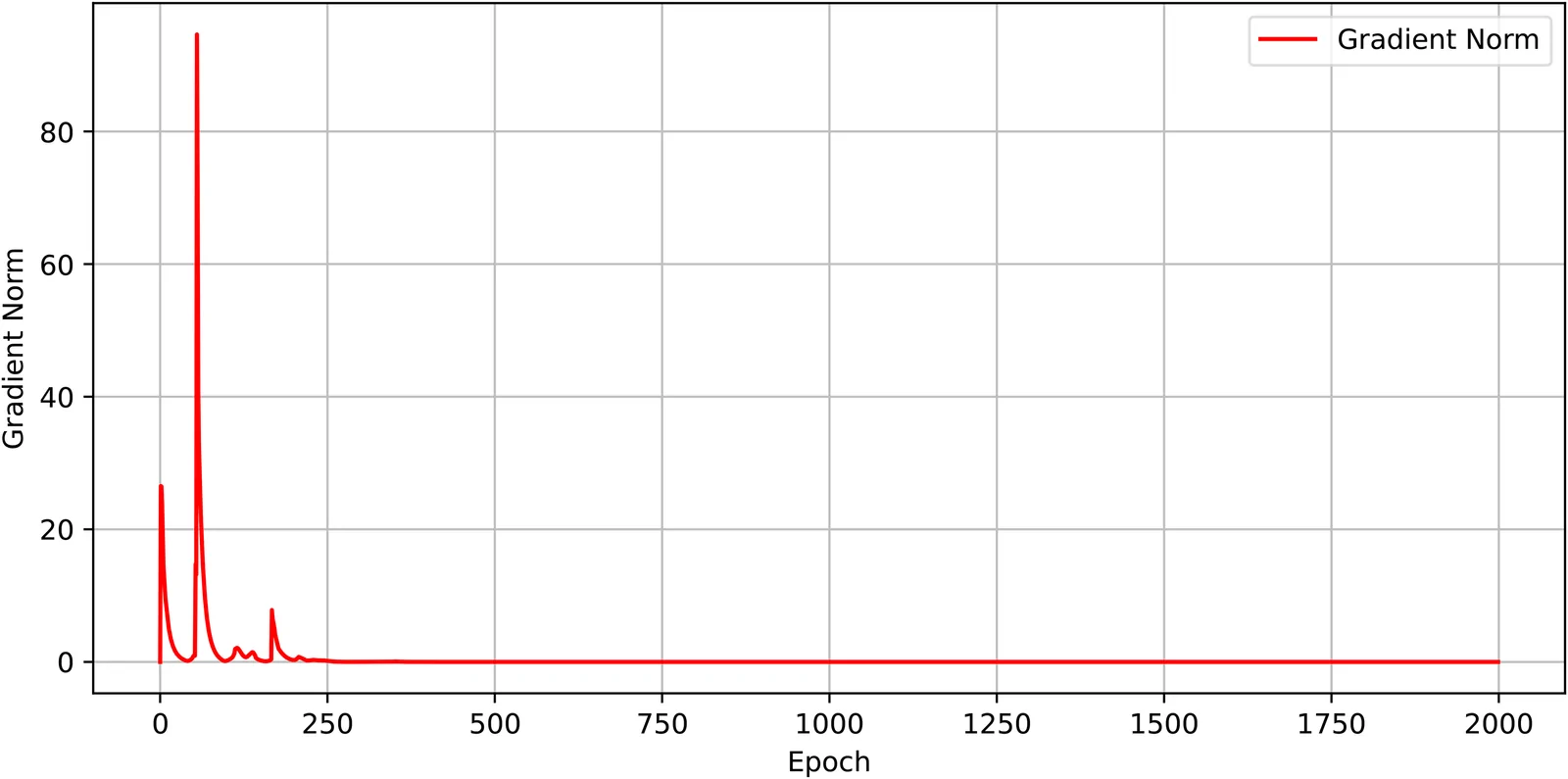
Gradient norm evolution during training for Case IV. PINN gradient norm.

The S-PINN gradient norm remains more stable during training. This means that the symmetry-preserving model avoids extreme parameter corrections and follows a smoother optimization path. The stable gradient behavior also explains the smoother loss convergence observed in Figs 49 and 50. Since the symmetry term guides the model toward an invariant solution space, the optimizer does not need to make large and unstable updates to satisfy the physical constraints.

In contrast, the standard PINN shows stronger gradient fluctuations. This behavior is consistent with the gradient-pathology problem reported in previous PINN studies. In standard PINNs, different loss components may generate gradients with different magnitudes and directions. This imbalance can slow convergence and reduce the quality of the final solution. The S-PINN reduces this difficulty by adding symmetry information, which acts as a regularizer and improves the conditioning of the optimization problem.

Therefore, the gradient comparison confirms that the proposed S-PINN improves the internal training dynamics of the model. It provides smoother gradients, better optimization stability, and stronger physical consistency than the standard PINN. This is one of the main reasons why the S-PINN gives better performance in Case IV.

#### 5.3.4 Overall discussion for case IV.

The results for Case IV show that the proposed S-PINN improves the standard PINN framework in terms of convergence, computational reliability, and gradient stability. The total loss curves show that the S-PINN reaches a stable low-loss region more smoothly. The update-cost comparison shows that the additional symmetry enforcement is worthwhile because it leads to more organized training behavior. The gradient norm comparison further confirms that the S-PINN reduces unstable parameter updates and improves the conditioning of the optimization problem.

Compared with earlier PINN models, the proposed S-PINN introduces a stronger physical and mathematical structure into the learning process. Compared with later works that focus mainly on improving training through adaptive weights or gradient-based corrections, the present approach improves the model by embedding symmetry directly into the loss formulation. This makes the learned solution more physically consistent and the training process more reliable.

Overall, Case IV confirms that symmetry preservation is an effective strategy for improving PINN performance. The proposed S-PINN does not simply reduce numerical error; it also improves the way the neural network learns the solution. This makes the method more suitable for nonlinear reduced differential equations where preserving the underlying mathematical structure is essential for accuracy and stability.

Fig 55 presents the layer-wise gradient norm evolution during the S-PINN training process for seven network layers. The gradient norms are plotted on a logarithmic scale, which makes it possible to observe both the early-stage large updates and the later-stage small corrective updates. At the beginning of training, all layers show relatively large gradient magnitudes, indicating that the optimizer actively adjusts the network parameters to reduce the dominant residual and symmetry errors. The initial decrease in the gradient norm shows that the network rapidly moves away from the randomly initialized state toward a more physically admissible solution region. During the early and intermediate epochs, the gradient norms exhibit several oscillatory patterns. These fluctuations are mainly associated with the interaction among the reduced ODE residual loss, the symmetry constraint loss, and the imposed boundary or anchor conditions. In particular, the interval around epochs 350–600 shows a temporary increase in gradient activity for several layers. This indicates that the optimizer performs stronger parameter corrections in this region to rebalance the competing loss components. However, the gradients remain bounded and do not grow uncontrollably, confirming that no gradient explosion occurs during training. A layer-dependent behavior can also be observed. The first few layers generally maintain slightly larger gradient magnitudes during the active optimization phase, suggesting that these layers play an important role in learning the dominant features of the reduced similarity representation. The deeper layers show comparatively smaller and more refined gradient variations, especially after the intermediate training phase. This indicates that the deeper layers mainly contribute to fine-scale correction of the learned solution rather than large structural changes. Toward the final stage of training, the gradient norms decrease and remain mostly within the range of approximately 10^–3^ to 10^–2^, with small bounded oscillations. This behavior shows that the optimizer approaches a stable region of the loss landscape while still preserving sufficient gradient activity for final refinement. Overall, the layer-wise gradient norm evolution confirms that the proposed S-PINN framework provides stable gradient propagation across all layers and maintains a well-conditioned training process for the symmetry-preserving nonlinear model.

**Fig 55:**
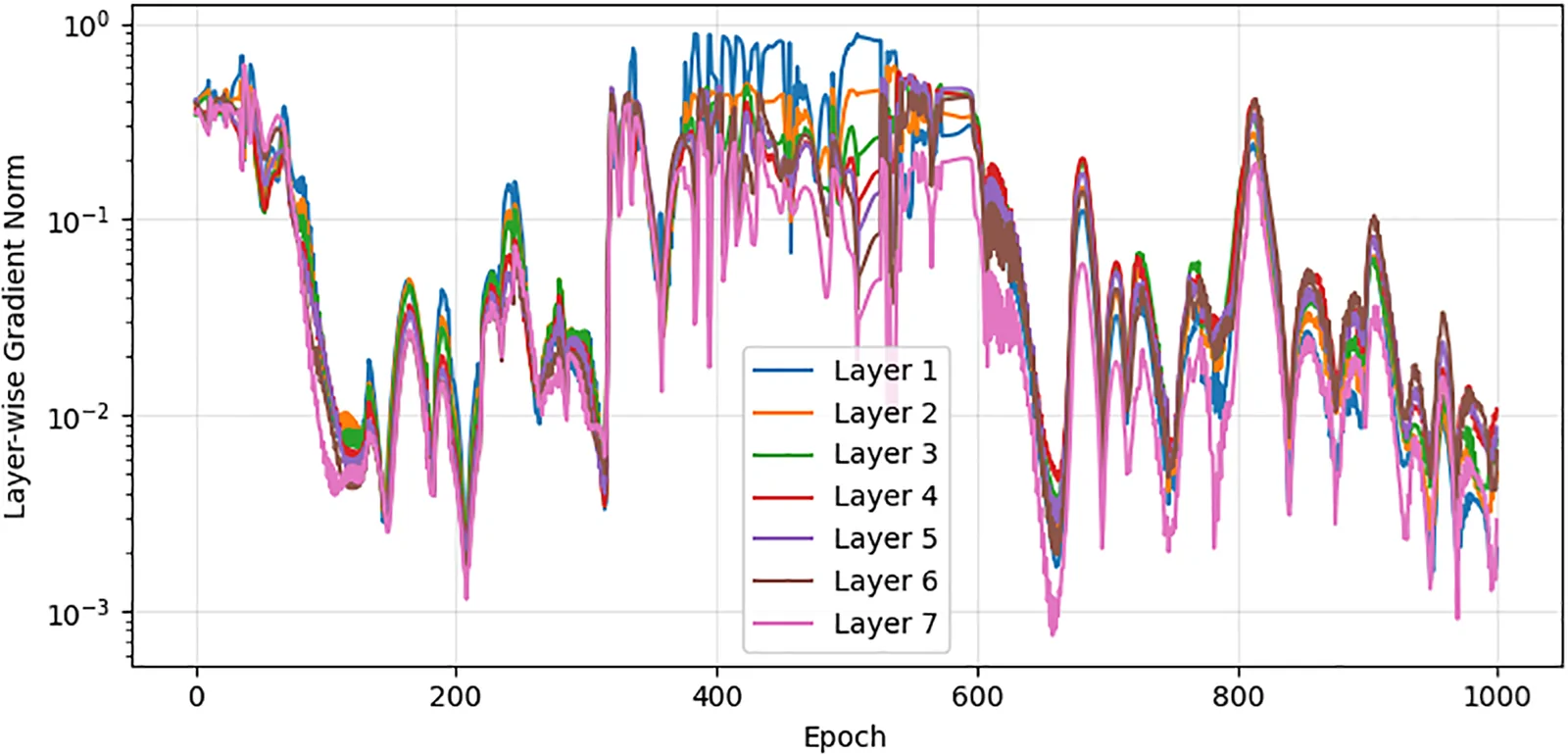
Layer-wise gradient norm evolution during S-PINN training.

#### 5.3.5 ODE solution comparison.

Fig 56 presents the direct comparison between the exact analytical solution, the standard PINN approximation, and the proposed S-PINN prediction for the reduced ODE problem in Case IV. This figure is important because it shows the actual solution quality after training, rather than only reporting the loss value. The S-PINN curve remains closer to the exact solution over the full computational interval, which confirms that the symmetry-enhanced formulation improves the ability of the neural network to recover the correct reduced solution profile.

**Fig 56:**
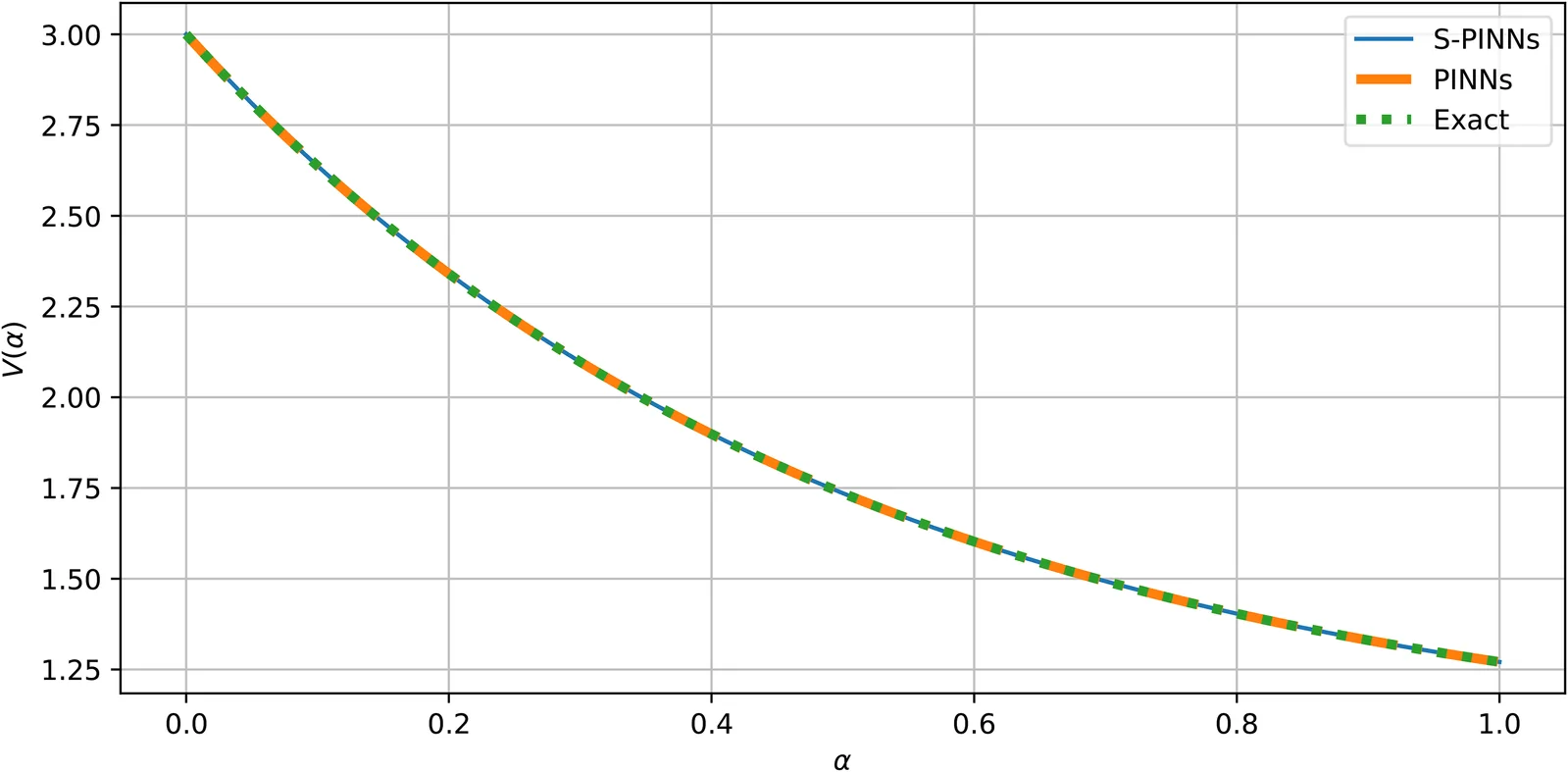
Comparison of reduced ODE solutions for Case IV. The exact analytical solution is compared with the standard PINN and the proposed S-PINN predictions.

The improvement becomes more meaningful in sensitive regions of the domain, where small errors in curvature or slope can lead to visible deviations from the exact solution. In these regions, the standard PINN captures the general trend, but it shows noticeable mismatch from the exact curve. This indicates that the standard residual-based training can approximate the solution, but it may not fully preserve the hidden structure of the reduced equation. The S-PINN, in contrast, benefits from the additional symmetry information, which acts as a mathematical guide during training. As a result, the learned solution remains more physically consistent and does not drift away from the analytical profile.

This behavior agrees with the known strengths and limitations of classical PINNs. The standard PINN framework embeds the governing equation into the loss function and has been widely used for solving nonlinear differential equations. However, later studies showed that standard PINNs may suffer from slow convergence, loss imbalance, and gradient pathologies, especially when the solution contains nonlinear structures or when different loss terms compete during training. The present S-PINN improves on this residual-only formulation by adding symmetry preservation as an additional constraint. Therefore, the network is not only minimizing the governing residual but also learning within a more physically admissible solution space.

Recent Lie-symmetry-informed neural solver studies have also shown that incorporating symmetry information can improve generalization, sample efficiency, and physical consistency. The present result supports this conclusion. The closer agreement of the S-PINN with the exact solution in Fig 56 shows that symmetry-aware modeling improves the accuracy of the reduced ODE solution and provides a more reliable alternative to the standard PINN.

#### 5.3.6 Surface and contour plots.

Figs 57–59 and 60–62 provide a more complete view of the learned solution in the full computational domain. While Fig 56 evaluates the reduced ODE profile, the surface and contour plots show whether the neural network can reconstruct the global solution field. This is a stronger test of model performance because a network may match the one-dimensional solution profile reasonably well but still fail to preserve the full geometric structure of the solution.

**Fig 57:**
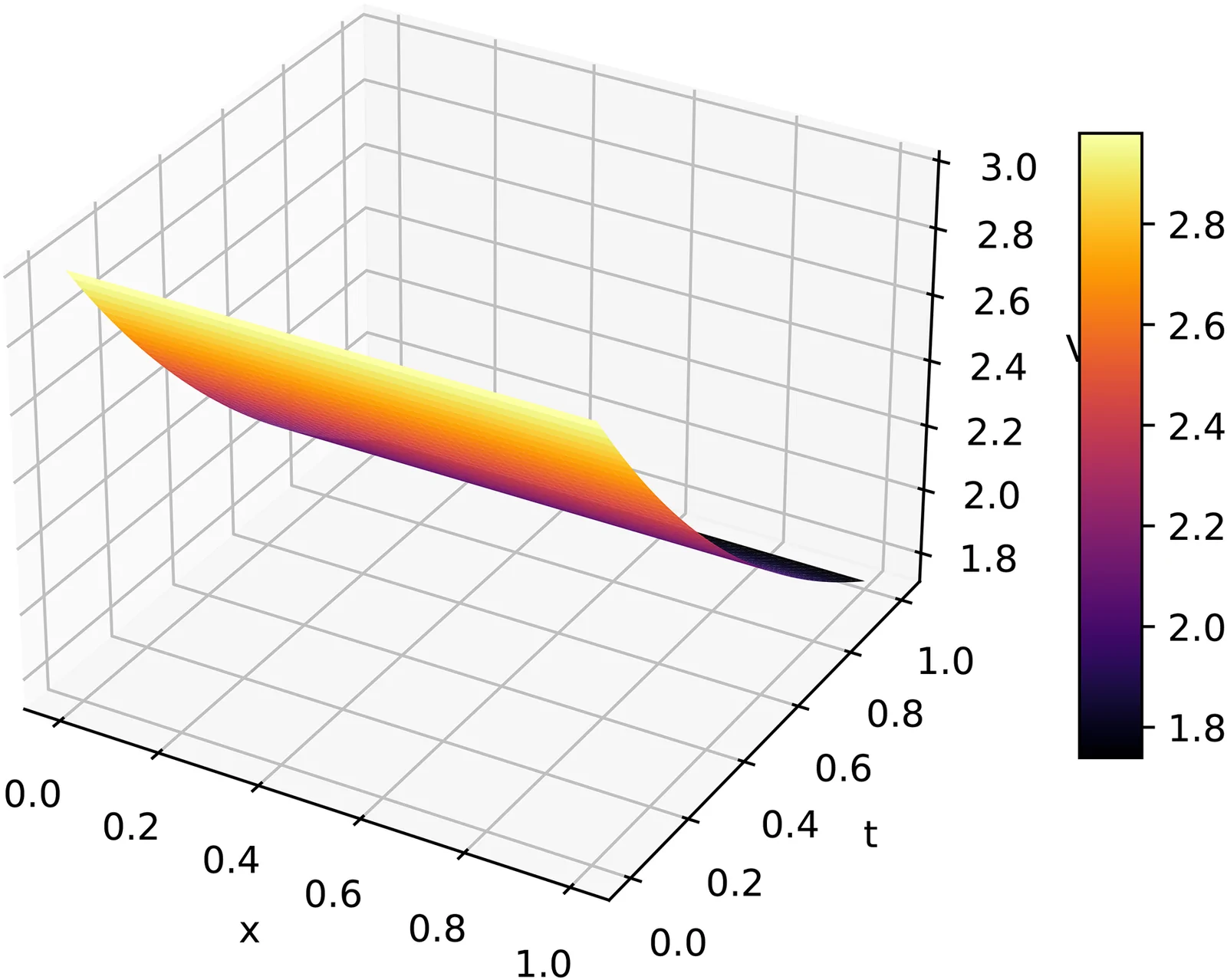
Surface solution comparison for Case IV. Exact surface.

**Fig 58:**
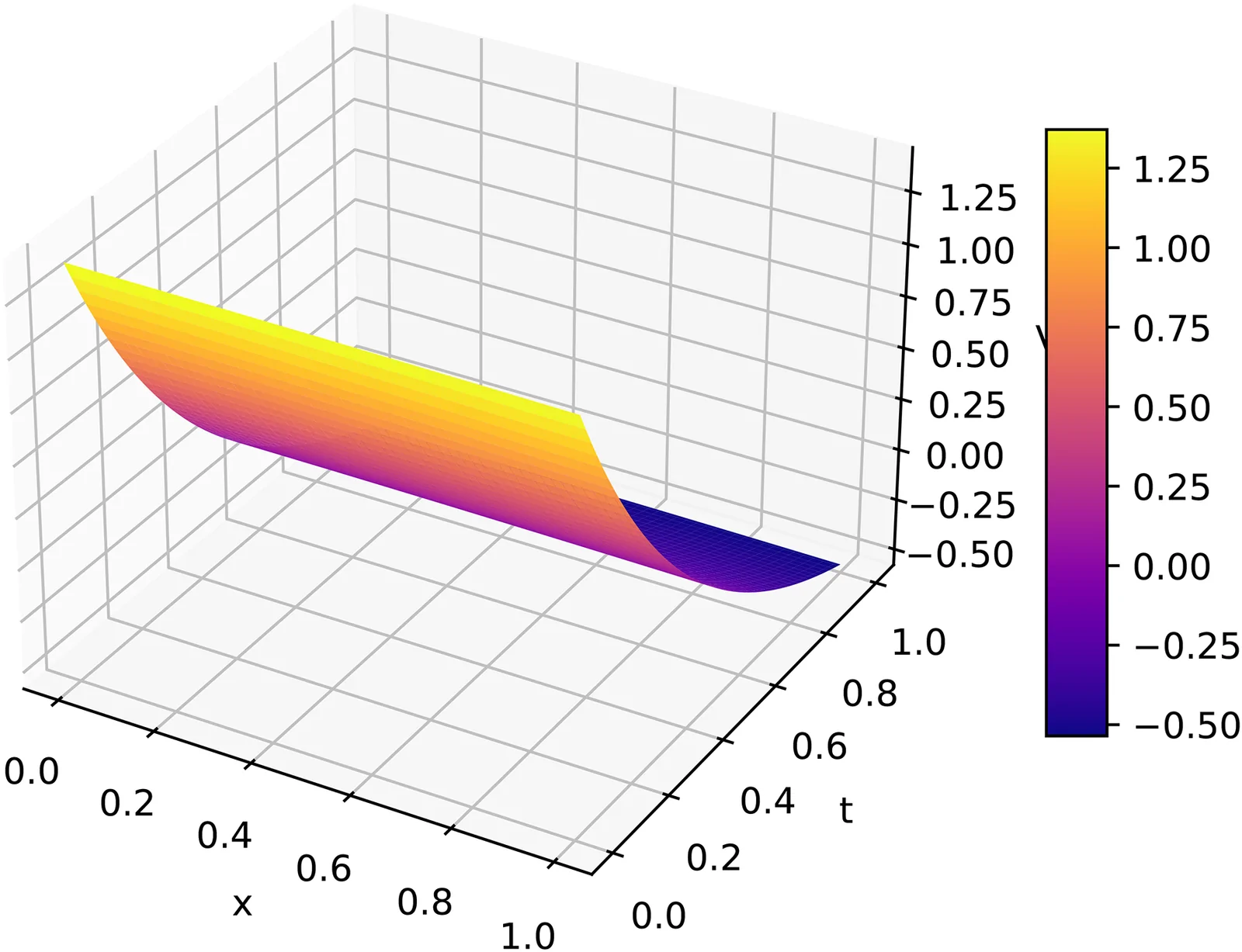
Surface solution comparison for Case IV. S-PINN surface.

**Fig 59:**
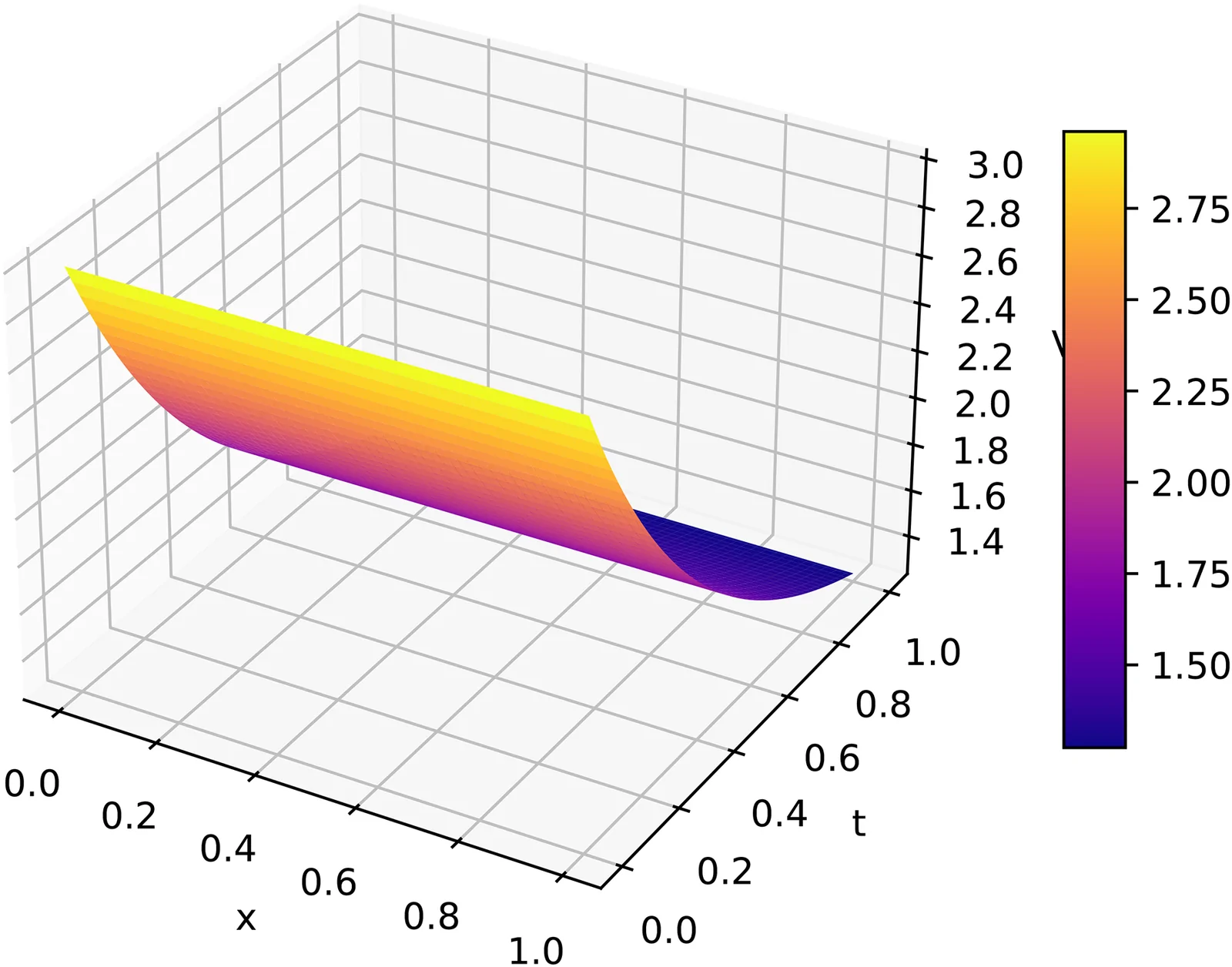
Surface solution comparison for Case IV. PINN surface.

**Fig 60:**
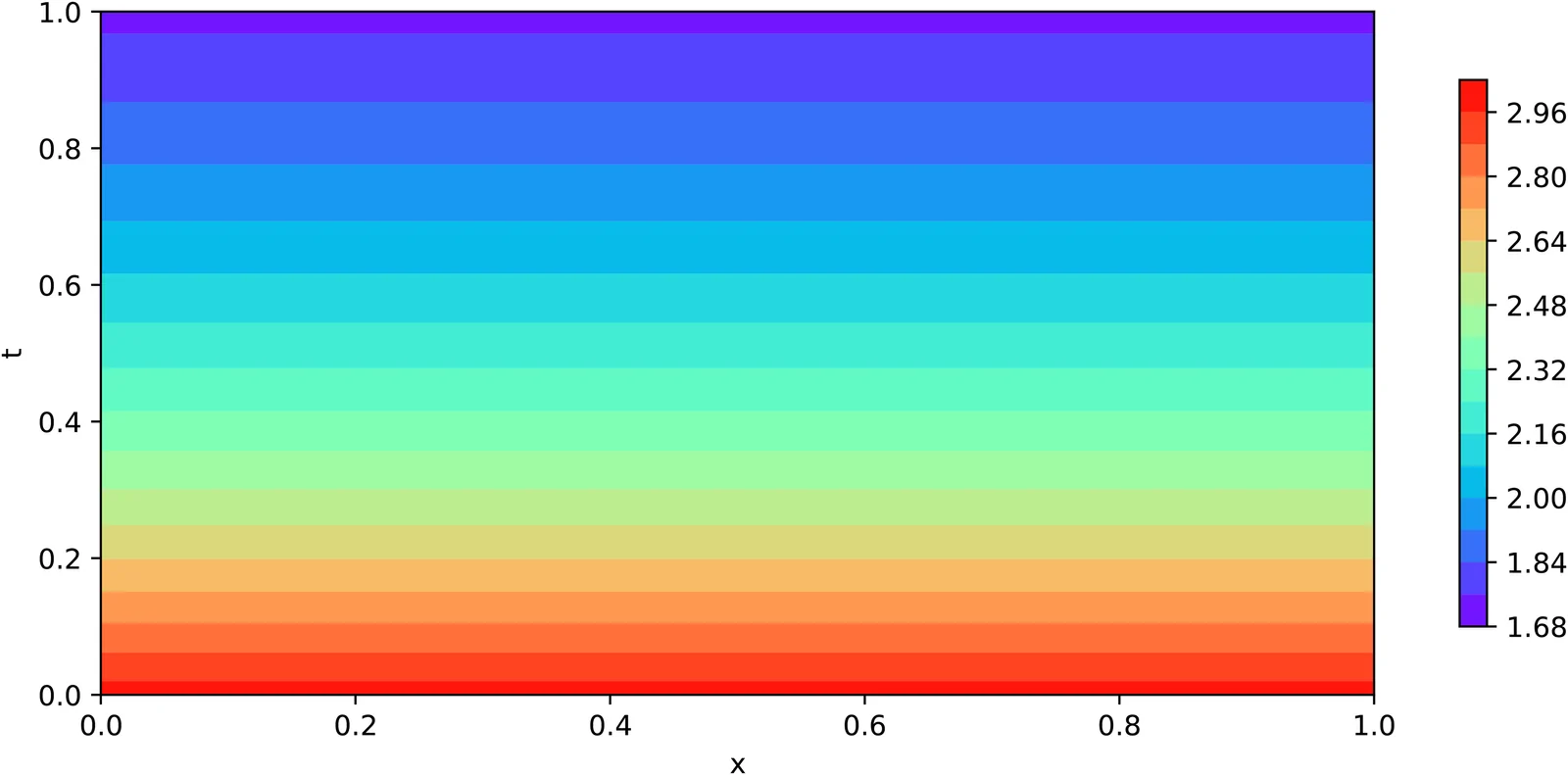
Contour solution comparison for Case IV. Exact contour.

**Fig 61:**
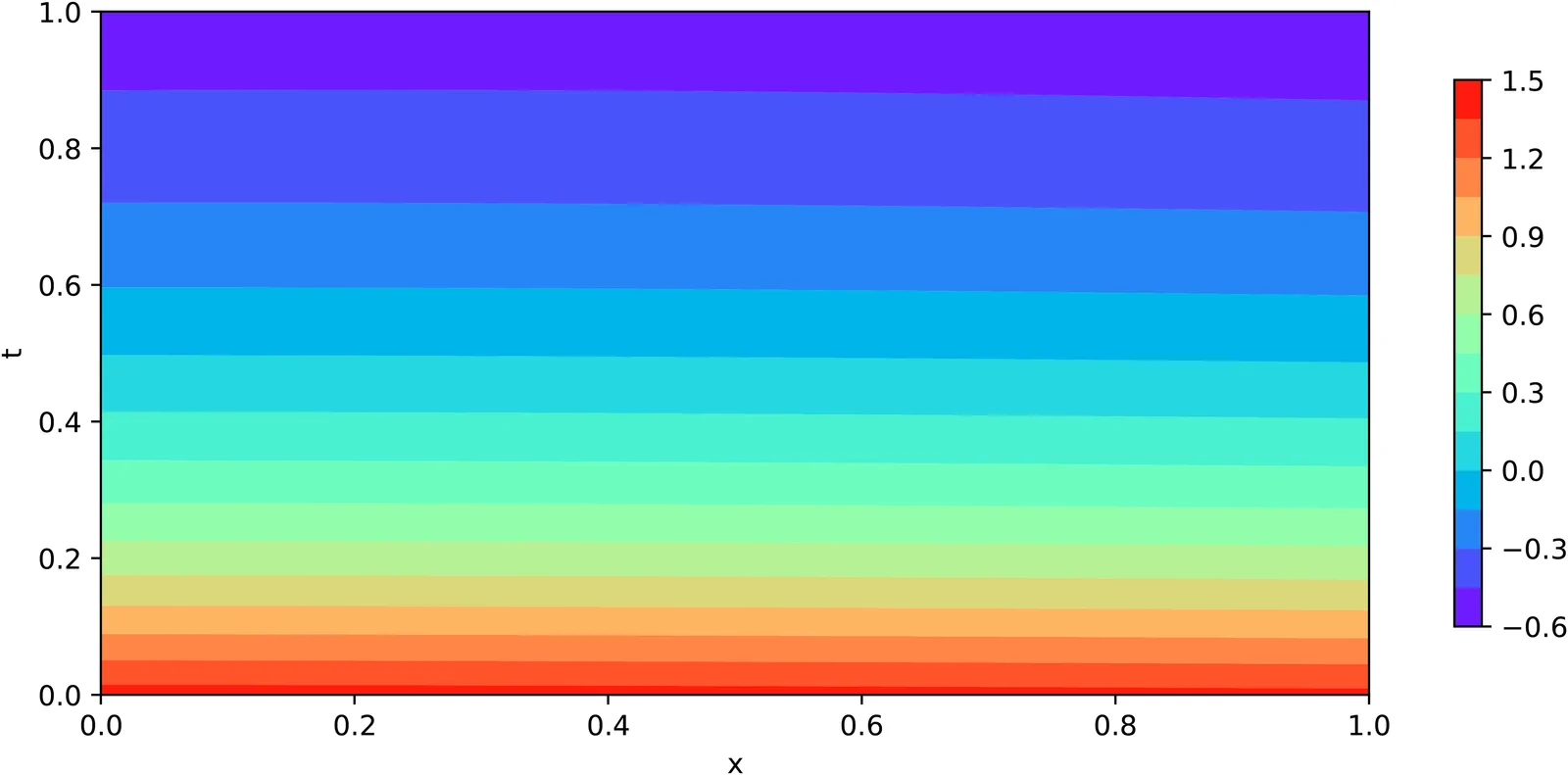
Contour solution comparison for Case IV. S-PINN contour.

**Fig 62:**
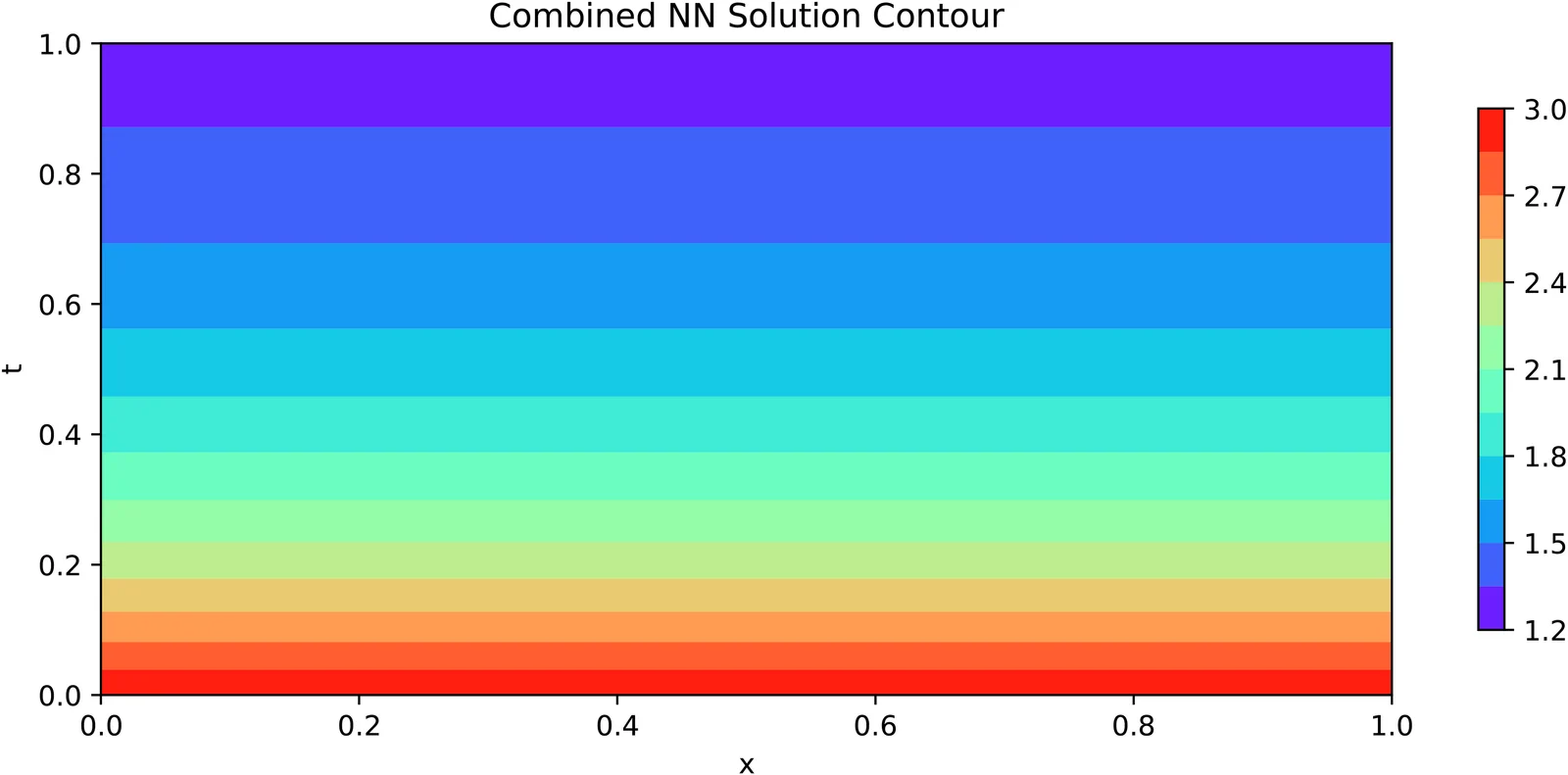
Contour solution comparison for Case IV. PINN contour.

The exact surface in Fig 57 shows a smooth and continuous solution pattern. The variation of the surface is physically consistent, without artificial jumps, noisy ridges, or unwanted flattening. The corresponding exact contour plot in Fig 60 shows well-organized contour bands, which represent the natural structure of the analytical solution. These exact plots serve as the reference for evaluating the two neural-network-based approximations.

The S-PINN surface in Fig 58 reproduces the main structure of the exact surface with strong visual agreement. The curvature, surface orientation, and smooth transition of the solution are better preserved than in the standard PINN result. This shows that the symmetry constraint helps the model learn the global shape of the solution, not only isolated pointwise values. Similarly, the S-PINN contour plot in Fig 61 maintains a regular and coherent contour arrangement. The contour lines follow the same general direction and spacing pattern as the exact solution, confirming that the proposed model preserves the geometry of the reduced system.

In contrast, the standard PINN surface in Fig 59 captures the broad trend of the solution but shows weaker agreement with the exact surface. Some regions appear less curved or slightly flattened, which indicates that the standard PINN has difficulty preserving the full solution geometry. This limitation is also visible in Fig 62, where the contour lines are less regular and show stronger distortion compared with the exact and S-PINN contours. These differences indicate that the standard PINN may satisfy the residual approximately, but it does not fully preserve the invariant structure associated with the reduced problem.

The improved surface and contour patterns of the S-PINN are consistent with recent developments in structure-preserving scientific machine learning. Classical PINNs use the PDE residual as a soft physical constraint, but they do not automatically encode the symmetry or invariant geometry of the governing equation. This can lead to physically admissible-looking but geometrically less accurate solutions. Symmetry-informed PINN approaches address this issue by adding invariant constraints or Lie-symmetry information into the training objective. The present results confirm that this strategy improves the global reconstruction of the solution field in Case IV.

#### 5.3.7 Loss components.

Figs 63 and 64 decomposes the S-PINN objective function into PDE-related and symmetry-related loss components. This decomposition is useful because the total loss alone does not explain how the model balances the different physical and structural requirements during training. In PINN-type methods, the total loss usually contains several terms, and poor balance among them may cause the model to satisfy one constraint while neglecting another.

**Fig 63:**
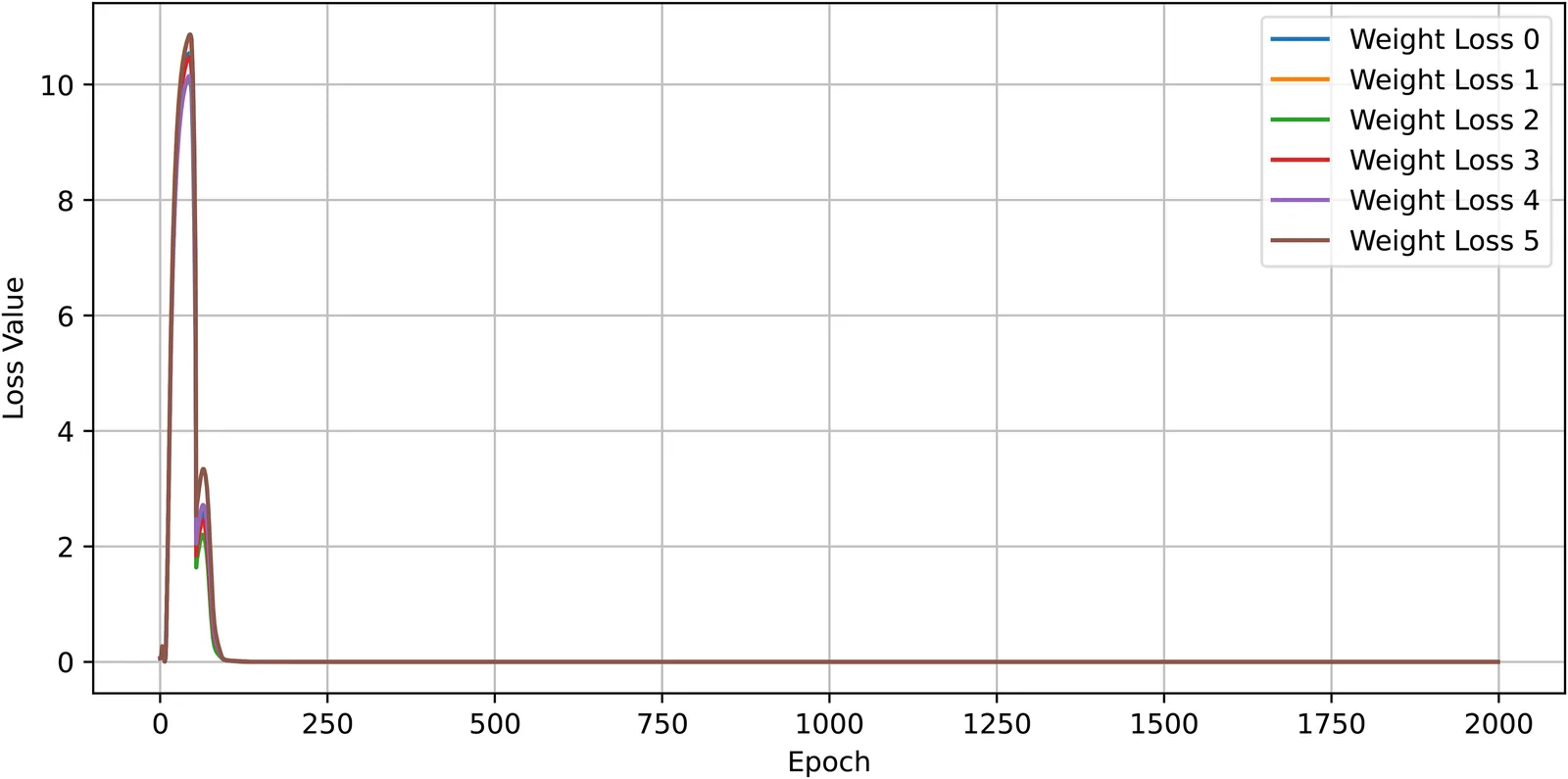
S-PINN loss component analysis for Case IV. PDE loss components.

**Fig 64:**
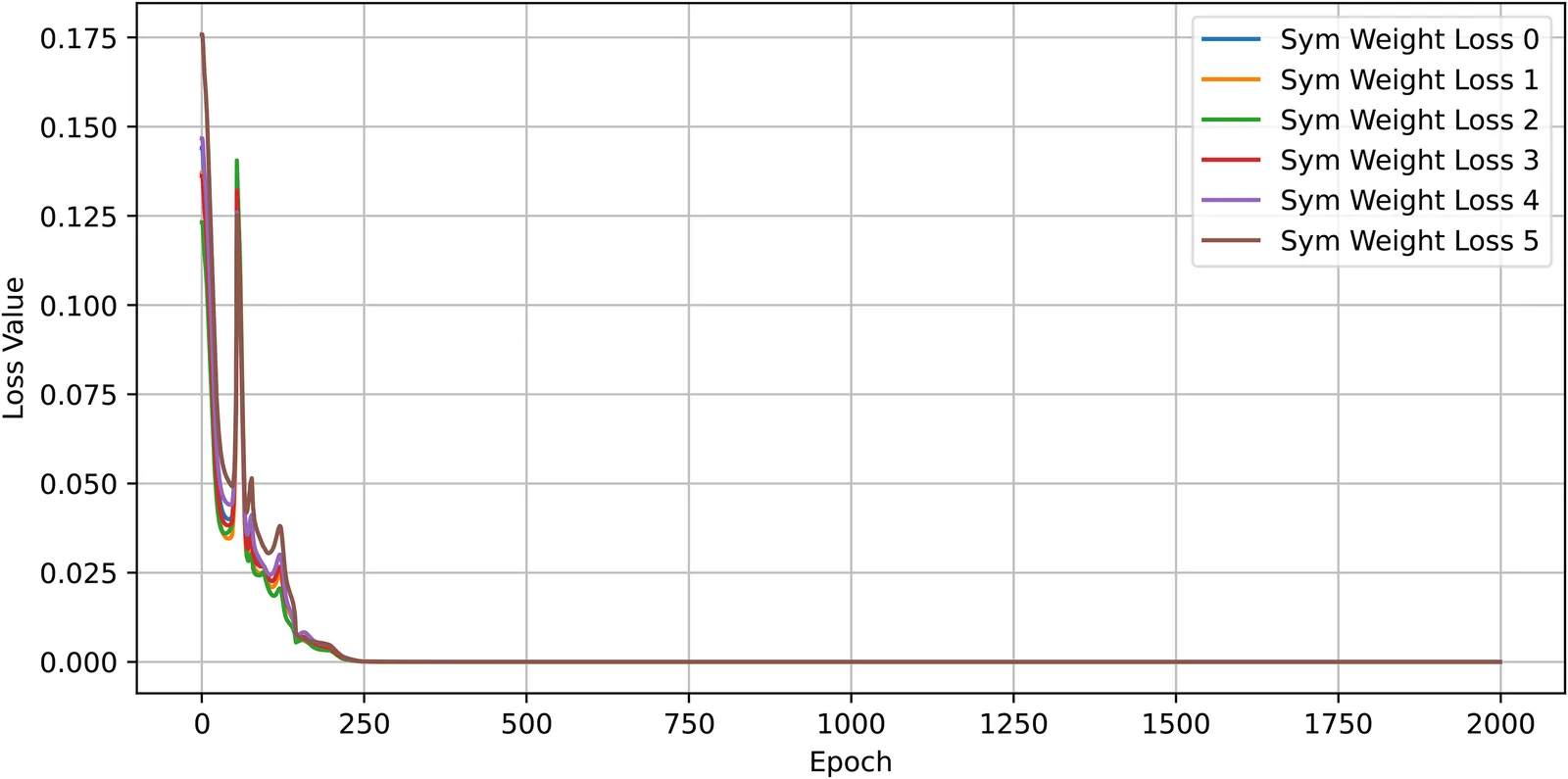
S-PINN loss component analysis for Case IV. Symmetry loss components.

The PDE loss components in Fig 63 show a coordinated decreasing trend during training. This means that the network learns the governing equation in a balanced way. No individual PDE residual remains dominant for a long part of the training process, which indicates that the model is not overfitting one component of the equation while ignoring the others. This balanced behavior supports the solution accuracy observed in the ODE, surface, and contour comparisons.

The symmetry loss components in Fig 64 show a rapid decrease during the early epochs, followed by a slower refinement stage. This behavior has a clear interpretation. At the beginning of training, the network quickly learns the main invariant structure imposed by the symmetry constraint. After this initial stage, the optimizer makes smaller corrections to improve the precision of symmetry enforcement. In other words, the S-PINN first learns the broad symmetry-compatible solution family and then refines the solution to satisfy both the PDE residual and the symmetry condition more accurately.

This result directly addresses one of the major difficulties reported in previous PINN studies. Standard PINNs can suffer from gradient imbalance because different loss components may generate gradients with different magnitudes. Their later NTK-based analysis also showed that different loss terms may converge at different rates, which can slow training and reduce accuracy. In the present S-PINN, the symmetry constraint helps organize the learning process by reducing the effective solution space and guiding the optimizer toward physically meaningful directions. Therefore, the balanced decay of the PDE and symmetry losses confirms that the proposed model improves the training dynamics compared with standard PINNs.

#### 5.3.8 Weight Evolution.

Figs 65 and 66 shows the evolution of the trainable weights associated with the PDE and symmetry components of the S-PINN model. The weight history provides insight into the internal learning dynamics of the network. Stable weight evolution usually indicates a well-conditioned optimization process, while persistent large oscillations may indicate conflict between different loss terms or instability in the training process.

**Fig 65:**
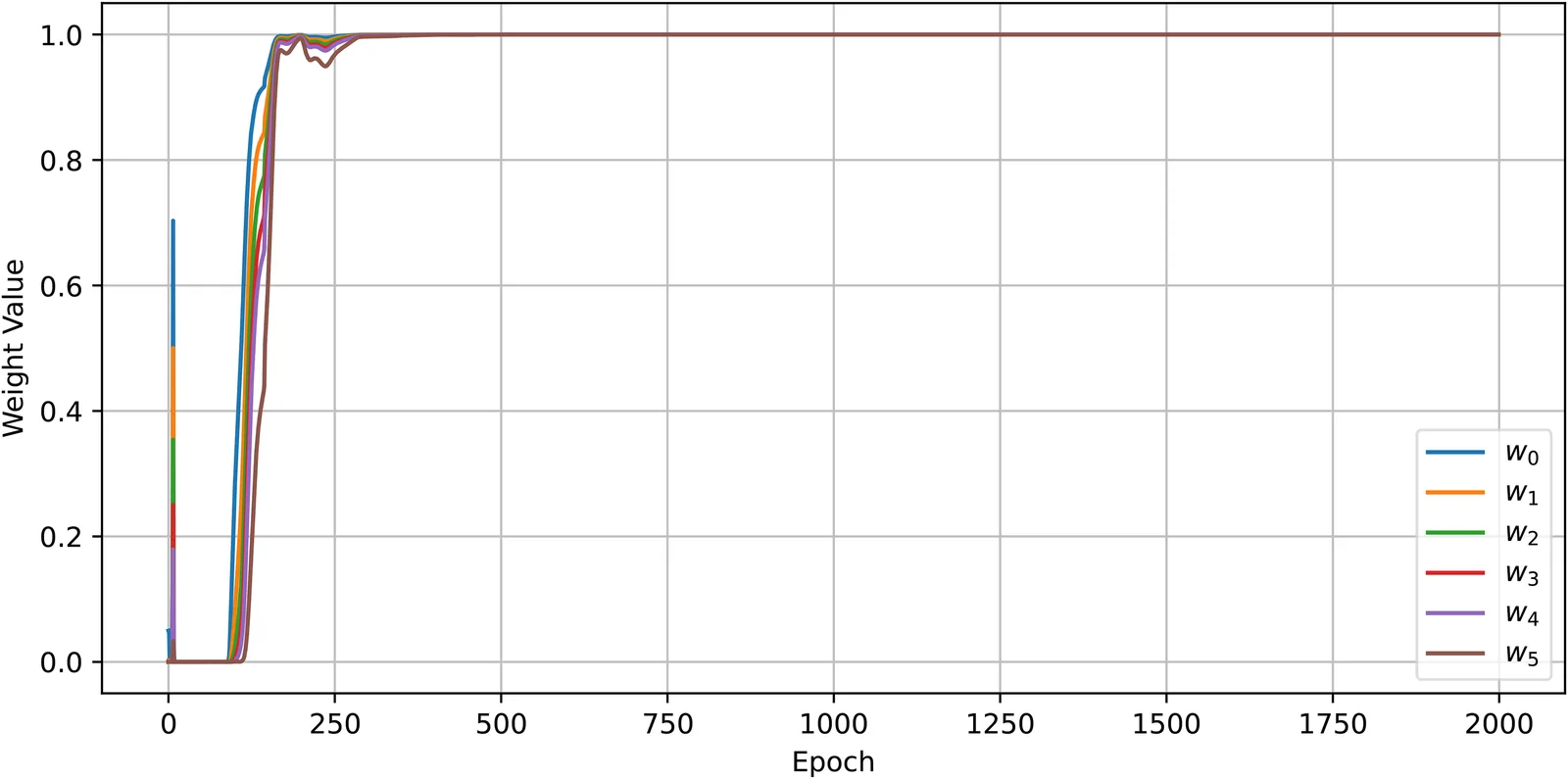
Weight evolution during S-PINN training for Case IV. PDE network weights.

**Fig 66:**
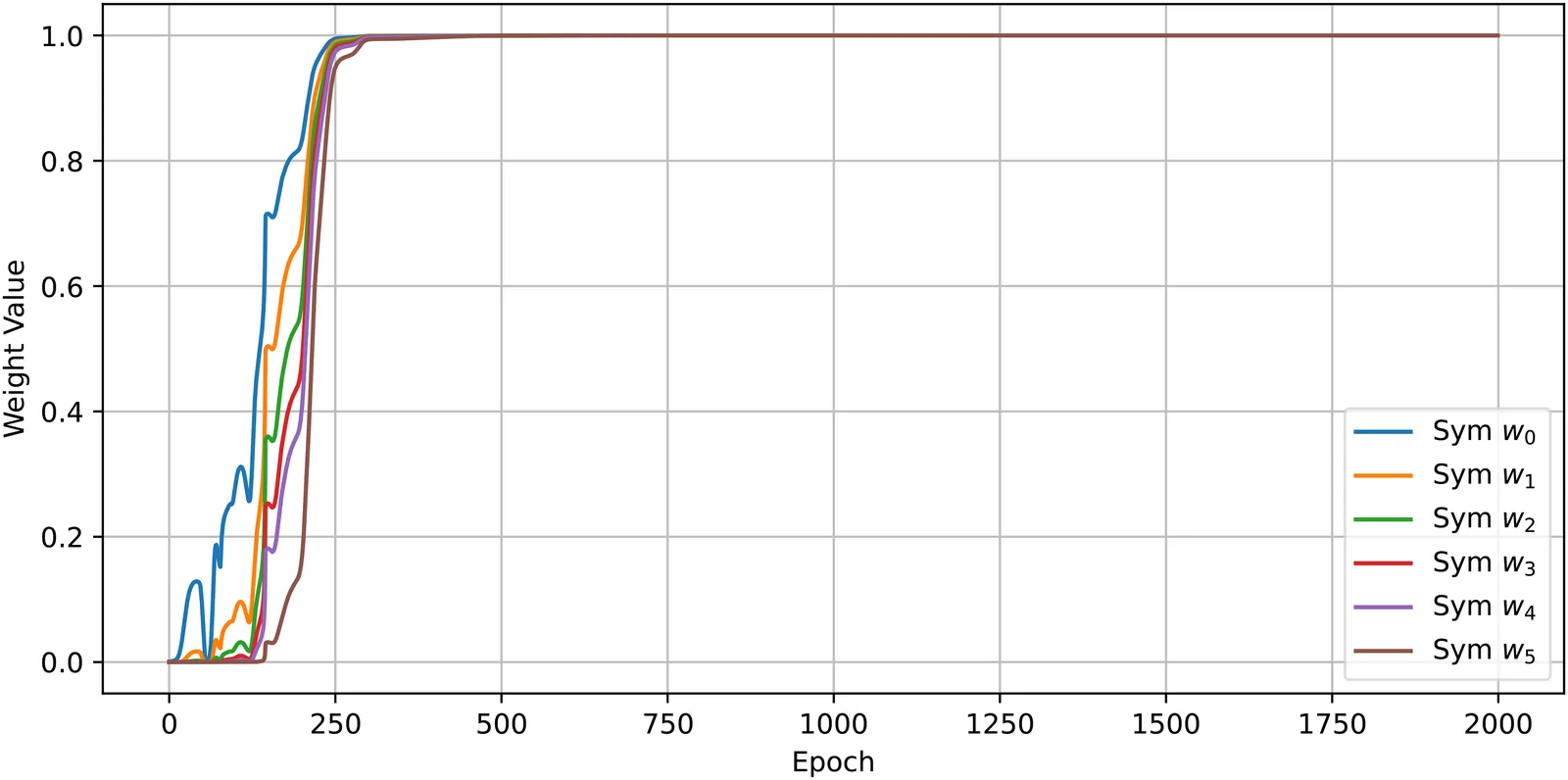
Weight evolution during S-PINN training for Case IV. Symmetry network weights.

The PDE network weights in Fig 65 follow a smooth and gradual convergence pattern. This indicates that the network learns the governing equation residual in a stable manner. The absence of strong oscillatory behavior suggests that the PDE component of the loss is being minimized without severe instability. This is consistent with the balanced PDE loss reduction shown earlier in Fig 63.

The symmetry network weights in Fig 66 show mild oscillations during the early training stage and then move toward a more stable regime. This behavior is expected because the network initially has to balance two tasks: learning the reduced solution and enforcing the symmetry structure. The early oscillations represent the adjustment stage where the model searches for a suitable balance between these two requirements. Once the symmetry structure is learned, the weights stabilize, showing that the symmetry component begins to act as a regularizing mechanism.

This stable weight evolution explains why the S-PINN produces better solution profiles and smoother global solution fields. The symmetry weights help guide the model away from physically inconsistent approximations and toward solutions that respect the invariant structure of the reduced equation. This is consistent with Lie-symmetry-based neural solvers, where the incorporation of symmetry generators improves learning by exposing the network to neighboring symmetry-related solutions. Therefore, the weight evolution results show that the proposed S-PINN improves not only the final accuracy but also the stability and interpretability of the training process.

## 6 Conclusion

This study developed a causality-enhanced and symmetry-preserving Physics-Informed Neural Network framework for nonlinear elastic wave equations with viscous damping. By combining Lie symmetry reductions, symmetry-aware loss functions, and causality-based temporal weighting, the proposed S-PINN framework constrains the neural solution to physically admissible structures and improves the stability of learning for nonlinear dissipative wave systems.

The numerical results show that S-PINNs provide faster convergence, lower loss values, more stable gradient behavior, and better agreement with exact solutions compared with standard PINNs. These improvements confirm that embedding symmetry information and temporal causality into the training process is an effective strategy for improving the accuracy and physical consistency of neural solvers for elastodynamic problems.

The findings offer useful insights for researchers and engineers working in computational mechanics, material modeling, nondestructive testing, biomedical ultrasound, and soft-material analysis. The proposed framework can serve as a reliable mesh-free tool for simulating damped nonlinear wave propagation while preserving the mathematical structure of the governing equations.

Future work extend this framework to two- and three-dimensional elastodynamic models, more realistic viscoelastic or anisotropic materials, and inverse problems for estimating damping coefficients and elastic parameters. Further improvements may also include adaptive causality weighting, residual-based sampling, experimental data integration, and uncertainty quantification to enhance robustness for practical engineering applications.
